# RSK1 promotes mammalian axon regeneration by inducing the synthesis of regeneration-related proteins

**DOI:** 10.1371/journal.pbio.3001653

**Published:** 2022-06-01

**Authors:** Susu Mao, Yuanyuan Chen, Wei Feng, Songlin Zhou, Chunyi Jiang, Junjie Zhang, Xiaohong Liu, Tianmei Qian, Kai Liu, Yaxian Wang, Chun Yao, Xiaosong Gu, Bin Yu

**Affiliations:** 1 Key Laboratory of Neuroregeneration of Jiangsu and Ministry of Education, NMPA Key Laboratory for Research and Evaluation of Tissue Engineering Technology Products, Co-innovation Center of Neuroregeneration, Nantong University, Nantong, China; 2 Division of Life Science, State Key Laboratory of Molecular Neuroscience, The Hong Kong University of Science and Technology, Hong Kong, China; 3 Jiangsu Clinical Medicine Center of Tissue Engineering and Nerve Injury Repair, Affiliated Hospital of Nantong University, Nantong University, Nantong, China; University of Notre Dame, Center for Stem Cells and Regenerative Medicine, UNITED STATES

## Abstract

In contrast to the adult mammalian central nervous system (CNS), the neurons in the peripheral nervous system (PNS) can regenerate their axons. However, the underlying mechanism dictating the regeneration program after PNS injuries remains poorly understood. Combining chemical inhibitor screening with gain- and loss-of-function analyses, we identified p90 ribosomal S6 kinase 1 (RSK1) as a crucial regulator of axon regeneration in dorsal root ganglion (DRG) neurons after sciatic nerve injury (SNI). Mechanistically, RSK1 was found to preferentially regulate the synthesis of regeneration-related proteins using ribosomal profiling. Interestingly, RSK1 expression was up-regulated in injured DRG neurons, but not retinal ganglion cells (RGCs). Additionally, RSK1 overexpression enhanced phosphatase and tensin homolog (PTEN) deletion-induced axon regeneration in RGCs in the adult CNS. Our findings reveal a critical mechanism in inducing protein synthesis that promotes axon regeneration and further suggest RSK1 as a possible therapeutic target for neuronal injury repair.

## Abstract 2

This study shows that p90 ribosomal S6 kinase 1 (RSK1) responds differentially to nerve injury in the peripheral and central nervous systems, and identifies it as a crucial regulator of axonal regeneration; mechanistically, RSK1 preferentially induces the synthesis of regeneration-related proteins via the RSK1-eEF2K-eEF2 axis.

## Introduction

Successful axon regeneration will be of benefit in treating many human diseases involving axon damage, such as traumatic brain injury, stroke, spinal cord injury, sciatic nerve injury (SNI), and numerous neurodegenerative diseases, in both the central nervous system (CNS) and peripheral nervous system (PNS) [1–3]. In contrast to the CNS, injured neurons in the PNS have robust regenerative capability, which largely depends on the up-regulation of the expression or activity of key molecules that promote axon regeneration following injury [4–7]. Hence, uncovering the molecular mechanisms underpinning axon regrowth following neuronal injury in the PNS will greatly aid the understanding of the differential regenerative capacity between neurons in the PNS and CNS and contribute to identifying factors with the potential to facilitate nerve regeneration.

Axon regeneration is a highly synergistic process consisting of various cellular events, including injury signal sensing, axon cargo transport, cytoskeletal organization, cellular energy homeostasis, and the synthesis of macromolecules [8–10]. Long-distance axon regeneration requires the sustained activity of structural and regulatory proteins in both the axon and the soma [11–13]. Several recent studies have implicated translational repression and ribosome biogenesis as rate-limiting processes for axon or dendrite growth [14,15] and identified that changes in the balance between protein synthesis in the axon and the soma affect axonal growth rates [16]. These findings indicate that manipulating protein synthesis has the potential to improve axonal regenerative ability.

Protein synthesis consists of 3 phases, namely, initiation, elongation, and termination. Each step involves a number of protein factors extrinsic to the ribosome [17]. Increasing evidence has indicated that mechanistic target of rapamycin (mTOR) is the central mediator of protein synthesis, controlling several components involved in the initiation and elongation stages of translation, such as EIF4B and eEF2[18,19]. There is also ample evidence supporting that mTOR plays a dominant role in axon regeneration in CNS neurons [20,21], whereas its effect on axon regeneration in PNS neurons is relatively limited. As previous studies have shown, inhibiting mTOR activity with rapamycin does not affect dorsal root ganglion (DRG) neuron axon regrowth although S6K1 activation is blocked [22,23], indicating that other signaling pathways are involved in the synthesis of proteins required for PNS nerve regeneration. In addition to mTOR, the mitogen-activated protein kinases (MAPKs) signaling pathway is known to be one of the best understood regulators of mRNA translation [19]. Known substrates of MAPKs include members of a family of Ser/Thr kinases, known as MAPK-activated protein kinases (MAPKAPKs) [24,25], among which the p90 ribosomal S6 kinases (RSKs) and the MAPK-interacting kinases (MNKs) have been directly implicated in the regulation of mRNA translation [26,27].

The RSK family is composed of a group of highly conserved Ser/Thr kinases, in which 4 RSK genes have been identified (RSK1, RSK2, RSK3, and RSK4) in mammals. RSKs were the first protein kinases found to have a 2-kinase domain structure, an N-terminal kinase (NTK) domain and a carboxyl-terminal kinase (CTK) domain, which are separated by a linker region that contains a hydrophobic motif [28]. Through the phosphorylation events in CTK domain, linker region, and NTK domain in sequence, RSKs are activated and able to phosphorylate downstream cellular targets to regulate diverse cellular processes, such as cell growth, cell motility, cell survival, and cell proliferation [29]. Besides, RSKs were found to modulate the activity of components of the translational machinery, such as ribosomal protein S6 and translational elongation factor eEF2, to affect protein synthesis [30,31]. However, the role of RSKs in axon regeneration remains elusive. Here, we find the expression level and activity of RSK1 in DRG neurons are significantly elevated by SNI and present evidence that RSK1 is an important regulator of axon regeneration, mainly through the induction of regeneration-related protein synthesis.

## Results

### The RSK inhibitors suppress neurite regrowth in DRG neurons

**Fig 1:**
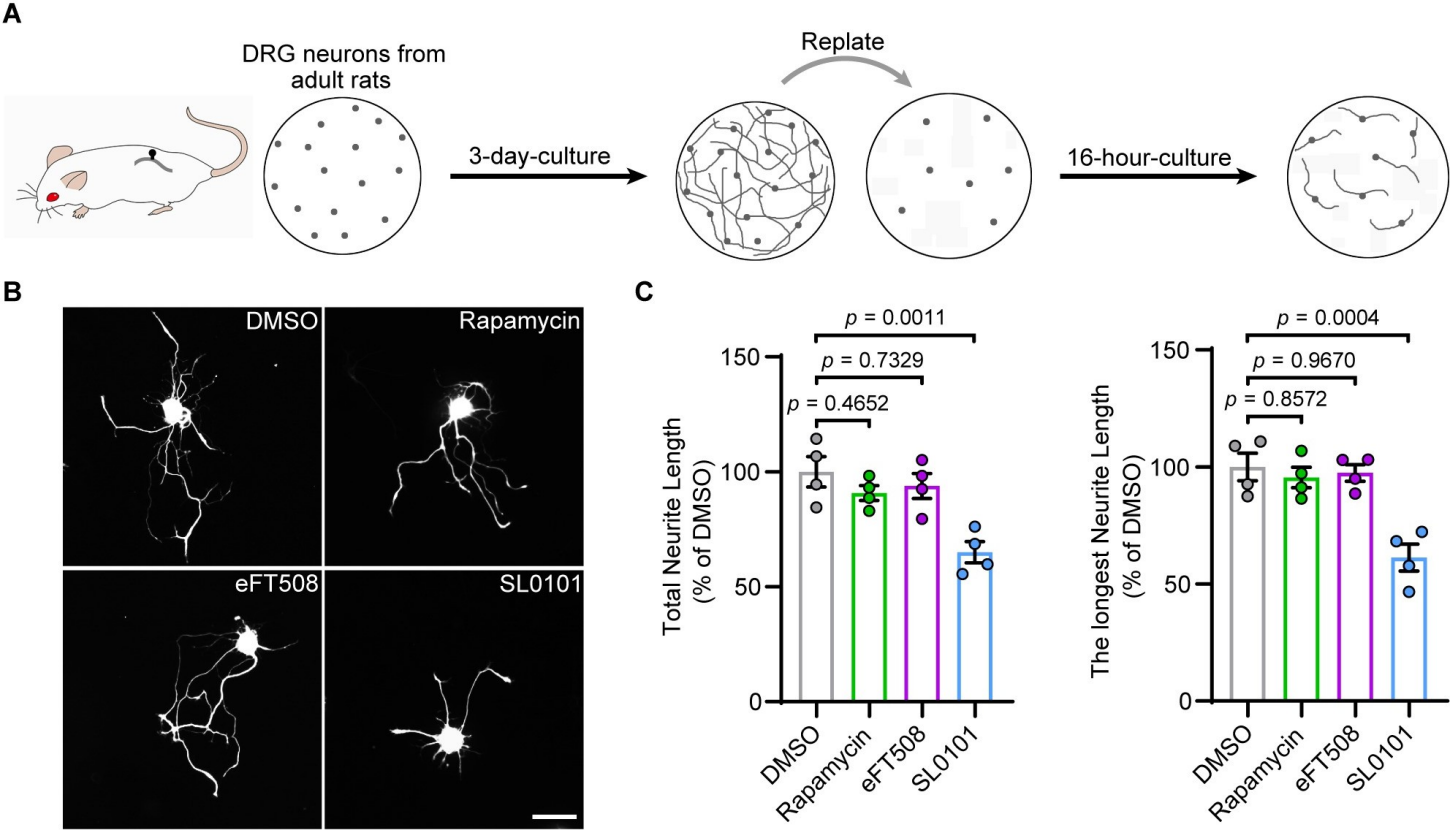
SL0101 inhibits DRG neuron regenerative growth. **(A)** Schematic of the culture-and-replate protocol. DRG neurons were dissociated from adult rats and cultured for 3 days. Neurons were then replated to reinitiate axon growth. Regenerative axon growth was assessed by measuring axon length in replated neurons 16 hours after replating. **(B)** Representative images of cultured DRG neurons treated with DMSO, 50 nM rapamycin, 1 μM eFT508, and 10 μM SL0101. Scale bar, 50 μm. **(C)** Quantification of the total and the longest neurite outgrowth per neuron relating to (B) (mean ± SEM, 1-way ANOVA, Dunnett post hoc test, *n* = 4 biologically independent experiments, approximately 50 cells/experiment on average). The data underlying all the graphs shown in the figure are included in S1 Data. DRG, dorsal root ganglion; SEM, standard error of the mean.

Although many signaling molecules intersect to control protein synthesis, mTOR, MNK, and RSK appear to be key players [32]. To determine the dominant regulator of mRNA translation during DRG neuron axon regrowth, we employed an in vitro neurite regrowth assay (Fig 1A) that can recapitulate in vivo axon regeneration induced by peripheral axotomy [23]. Replated DRG neurons were treated with various concentrations of the small-molecule inhibitors rapamycin, eFT508, or SL0101, which inhibit mTOR, MNK1/2, and RSKs, respectively. As determined by CCK-8 assay, cell viability was not significantly affected when cells were treated for 24 hours with up to 200 nM rapamycin, 10 μM eFT508, or 100 μM SL0101, indicating that these inhibitors were not toxic to the DRG neurons under our experimental conditions (S1A Fig). Next, we treated replated neurons with moderate concentrations of the inhibitors (50 nM rapamycin, 1 μM eFT508, or 10 μM SL0101) and found that treatment with the pan-RSK inhibitor SL0101 significantly decreased the total and the longest neurite length of the neurons, whereas rapamycin or eFT508 had no significant effect (Fig 1B and 1C), in line with a previous result showing that DRG neurite outgrowth is resistant to rapamycin [23]. To further confirm the role of RSKs in axon regrowth, we used a second RSK inhibitor (BI-D1870) [33]. Our results showed that the application of BI-D1870 alone, or as a combination with SL0101 (S1B Fig), significantly suppressed neurite regrowth in replated DRG neurons (S1C and S1D Fig). These data suggested that RSKs might be essential for axon regrowth in DRG neurons.

### RSK1 expression and activity are up-regulated in DRG neurons following SNI

**Fig 2:**
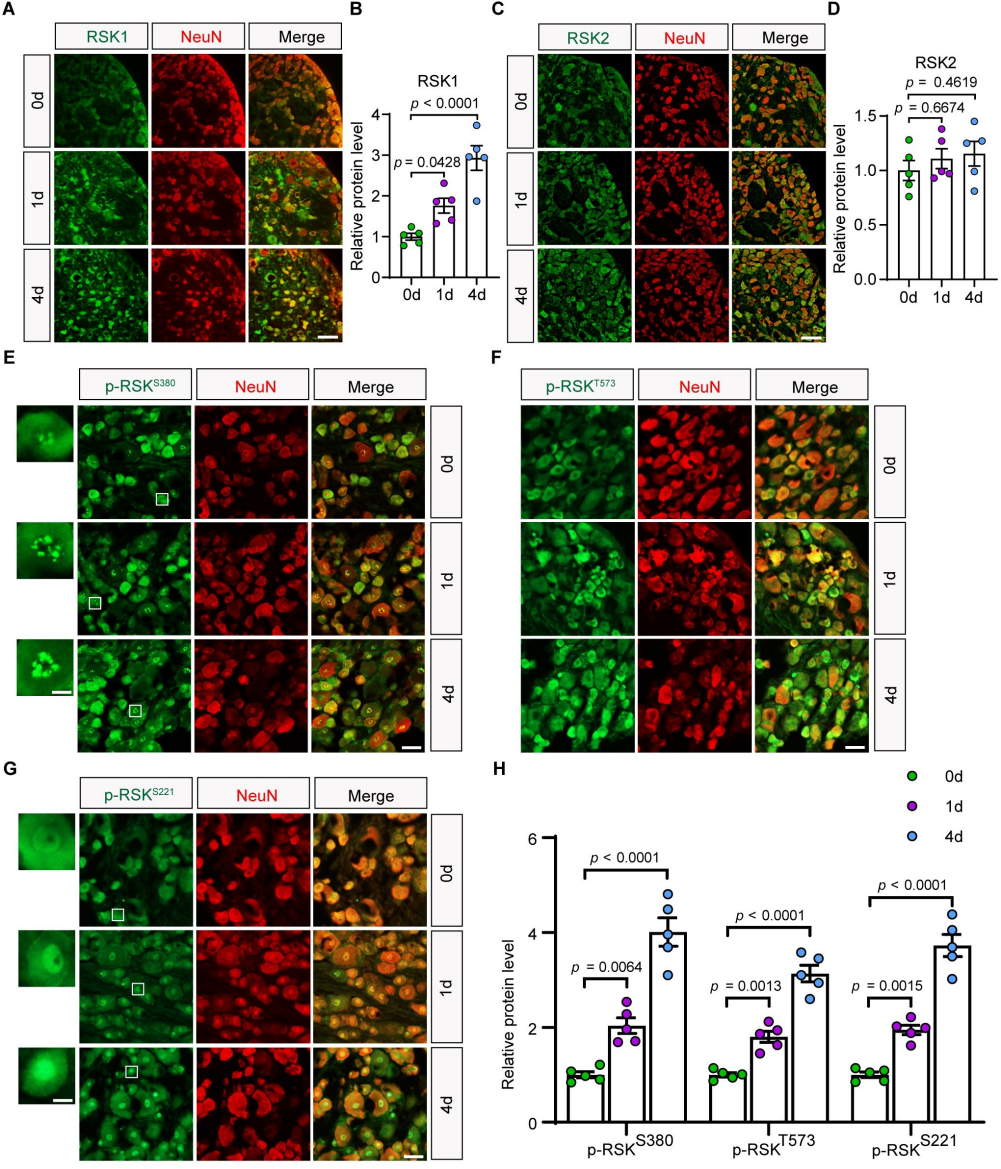
RSK1 expression and phosphorylation are up-regulated in DRG neurons following sciatic nerve axotomy. **(A, C)** Representative fluorescence images of immunostaining for RSK1 (A) and RSK2 (C) in the DRG on day 0, 1, or 4 post-SNI. Scale bar, 100 μm. **(B, D)** Quantification of RSK1 (B) and RSK2 (D) immunofluorescence intensity relating to (A) and (C), respectively. Relative protein expression levels were quantified after normalization to background immunofluorescence (secondary antibody only) (mean ± SEM, 1-way ANOVA, Dunnett post hoc test, *n* = 5 biologically independent animals/group). **(E–G)** Representative fluorescence images of immunostaining for p-RSK^S380^ (E), p-RSK^T573^ (F), and p-RSK^S221^ (G) in the DRG on day 0, 1, or 4 post-SNI. Enlarged views of the boxed regions in (E) and (G) are shown on the left of each panel. Scale bar, 50 μm in lower magnification view and 10 μm in higher magnification view. **(H)** Quantification of p-RSK^S380^ immunofluorescence intensity in the nuclei (E), p-RSK^T573^ immunofluorescence intensity in the soma (F), and p-RSK^S221^ immunofluorescence intensity in the nuclei (G). Relative p-RSK expression levels were quantified after normalization to background immunofluorescence (secondary antibody only) (mean ± SEM, 1-way ANOVA, Dunnett post hoc test, *n* = 5 biologically independent animals/group). The data underlying all the graphs shown in the figure are included in S1 Data. DRG, dorsal root ganglion; RSK, ribosomal S6 kinase; RSK1, ribosomal S6 kinase 1; SEM, standard error of the mean; SNI, sciatic nerve injury.

Studies have shown that the expression levels of many regeneration-associated genes are significantly changed when the sciatic nerve spontaneously regenerates after injury [34,35]. To identify which member(s) of the RSK family is required for axon regeneration in DRG neurons, we examined changes of expression and phosphorylation levels of individual RSKs following the SNI. In situ hybridization analysis showed that within 4 RSKs, only mRNA level of RSK1 was significantly increased in the DRG at day 4 compared to that at day 0 post-SNI (S2A and S2B Fig). Western blotting revealed that the protein level of RSK1, but not RSK2, was increased in DRGs post-SNI (S2C and S2D Fig). Next, we performed immunohistochemistry (IHC) to determine expressional changes of RSK1 in DRG neurons versus other cell types. We observed that most of the RSK1 signals were colocalized with those of a neuronal cell marker NeuN (Fig 2A) and that the protein level of RSK1 in DRG neuronal soma was significantly up-regulated at days 1 and 4 compared with that at day 0 post-SNI (Fig 2A and 2B). In contrast, the expression of RSK2 in DRG neuronal soma showed no overt change (Fig 2C and 2D).

Six different phosphorylation sites have been mapped in RSK1, of which Ser221 (S221), Ser380 (S380), and Thr573 (T573) have been reported to be important for RSK1 activity [29]. We found that the levels of phosphorylation at these sites were increased at days 1 and 4 post-SNI (Fig 2E–2H, S2E and S2F Fig). In addition, the localization of p-RSK^S380^ and p-RSK^S221^ in the nuclei indicates that RSK1 was activated in DRG neurons post-SNI (Fig 2E and 2G). In light of this, the western blotting analysis of nuclear versus cytoplasmic fractions of DRG tissue revealed that there were more p-RSK^S380^ and p-RSK^S221^ in nuclei at days 1 and 4 compared with that at day 0 post-SNI (S2G and S2H Fig). To further investigate whether phosphorylated RSK is functioning to enhance protein synthesis, we examined the phosphorylation of S6 and eEF2K, 2 well-known substrates of RSK related to mRNA translation [30,31]. We observed that the level of p-S6^S235/236^ was slightly increased in DRG post-SNI (S3A, S3B, S3E and S3F Fig), while that of p-eEF2K was significantly increased (S3C–S3E and S3G Fig). These results indicated that RSK1 activation was injury induced and suggested that it may play a role in protein synthesis involved in PNS regeneration.

### RSK1 is a facilitator of axon regeneration and functional recovery of DRG neurons after injury

**Fig 3:**
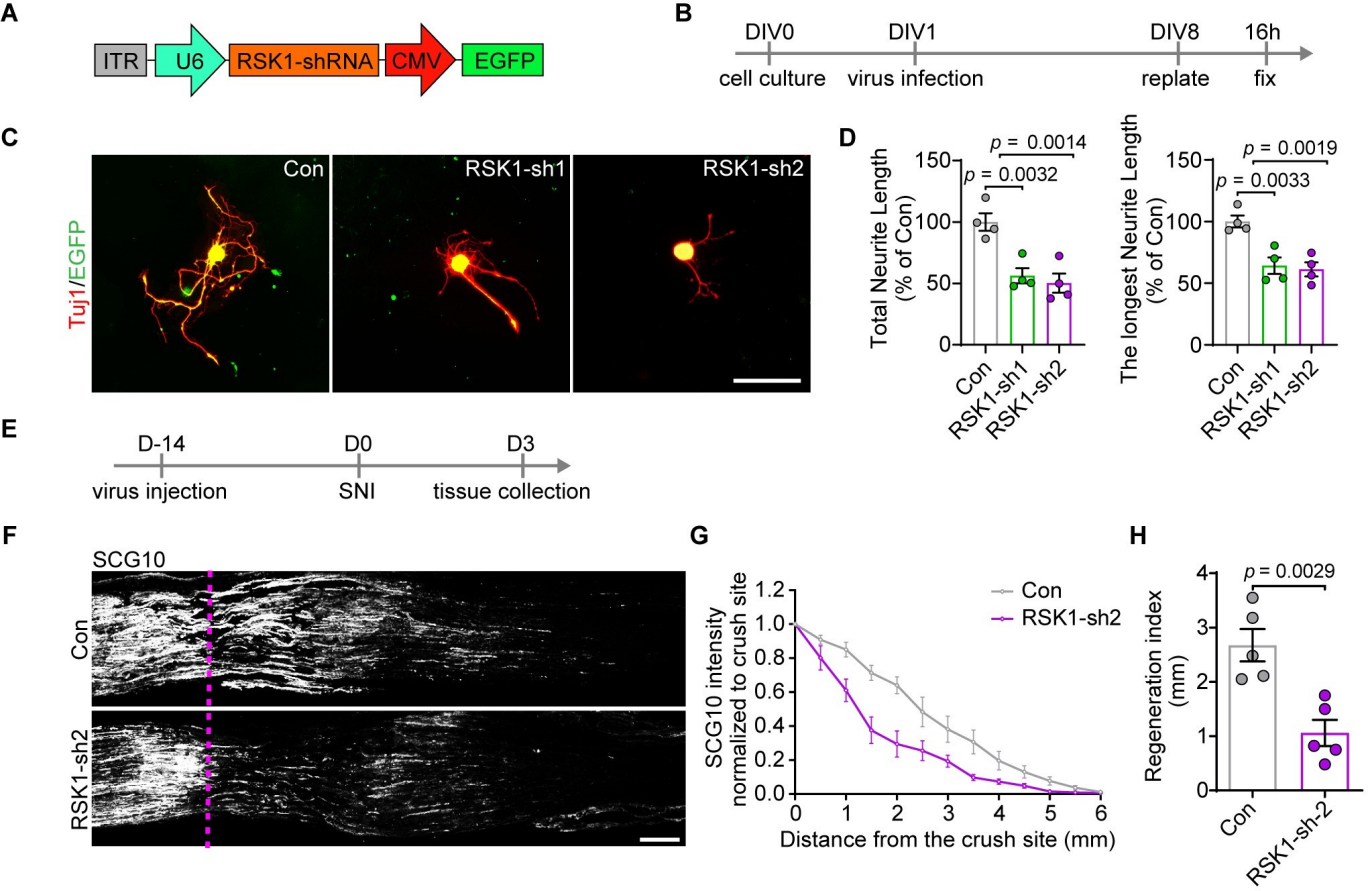
KD of RSK1 inhibits axon regeneration in vitro and in vivo. **(A)** A schematic representation of the AAV2/8-U6-shRNA-CMV-EGFP vector utilized in RSK1 KD experiments. **(B)** Timeline for RSK1 KD in DRG neurons at DIV1. **(C)** Representative images of cultured DRG neurons (EGFP and Tuj1 double positive neurons) infected with control AAV2/8 expressing scramble shRNA or AAV expressing shRNA1 (RSK1-sh1) or RSK1-sh2 to knock down RSK1. Scale bar, 100 μm. **(D)** Quantification of the total and longest neurite outgrowth per neuron relating to (C) (mean ± SEM, 1-way ANOVA, Dunnett post hoc test, *n* = 4 biologically independent experiments, approximately 50 cells/experiment on average). **(E)** Timeline for RSK1 KD in vivo and SNI. **(F)** Representative longitudinal sections from injured sciatic nerves. The crush site is indicated by a purple dotted line. Scale bar, 500 μm. **(G)** Normalized SCG10 intensity plotted in function of the distance from the crush line (*n* = 5 rats per group). **(H)** Axon regeneration in injured rats was quantified by regeneration indices obtained from SCG10 immunostaining on day 3 after crush injury (mean ± SEM, unpaired 2-tailed *t* test, *n* = 5 rats per group). The data underlying all the graphs shown in the figure are included in S1 Data. DIV1, 1 day in vitro; DRG, dorsal root ganglion; KD, knockdown; RSK1, ribosomal S6 kinase 1; SEM, standard error of the mean; SNI, sciatic nerve injury.

Given the limitation of RSK inhibitors we used, such as the off-target effects and negative feedback pathways functioning through RSK and related kinases [36,37], we went further to knock down RSK1 expression using RSK1-specific shRNA (Fig 3A) and examined its effect on DRG axon regeneration (Fig 3B). DRG cultures were infected with AAV2/8 that carry shRNAs against RSK1, at 1 day in vitro (DIV1). Seven days later, the reverse transcription quantitative real-time PCR (RT-qPCR) and western blotting analysis revealed that the RNA and protein levels of RSK1, but not that of RSK2, were significantly reduced by shRNA1 (RSK1-sh1) and RSK1-sh2 (S4A–S4C Fig). The neurite regrowth assay showed that RSK1 knockdown (KD) by either shRNA reduced the total and the longest neurite length of DRG neurons (Fig 3C and 3D). Among them, RSK1-sh2 was shown to have a stronger effect on reducing RSK1 expression and neurite length and thus was chosen for further experiments. To rule out the possibility of potential off-target effects, we examined the expression of several predicted off-target candidates (exocyst complex component 2, calcium voltage-gated channel subunit alpha1 S, and protein kinase C alpha) upon RSK1-sh2 treatment. RT-qPCR analysis revealed that RSK1-sh2 had no inhibition effect on above-mentioned candidate genes (S4D Fig), confirming the specificity of RSK1-sh2.

Next, we tried to assess the in vivo effect of RSK1 KD on axon regeneration in adult DRG neurons via intrathecal injection of AAV2/8-RSK1-sh2 in animals with SNI (Fig 3E). In intact rats receiving AAV2/8-scrambled shRNA-EGFP, we observed that 36.45 ± 3.54% of DRG neurons were successfully infected (S5A and S5B Fig). Among them, NF200^+^, CGRP^+^ and IB4^+^ neurons were infected with varying degrees (S5C–S5F Fig), indicating that we are primarily infecting myelinated larger DRG neurons, such as mechanoreceptors and proprioceptors and not the predominant fraction of nociceptors of the DRG. Intrathecal injection of AAV-2/8-RSK1-sh2 significantly down-regulated RSK1 expression level in DRG neurons in vivo (S5G and S5H Fig). Next, at 3 days after SNI, a marker of regenerating sensory axon SCG10 [38] was used to identify the regenerating sciatic nerve extending from the DRG neurons. We found that the extension of SCG10^+^ axons was substantially repressed in RSK1 KD rats compared with that in animals injected with control shRNA (Fig 3F and 3G). We calculated a regeneration index by measuring the distance from the crush site at which the average SCG10 intensity was half that observed at the crush site [39]. The regeneration index was significantly lower in nerves treated with RSK1-sh2 compared with those treated with control shRNA (Fig 3H). These observations confirmed that RSK1 is required for axon regeneration in DRG neurons.

Since the in vitro neurite regrowth model really mimics the conditioning injury paradigm where the initial dissociation serves as the conditioning injury, we asked whether RSK1 is important in the conditioning injury effect in vivo. We performed AAV intrathecal injection and sciatic nerve transection (the first injury) simultaneously. Fourteen days later, we performed a crush injury (the second injury) and assessed sciatic nerve regrowth 2 days later (S6A Fig). The immunostaining of SCG10 showed that, with a conditioning injury, suppressing RSK1 significantly inhibited sciatic nerve regrowth postinjury (S6B–S6D Fig). These data confirmed that RSK1 is important in the conditioning injury effect.

**Fig 4:**
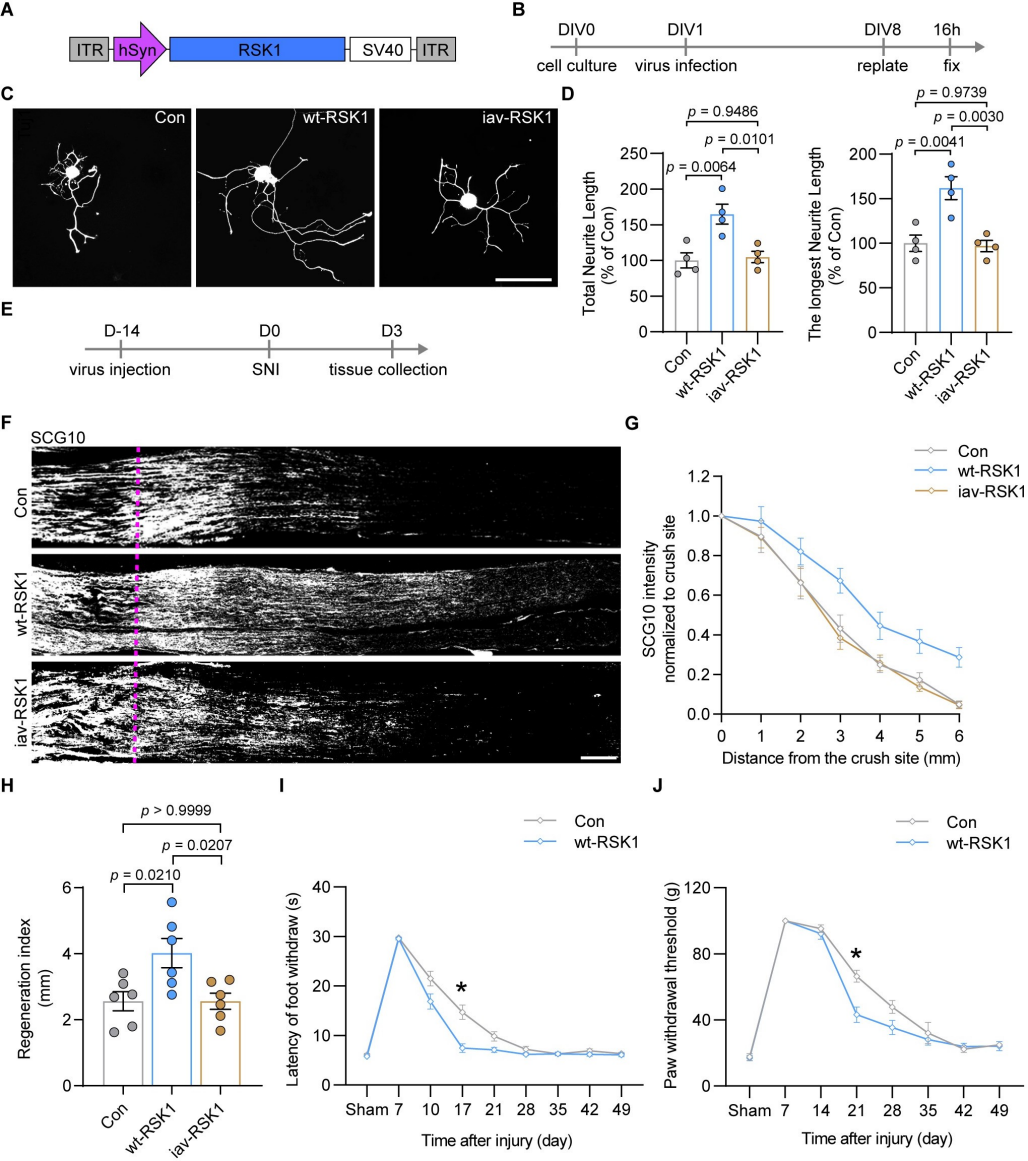
RSK1 promotes DRG neuron axon regeneration and functional recovery after injury. **(A)** A schematic representation of the AAV2/8-hSyn-RSK1 vector utilized in RSK1 OE experiments. **(B)** Timeline for RSK1 OE in DRG neurons at DIV1. **(C)** Representative images of cultured DRG neurons infected with control AAV2/8 (Con), AAV expressing wt-RSK1, or AAV expressing inactive mutant (S221A, S380A, and T573A) RSK1 (iav-RSK1). Scale bar, 100 μm. **(D)** Quantification of the total and the longest neurite outgrowth per neuron relating to (C) (mean ± SEM, 1-way ANOVA, Tukey post hoc test, *n* = 4 biologically independent experiments, approximately 50 cells/experiment on average). **(E)** Timeline for RSK1 OE in vivo and SNI. **(F)** Representative longitudinal sections from injured sciatic nerves. The crush site is indicated by a purple dotted line. Scale bar, 500 μm. **(G)** Normalized SCG10 intensity plotted in function of the distance from the crush line (*n* = 6 rats per group). **(H)** Axon regeneration in injured rats was quantified by regeneration indices obtained from SCG10 immunostaining on day 3 after injury (mean ± SEM, 1-way ANOVA, Tukey post hoc test, *n* = 6 rats per group). **(I, J)** Assessment of the recovery of thermal (I) or mechanical (J) sensory function after SNI in rats infected with AAV-wt-RSK1 or control AAV2/8 (mean ± SEM, 2-way ANOVA, Bonferroni post hoc test, *n* = 8 rats per group). The data underlying all the graphs shown in the figure are included in S1 Data. DIV1, 1 day in vitro; DRG, dorsal root ganglion; iav-RSK1, inactive RSK1; OE, overexpression; RSK1, ribosomal S6 kinase 1; SEM, standard error of the mean; SNI, sciatic nerve injury; wt-RSK1, wild-type RSK1.

We next asked whether manipulating the expression of RSK1 in neurons could facilitate their axonal regenerative potential. Rat wild-type RSK1 (wt-RSK1) was overexpressed via AAV2/8 under the control of the human synapsin (hSyn) promoter (AAV-wt-RSK1) (Fig 4A and 4B, S7A and S7B Fig). The in vitro neurite regrowth assay revealed that the overexpression of wt-RSK1 enhanced axon regrowth in primary DRG neurons (Fig 4C and 4D). To investigate whether the phosphorylated form is essential for RSK1 to be effective on axon regeneration, we mutated 3 phosphorylation sites (S221A, S380A, and T573A) of RSK1. The western blotting assay showed that wt-RSK1 significantly increased the level of p-eEF2K, while the mutant RSK1 had no obvious effect on phosphorylation of eEF2K (S7C and S7D Fig), suggesting the mutant RSK1 is phosphorylation inactive (named inactive RSK1 (iav-RSK1)). The in vitro neurite regrowth assay further revealed that the overexpression of iav-RSK1 had no significant effect on axon regrowth in DRG neurons (Fig 4C and 4D). When wt-RSK1 was overexpressed in DRG neurons in vivo (Fig 4E, S7E Fig), we observed significant increase of nuclear p-RSK^S221^ in DRG neurons (S7F and S7G Fig), along with enhanced p-eEF2K (S7H and S7I Fig). Anatomically, we found that the extension of SCG10^+^ axons after SNI was substantially increased compared with that in animals injected with control virus (Fig 4F and 4G). Meanwhile, the regeneration index was significantly higher in nerves treated with AAV-wt-RSK1 than in those treated with the control virus (Fig 4H). In contrast, iav-RSK1 had no significant effects on p-eEF2K expression (S7H and S7I Fig), and axon regeneration in vivo (Fig 4F–4H), indicating its phosphorylated form is essential for RSK1 to be effective on axon regeneration.

Regenerating axons of sciatic nerves extend to the epidermis and start to reinnervate the skin of the hind paw approximately 2 to 3 weeks after crush injury [40]. To assess functional recovery following axon regeneration, we first performed a behavioral test in rats with or without RSK1 overexpression following SNI to quantify the latency of heat-evoked hind paw withdrawal [41]. Neither group of animals showed any response to a radiant thermal stimulus at day 7 post-SNI (Fig 4I). Starting from day 10 post-SNI, the speed of recovery of the withdrawal latency was higher in the wt-RSK1 group than in the control group, reaching significance on day 17 post-SNI (Fig 4I). The Von Frey test was subsequently used to examine the mechanical sensory function. Animals from both the control and wt-RSK1 groups exhibited similar Von Frey scores at days 7 and 14 post-SNI (Fig 4J); however, after day 14, mechanical sensory function was better in the wt-RSK1 group than in the control group, as reflected by a significant difference at day 21 post-SNI (Fig 4J). Furthermore, the lines of the 2 groups in the thermal or mechanical sensory behavioral test converged and tended to be relatively stable starting at 35 days or 42 days postinjury, respectively (Fig 4I and 4J). Collectively, these data suggested that RSK1 facilitates axonal regeneration and functional recovery in DRG neurons.

### RSK1 promotes axon regeneration by regulating protein synthesis through eEF2

**Fig 5:**
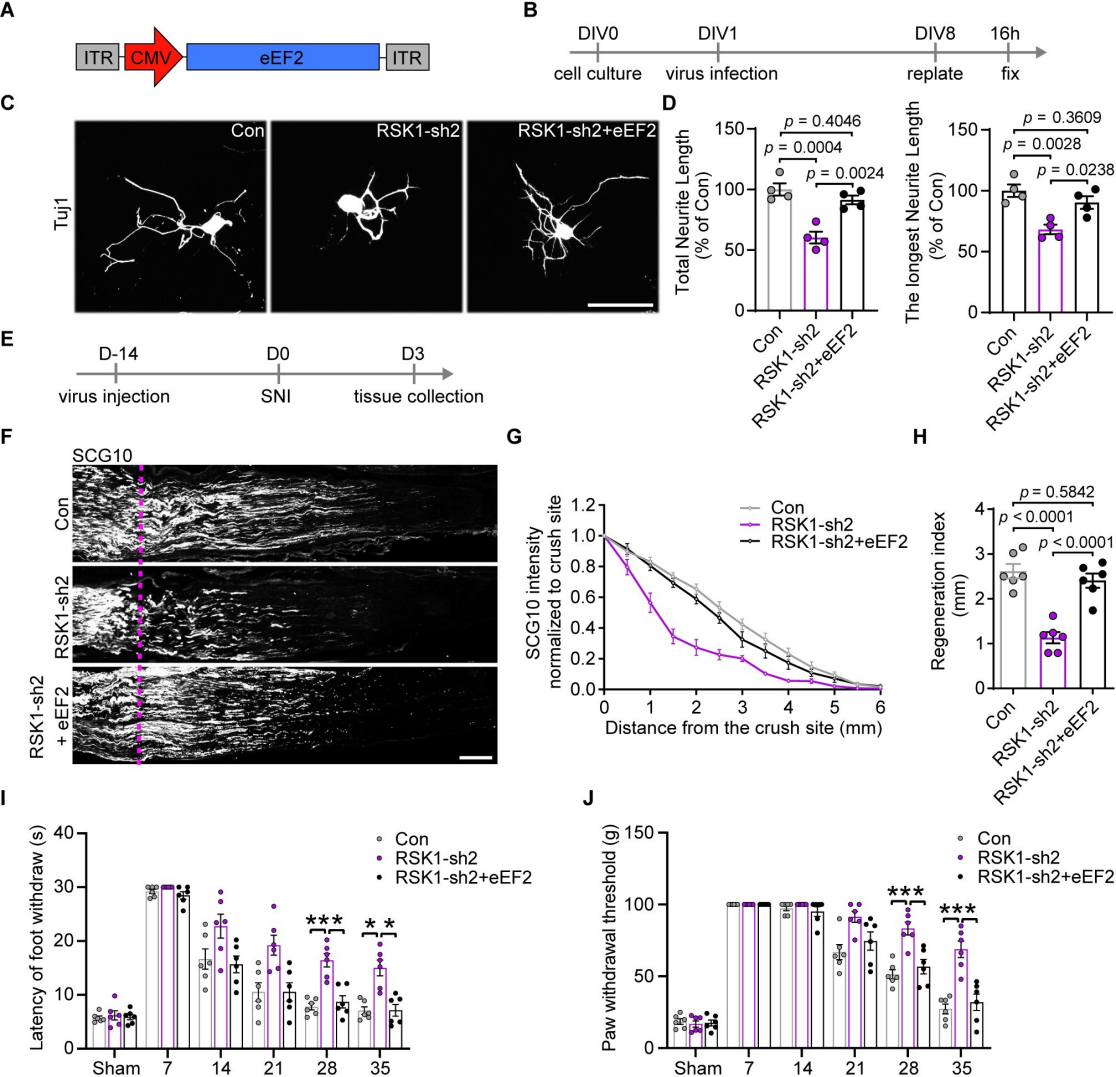
Overexpression of eEF2 rescues the inhibitory effect of RSK1 KD. **(A)** A schematic representation of the AAV2/8-CMV-eEF2 (eEF2) vector utilized in eEF2 overexpression experiments. **(B)** Timeline for RSK1 KD and/or eEF2 overexpression in DRG neurons at DIV1. **(C)** Representative images of cultured DRG neurons infected with control AAV (Con), AAV to knock down RSK1 (RSK1-sh2), or AAVs to knock down RSK1 and overexpress eEF2 (RSK1-sh2+eEF2). Scale bar, 100 μm. **(D)** Quantification of the total and the longest neurite outgrowth per neuron relating to (C) (mean ± SEM, 1-way ANOVA, Tukey post hoc test, *n* = 4 biologically independent experiments, approximately 50 cells/experiment on average). **(E)** Timeline for AAV infection in vivo and SNI. **(F)** Representative longitudinal sections from injured sciatic nerves. The crush site is indicated by a purple dotted line. Scale bar, 500 μm. **(G)** Normalized SCG10 intensity plotted in function of the distance from the crush line (*n* = 6 rats per group). **(H)** Axon regeneration in injured rats was quantified by regeneration indices obtained from SCG10 immunostaining on day 3 after crush injury (mean ± SEM, 1-way ANOVA, Tukey post hoc test, *n* = 6 rats per group). **(I, J)** Assessment of the recovery of thermal (I) or mechanical (J) sensory function after SNI in rats infected with RSK1-sh2, RSK1-sh2+eEF2, or control AAV2/8 (mean ± SEM, 2-way ANOVA, Bonferroni post hoc test, *n* = 6 rats per group). The data underlying all the graphs shown in the figure are included in S1 Data. DIV1, 1 day in vitro; KD, knockdown; RSK1, ribosomal S6 kinase 1; SEM, standard error of the mean; SNI, sciatic nerve injury.

Activated RSK1 is reported to dephosphorylate and activate translational elongation factor eEF2 through phosphorylating and inactivating eEF2K, resulting in subsequent induction of protein synthesis [42]. Our results have shown that the levels of p-RSK and p-eEF2K were significantly increased in DRG post-SNI (Fig 2, S2 and S3 Figs). Consistent with this, overexpression of RSK1 induced phosphorylation of eEF2K (S7C, S7D, S7H, and S7I Fig), leading to a down-regulation of p-eEF2 (the inactive form of eEF2) in DRG neurons (S8A and S8B Fig). Moreover, eEF2 is a major regulator of protein synthesis in neurons [43] and a regulator of axon outgrowth of DRG neurons [44,45]. These observations suggested that RSK1 might promote axon regeneration by regulating protein synthesis through RSK1-eEF2 axis post-SNI, leading us to assess the role of eEF2 by asking whether overexpressing eEF2 will rescue the effect of knocking down RSK1 (Fig 5A and 5B). The overexpression of eEF2 in cultured DRG neurons was confirmed by RT-qPCR and western blotting assays (S8C–S8E Fig). The in vitro axon regrowth assay showed that overexpression of eEF2 rescued the inhibitory effect of RSK1-KD on the total and longest neurite length of DRG neurons (Fig 5C and 5D). To determine the potential role of the RSK1-eEF2 axis in vivo, we intrathecally injected AAV2/8-RSK1-sh2 and AAV2/8-eEF2 (RSK1-sh2+eEF2) in the same animal (Fig 5E). The immunostaining assay showed that eEF2 was successfully overexpressed in DRG neurons in vivo (S8F and S8G Fig). Western blotting assay further confirmed this observation, along with RSK1 down-regulation (S8H–S8J Fig). Additionally, we observed that rats in the RSK1-sh2+eEF2 group showed more robust sciatic nerve regenerative ability compared with that of the RSK1-sh2 group. Although the maximal distance showed no differences, it was clear that the density of SCG10^+^ axons was higher in RSK1-sh2 +eEF2 group than that in the RSK1-sh2 group at the proximal end (Fig 5F–5H). To further assess the rescue effect of eEF2 on the behavior of RSK1 KD rats, we performed the thermal and mechanical sensory tests described above. In the heat plate and Von Frey tests, no significant differences were seen between either the RSK1-sh2 and control groups or the RSK1-sh2 and RSK1-sh2+eEF2 groups within 3 weeks postinjury. At 28 and 35 days post-SNI, animals in the RSK1-sh2 group showed less recovery of thermal and mechanical sensory function compared with that of animals in the control group, while animals in the RSK1-sh2+eEF2 group exhibited significantly better functional recovery than that of rats in the RSK1-sh2 group (Fig 5I and 5J). Furthermore, in the behavioral tests, the functional recovery score of rats in the RSK1-sh2+eEF2 group was comparable to that of control animals (Fig 5I and 5J). Together, these data indicated that RSK1 promotes axonal regeneration and functional recovery, at least partially, through activated eEF2.

### RSK1 is vital for regeneration-related protein synthesis

**Fig 6:**
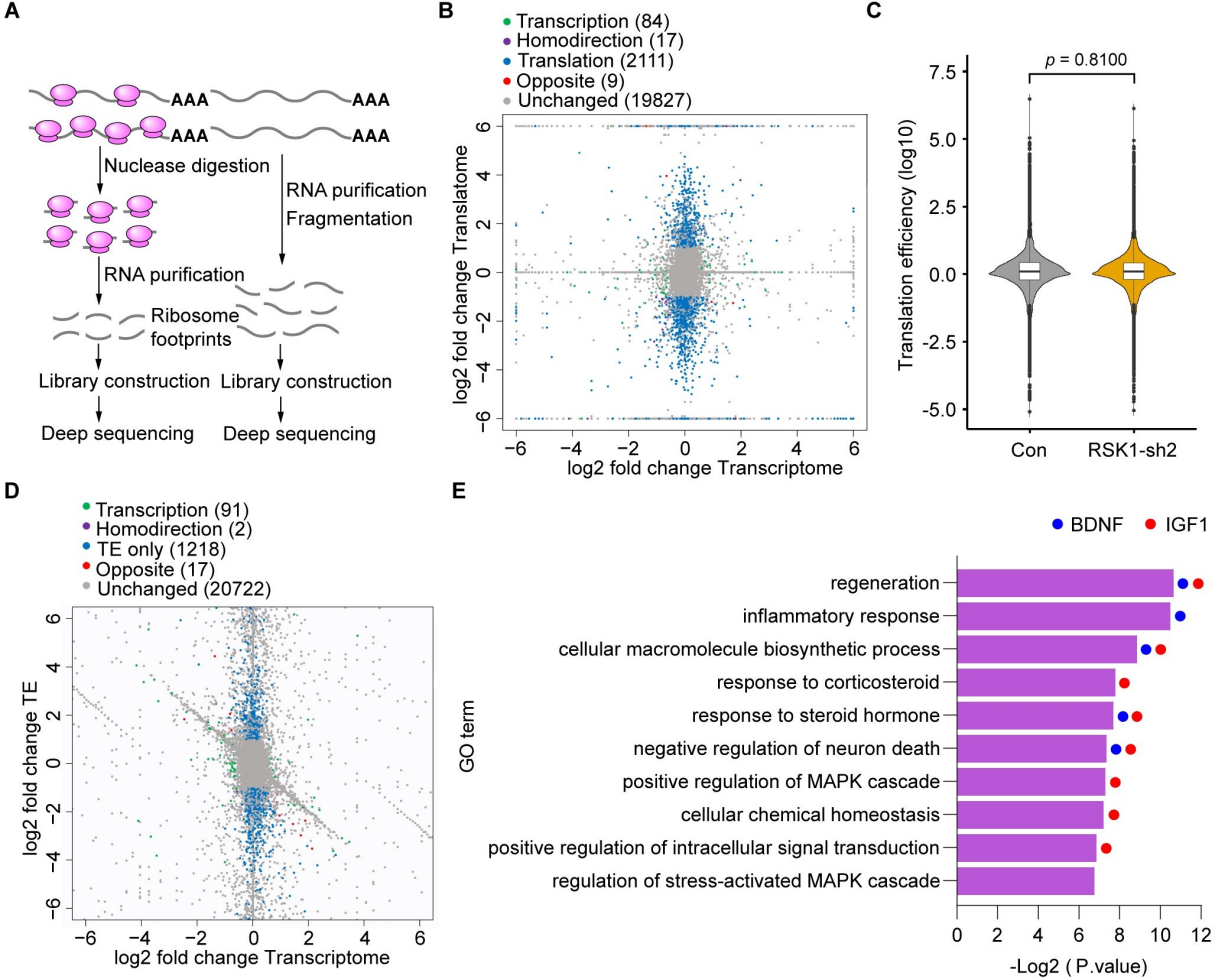
KD of RSK1 impairs the synthesis of regeneration-related proteins. **(A)** Experimental workflow of Ribo-seq. **(B)** Scatter plot of differentially expressed translatome and transcriptome between neurons infected with AAV2/8-RSK1-sh2 (RSK1 KD) or control shRNA AAV. **(C)** TE of mRNAs in neurons infected with RSK1 KD or control shRNA AAV. Box plots show mean and standard deviation within each group; violin plot shows the gene density at each y-axis value; *p*-value was calculated using the 2-sided Wilcoxon rank-sum test. **(D)** Scatter plot of genes with significant differential TEs between neurons with and without RSK1 KD. The TEs of the transcripts were calculated as the ratio of reads of RPFs to the total mRNA abundance. **(E)** Intersection of the results from GO analysis using DAVID and GO Resource of the differentially expressed genes with down-regulated TEs in the TE only and Opposite groups from (D) to determine which signaling/effector pathways are enriched with RSK1 KD. BDNF and IGF1 are indicated in each GO term in which they are involved by blue and red dots, respectively. The data underlying the graphs shown in Fig 6C and 6E are included in S1 Data. The data underlying the graphs shown in Fig 6B and 6D are included in the S1 and S2 Tables respectively. GO, Gene Ontology; KD, knockdown; RPF, ribosome-protected fragment; RSK1, ribosomal S6 kinase 1; TE, translation efficiency.

To determine the exact role of the RSK1-eEF2 axis in mRNA translation in DRG neurons, we next examined the mRNA translation in primary DRG neurons infected with AAV2/8-RSK1-sh2 or control AAV using ribosome profiling (Ribo-seq) [46], which combines ribosome footprints with deep sequencing. First, we found 79.33 ± 3.87% of neurons, along with 7.61 ± 1.37% of nonneuronal cells were successfully infected by AAV (S9A and S9B Fig), suggesting a preferential target of DRG neurons with relatively high efficiency. Following polysome isolation, the sample is treated with ribonuclease to digest unprotected RNA. The resulting ribosome-protected RNA fragments (or ribosome footprints) are used to generate a sequencing library (Fig 6A). We analyzed differential gene expression between primary DRG neurons with and without RSK1 KD and calculated log fold-changes between ribosome-bound RNAs (Translatome) and total transcripts (Transcriptome). The RNA-seq and RT-qPCR further confirmed that RSK1 was significantly inhibited in this experiment (S9C and S9D Fig). The Ribo-seq indicated that a total of 2,111 genes were regulated by RSK1 exclusively at the translational level (translation group), while 84 genes were regulated only at the transcriptional level (transcription group). Additionally, 9 genes were regulated via translational antagonism (Opposite group) (where genes exhibited increased mRNA levels but lower translational levels or vice versa). The number of genes in translation group (blue dots) is approximately 25 times that in transcription group (green dots) (Fig 6B, S1 Table). These data suggested that RSK1 preferentially serves as a translational, rather than transcriptional, regulator of target genes in DRG neurons.

Translational regulation can occur through differential translation efficiencies (TEs) of transcripts, which are calculated as the ratio of ribosome-bound RNA (ribosome-protected fragments [RPFs]) reads to total mRNA abundance to describe the propensity of mRNA to undergo translation. Our results have demonstrated that RSK1 can activate translational elongation factor eEF2, leading us to speculate that RSK1 increases overall TE of all mRNAs. Unexpectedly, we observed that compared with the control group, RSK1 KD did not affect overall mRNA TE (Fig 6C); however, several transcripts displayed significantly different TE values between DRG neurons with or without RSK1 KD. A total of 1,218 genes were regulated by RSK1 exclusively at the TE level (TE only group), while 17 genes were regulated via TE antagonism (Opposite group) (where genes exhibited increased mRNA levels but lower TEs or vice versa) (Fig 6D, S2 Table). To explore the functions of the genes for which the TEs were specifically reduced by RSK1 KD while the transcriptional levels were unchanged or induced, we applied Gene Ontology (GO) analysis of genes with down-regulated TEs in the TE only and Opposite groups using 2 well-accepted GO analysis tools, DAVID and GO Resource [47–49]. Intersectional results from these 2 tools revealed that selected genes were enriched in “regeneration,” “cellular macromolecule biosynthetic process,” “response to corticosteroid,” “response to steroid hormone,” “negative regulation of neuron death,” and “positive regulation of MAPK cascade” (Fig 6E, S3 Table). These terms are closely related to axon regeneration process, suggesting that RSK1 might play a previously unidentified role in modulating regeneration-related protein synthesis during axonal regeneration in DRG neurons.

### RSK1 induces the synthesis of regeneration-related protein BDNF and IGF1

**Fig 7:**
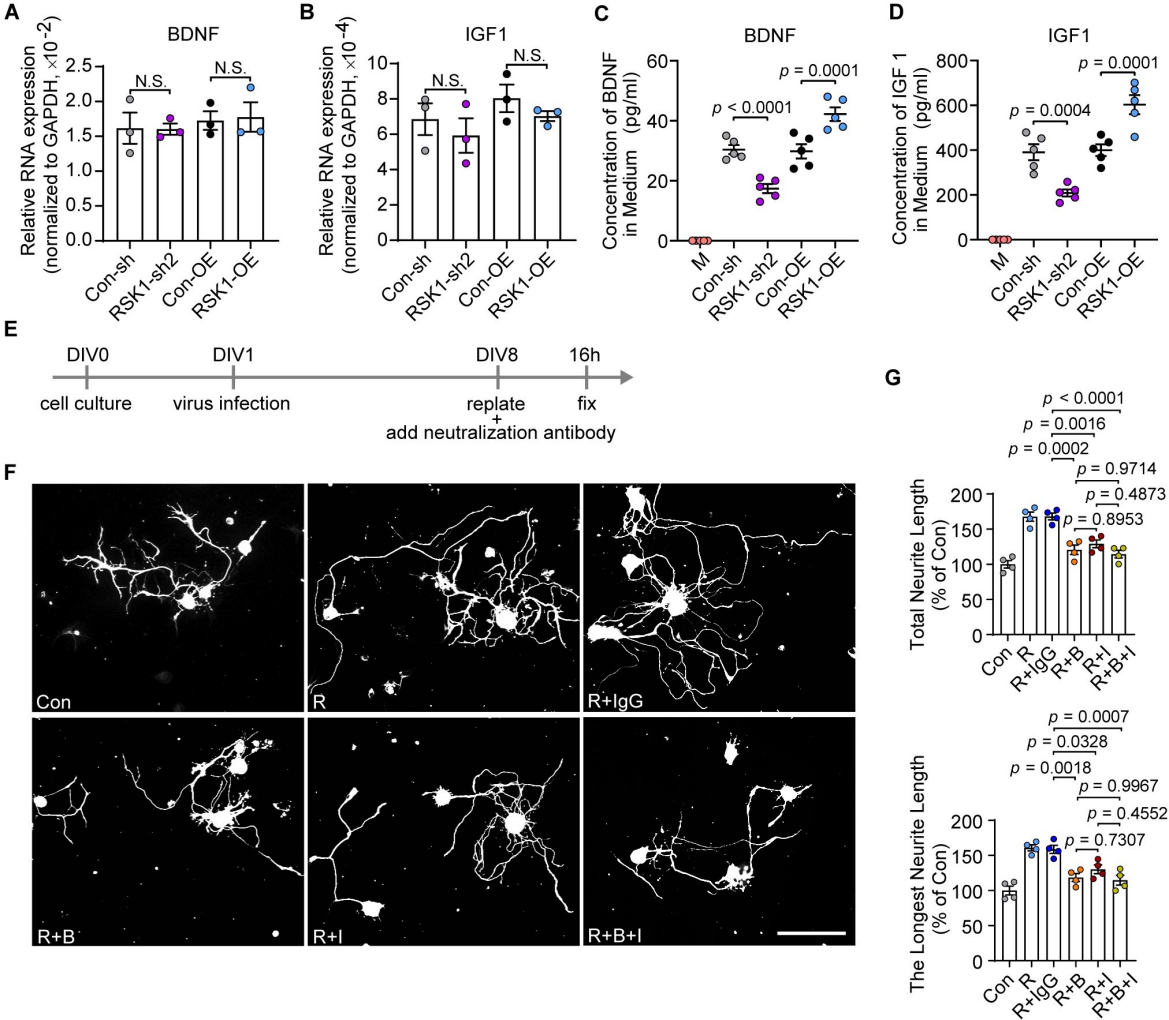
RSK1 promotes axon regeneration through BDNF and IGF1. **(A, B)** RT-qPCR analysis of the expression of BDNF (A) or IGF1 (B) in neurons infected with AAV expressing control shRNA (Con-sh), RSK1-shRNA2 (RSK1-sh2), RSK1 (RSK1-OE), or empty AAV (Con-OE) for 7 days (N.S., not significant, mean ± SEM, 1-way ANOVA, Bonferroni post hoc test, *n* = 3 biologically independent experiments). **(C, D)** Quantification of secreted BDNF (C) or IGF1 (D) levels in the supernatant of neurons without AAV infection (M) or neurons infected with AAV expressing Con-sh, RSK1-sh2, RSK1-OE, or Con-OE for 7 days followed by replating for 16 hours as determined by ELISA (mean ± SEM, 1-way ANOVA, Bonferroni post hoc test, *n* = 5 biologically independent samples). **(E)** Timeline for neutralization antibody incubation of DRG neurons infected with RSK1-OE or Con-OE followed by replating. After replating, the neurons infected with AAV-Con-OE were not incubated with any antibody (Con), and the neurons infected with AAV-RSK1-OE were incubated with no antibody (R), antibody IgG (R+IgG), BDNF neutralizing antibody (R+B), IGF1 neutralizing antibody (R+I), or both BDNF and IGF1 neutralizing antibodies (R+B+I). Antibody details can be found in S8 Table. **(F)** Representative images of DRG neurons treated as in (E). Scale bar, 100 μm. **(G)** Quantification of the total and the longest neurite outgrowth per neuron relating to (F) (mean ± SEM, 1-way ANOVA, Tukey post hoc test, *n* = 4 biologically independent experiments, approximately 50 cells/experiment on average). The data underlying all the graphs shown in the figure are included in S1 Data. DRG, dorsal root ganglion; ELISA, enzyme-linked immunosorbent assay; OE, overexpression; RSK1, ribosomal S6 kinase 1; RT-qPCR, reverse transcription quantitative real-time PCR; SEM, standard error of the mean.

In order to clarify the notion that RSK1 induces regeneration-related protein synthesis during axonal regeneration in DRG neurons, we focused our attention on the well-known regeneration-related genes with the highest hits among the top enriched GO terms. We found that the neurotrophic factors IGF1 and BDNF were observed in 8 and 5 of the top 10 enriched biological processes, respectively (Fig 6E, S4 Table), indicating that they are preferentially involved in the regeneration-related biological processes regulated by RSK1. Indeed, BDNF and IGF1 have been shown to be axonal regrowth inducers in the PNS [50,51] and thus were chosen as representative target molecules of RSK1. Through RT-qPCR, we validated that RSK1 did not change the transcriptional levels of either BDNF or IGF1 in primary DRG neurons treated with AAV-RSK1-sh2 or AAV-RSK1-OE (S10A and S10B Fig, Fig 7A and 7B). Given BDNF and IGF1 usually function via secreted form, an enzyme-linked immunosorbent assay (ELISA) was performed, which showed that BDNF and IGF1 were secreted by DRG neurons, and the levels of secreted BDNF and IGF1 in the supernatant were significantly altered when RSK1 was knocked down or overexpressed (Fig 7C and 7D). These results confirmed that instead of altering the transcriptional levels of BDNF and IGF1, RSK1 is essential for their translation. To further determine whether the RSK1-induced synthesis of BDNF or IGF1 affects DRG axon regrowth, RSK1-overexpressing neurons were treated with neutralizing antibodies against BDNF and/or IGF1 with confirmed neutralizing capability (S10C and S10D Fig) or nonspecific IgG (Fig 7E). The in vitro axon regrowth assay showed that inhibiting BDNF or IGF1 alone using neutralizing antibodies significantly blocked the RSK1 overexpression-mediated enhancement of axon regrowth. However, the combination of the 2 neutralizing antibodies showed no synergistic effect (Fig 7F and 7G).

The respective receptors for BDNF and IGF1 are differentially expressed in different subtypes of DRG neurons. For instance, TrkB is only expressed on a subset of DRGs, while IGF1R is more generally expressed in a variety of DRG subsets. To investigate whether overexpression of RSK1 is preferentially driving regeneration of TrkB^+^ DRG neurons, we performed fluorescence in situ hybridization (FISH; TrkB) combining with immunostaining (Tuj1) in cultured DRG neurons transfected with RSK1 or control vectors (S10E Fig), showing no preferential growth-promoting effect in TrkB^+^ neurons over TrkB^−^ ones either in the control or RSK1 overexpressed condition (S10F Fig). Together, these data indicated that regeneration-related BDNF and IGF1 enhance axon regrowth under the regulation of RSK1.

### RSK1 enhances axon regeneration in PTEN-deleted retinal ganglion neurons

Finally, we assessed whether RSK1 promotes axon regeneration in the adult CNS. Compared with other types of neurons in the CNS, retinal ganglion cells (RGCs) are easily accessible for viral manipulations through intravitreal injection. Additionally, with all axons emanating from RGCs forming the optic nerve, the optic nerve crush (ONC) injury becomes an important experimental model to investigate CNS axon regeneration and repair [9,20,52]. Here, we first employed the ONC model in adult rats. The IHC assay showed that, in contrast to that observed in DRG neurons following SNI (Fig 2), neither the expression nor the phosphorylation levels of RSK1 in RGCs were changed following ONC (S11 Fig), indicating that the activity of RSK1 is not up-regulated in RGCs after injury. To determine the role of RSK1 in axonal regeneration of RGCs, AAV2 expressing EGFP, wt-RSK1 or phospho-mimetic (S221D, S380D, and T573D) RSK1 was intravitreally injected. Fourteen days later, we observed that 76.19 ± 3.85% of RGCs were infected (S12A and S12B Fig). The immunostaining assay showed that the levels of RSK1 and p-eEF2K were significantly increased in the animals overexpressing wt-RSK1, as well as that of p-eEF2K in the animals overexpressing phospho-mimetic RSK1, indicating the phospho-mimetic RSK1 is active in RGCs (named active RSK1 (av-RSK1)) (S12C–S12F Fig). Next, we overexpressed wt-RSK1 or av-RSK1 in RGCs followed by ONC and axonal labeling (S12G Fig). Neither wt-RSK1 OE nor av-RSK1 OE led to any optic nerve regeneration (S12H and S12I Fig). These data indicated that RSK1 alone does not affect optic nerve regeneration in rats.

**Fig 8:**
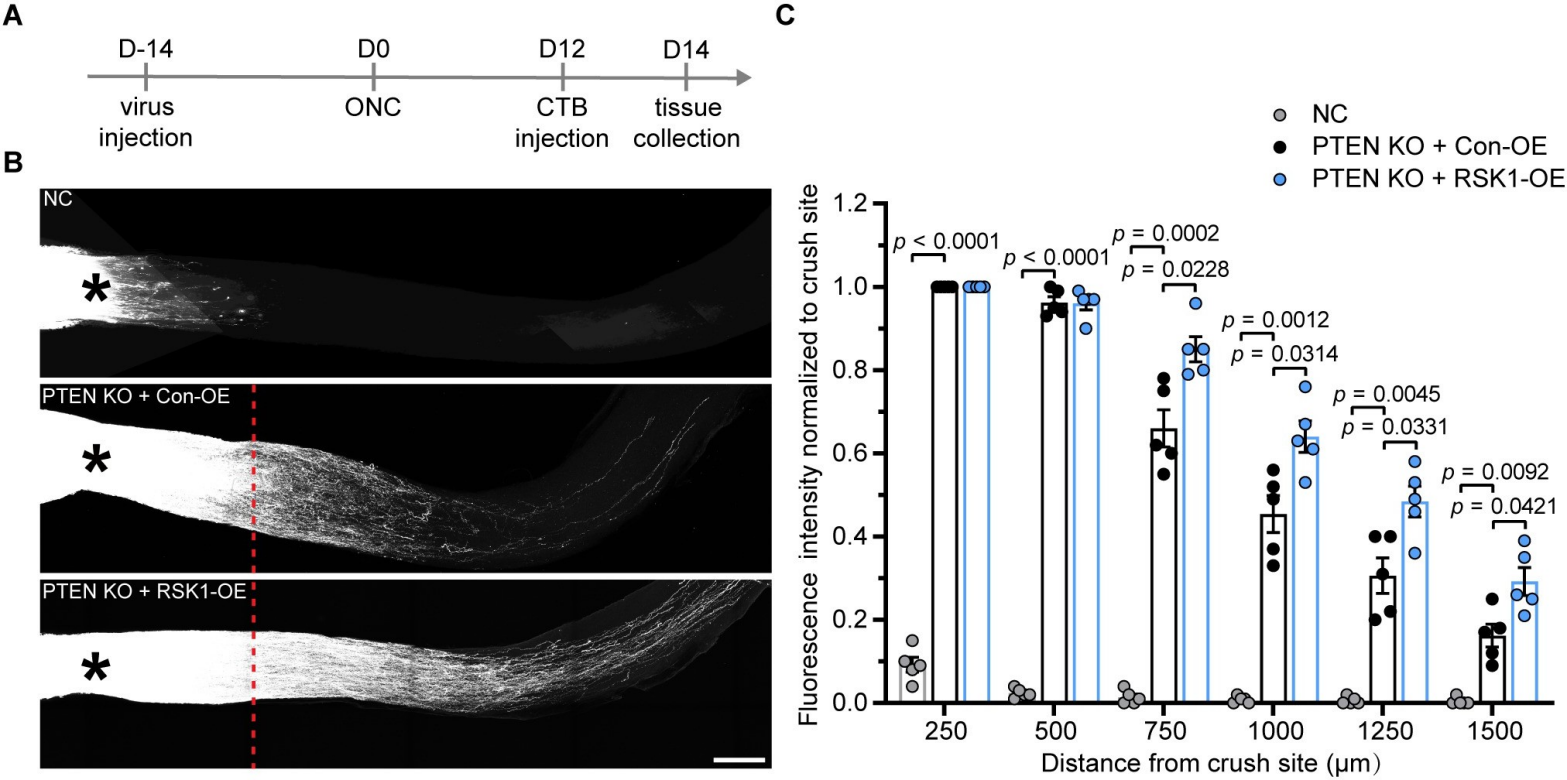
RSK1 enhances PTEN deletion-induced axon regeneration of retinal ganglion neurons in the adult mouse. **(A)** Timeline for AAV2 injection, ONC injury, and CTB injection. **(B)** Representative images of the cleared whole-mount optic nerves from Rosa26-Cas-9 mice 2 weeks postinjury. AAVs with EGFP only (NC), or AAVs with gRNA targeting PTEN (AAV-PTEN-gRNA) combined with AAV expressing RSK1 (RSK1-OE) or empty AAV (Con-OE) were intravitreally injected into Rosa26-Cas-9 mice. Axons were labeled by AF 555-conjugated CTB. The crush site is indicated by an asterisk, and the position where PTEN KO+RSK1-OE and PTEN KO+Con-OE conditions start to show differences is indicated by a red dotted line. Scale bar, 200 μm. **(C)** Normalized fluorescence intensity plotted in function of the distance from the crush line (mean ± SEM, 2-way ANOVA, Tukey post hoc test, *n* = 5 mice per group). The data underlying all the graphs shown in the figure are included in S1 Data. AF, Alexa Fluor; CTB, cholera toxin B subunit; gRNA, guide RNA; KO, knockout; ONC, optic nerve crush; PTEN, phosphatase and tensin homolog; RSK1, ribosomal S6 kinase 1; SEM, standard error of the mean.

As phosphatase and tensin homolog (PTEN) deletion is known to induce axon regeneration in RGCs of adult mice [20], in order to utilize the transgenic mice to knockout (KO) PTEN, we then shifted the animal model from rat to mouse to test whether RSK1 overexpression has a synergistic effect on PTEN deletion-induced optic nerve regeneration. Before the investigation in PTEN KO mice, we first overexpressed wt-RSK1 and av-RSK1 in wild-type mice (S13A–S13D Fig). Likewise, we observed no significant axon regeneration following ONC, indicating that RSK1 alone also does not affect optic nerve regeneration in mice (S13E and S13F Fig). Next, AAVs with guide RNA (gRNA) targeting PTEN were intravitreally injected into Rosa26-Cas-9 mice [53] (Fig 8A). The increased level of p-S6^S240/244^ confirmed that PTEN was silenced (S13G and S13H Fig). Consistent with previous studies [20], PTEN deletion significantly enhanced axon regeneration of RGCs (Fig 8B and 8C). In addition, we found that RSK1 OE significantly enhanced the effect of PTEN deletion on axon regeneration (Fig 8B and 8C). Together, these data suggested that RSK1 up-regulation enhances axonal regeneration in PTEN-deleted RGCs after injury in the adult CNS.

## Discussion

The results of our study demonstrated that RSK1 is up-regulated in DRG neurons after SNI. This, in turn, increases the translation of BDNF and IGF1, leading to enhanced axon regeneration in mature PNS neurons, along with the recovery of sensory function. Our study further suggested that RSK1 may also play a role in axonal regeneration in the adult CNS. To our knowledge, this is the first demonstration that RSK1 preferentially regulates the translation, instead of transcription, of regeneration-related proteins.

The current consensus is that, compared with the PNS, the CNS is characterized by a diminished intrinsic neuronal regenerative capacity and an extrinsic inhibitory environment, both of which pose major obstacles for axon regeneration. Recently, axon regeneration research focus has begun to shift from the extrinsic environment toward the intrinsic regenerative properties of neurons and their axons. Several intracellular mechanisms involved in the control of axon regeneration have been uncovered, including signaling pathways and transcription factors relating to regenerative programs, axonal transport and trafficking, cytoskeletal dynamics, and epigenetic modifications [1,54–56]. However, the differences in translational regulatory mechanisms involved in axon regeneration between the PNS and CNS remain poorly understood. In the present work, we identified RSK1 as a novel enhancer of axon regeneration in DRG neurons and showed that RSK1 can induce the translation of regeneration-related protein mRNA. In addition, our results showed that the expression and function of RSK1 in mature RGCs differ greatly from those in DRG neurons, which may partially explain the large difference in regenerative potential between neurons in the PNS and CNS.

De novo protein synthesis is known to be critical for axon regeneration in the adult mammalian PNS and CNS [57–59], in which mTOR plays a critical role. Molecules downstream of mTOR, such as S6K1, 4E-BP1, eEF2K, and EIF4B [60], have been reported to be regulated by another protein synthesis regulator, RSK; meanwhile, RSK can activate mTORC1 directly. These observations illustrate that there is complex crosstalk between RSK and mTOR pathways involved in protein synthesis. We found that inhibition of mTOR by rapamycin does not affect axon regrowth in mature DRG neurons in vitro, in agreement with that previously reported [22,23]. In contrast, inhibiting RSK1 has a negative effect on axon regrowth, suggesting that RSK1 regulates protein synthesis in an mTOR/S6K-independent manner, which has also been demonstrated in several other studies [30,61]. Although rapamycin does not affect DRG axon growth, mTOR nonetheless has been demonstrated to play a positive role in axon regeneration in DRG neurons in a rapamycin-resistant manner [39,62]. The mTOR-dependent and mTOR-independent roles of RSK together with mTOR pathway increase the complexity and stability of the protein synthesis regulatory network that might be beneficial for PNS axon regeneration after injury.

A previous study has shown that RSK1 is sufficient for differentiation of PC12 cells, as evidenced by neurite outgrowth [63]. However, its role in axon regeneration is unclear. The present study revealed that RSK1 is required for axon regeneration in DRG neurons, while RSK1 overexpression promotes axon regrowth and the recovery of sensory function, suggesting that RSK1 is a newly identified facilitator of axon regeneration in the PNS. Unlike RSK1, the expression of RSK2 in DRG was barely changed post-SNI, suggesting distinct injury response of RSK family members in the PNS. In addition, active RSK2 appeared without effect on neurite outgrowth of PC12 cells under the same conditions with RSK1 [63]. In the spinal motor neurons, RSK2 was reported to negatively regulate axon growth apparently via the feedback inhibition on the ERK pathway, which cannot be compensated by other members of the RSK family [64]. These observations suggest that individual RSK family members play different roles in axon growth. It will be extremely interesting to dissect their respective roles in peripheral and central axon regeneration in future.

Previous studies have shown that RSK can control protein synthesis during translation initiation or elongation by regulating S6 or eEF2K [29]. Here, we clarified that RSK1 enhances axon regeneration through translational elongation factor eEF2, at least partially. However, unexpectedly, we found that RSK1 does not have an obvious impact on overall TE of all mRNAs, which is in line with another study reporting that levels of global protein synthesis were unaffected when eEF2K, a bridge between RSK1 and eEF2[19], was knocked down [65]. Genes in which TE was inhibited by RSK1 KD were surprisingly enriched in regeneration-related biological processes. Besides the GO terms directly involved in axon regeneration, such as “regeneration,” “cellular macromolecule biosynthetic process,” and “negative regulation of neuron death,” the genes down-regulated in TE by RSK1 KD are enriched in “response to corticosteroid,” “response to steroid hormone,” and “positive regulation of MAPK cascade.”. It is reported that axon regeneration of DRG neurons was increased through corticosteroid response-dependent transcriptional programs [66]. Steroid hormone was demonstrated to act at the level of RNA and protein synthesis to accelerate regeneration of the hypoglossal nerve after injury [67]. Several studies have suggested that activation and retrograde transport of MAPKs play an important role in axon regeneration [68,69]. These observations, together with our results, suggest RSK1 enhances axon regeneration by regulating the translation of genes involved in regeneration-related biological processes. To demonstrate this notion, we chose IGF1 and BDNF for further investigation, which were preferentially involved in the top enriched regeneration-related GO terms regulated by RSK1 and were reported to be vital enhancers of axon regeneration [50,51]. We demonstrated that RSK1 enhances axon regrowth in DRG neurons by increasing the translation, rather than transcription, of BDNF and IGF1. However, the location of RSK1 regulating mRNA translation remains unclear. Although studies on the role of protein synthesis have mainly focused on the site of local injury in the axon [11,12,70], a recent study revealed that the somatic response to injury involves the extensive regulation of protein synthesis [13]. Thus, further studies are needed to address whether RSK1 affects mRNA translation within local axons or in DRG neuronal somata.

Defining how injured mature PNS neurons switch to a proregenerative state may not only reveal the basic biology of mature mammalian neurons, but may also suggest novel therapeutic strategies for promoting axonal regeneration within both the PNS and CNS. Hence, we then tested the role of RSK1 in the CNS. First, we found that neither the expression nor the activity of RSK1 was significantly changed, which might partially account for the weak intrinsic axon regenerative capacity in neurons of the CNS. Furthermore, different from when it was combined with PTEN deletion, RSK1 OE alone could not promote axon regeneration in RGCs, suggesting that the proteins positively regulated by RSK1 are insufficient to promote axon regeneration in RGCs. A possible explanation is that injured adult RGCs are not so sensitive to growth factors as they do during the development [71–74]. In line with these results, exogenous expression of BDNF or IGF1 alone exerts no significant impact on optic nerve regeneration. In contrast, they became effective when combined with other factors (e.g., OPN or lin28) that are able to enhance the mTOR activity of adult RGCs [72,73]. PTEN deletion has emerged as one of the most powerful strategies for axon regeneration in RGCs presumably through activating the PI3K-mTOR pathway. This pathway is the central effector of multiple growth factors’ signaling to promote protein synthesis and cell growth, leading injured RGCs to a regrowth state [75,76]. Therefore, PTEN deletion is likely to enable RGCs to become more responsive to the regeneration-related proteins up-regulated by RSK1, including the BDNF and IGF1.

Taken together, our data revealed that RSK1 was differentially activated after nerve injury in DRG neurons and RGCs, which may partially account for the large differences in regenerative responses between the PNS and CNS. Unlike previous work mainly focusing on regulators of transcription or posttranscription of regeneration-related genes, we determined RSK1 as a modulator of protein synthesis that is essential for axon regeneration. Our results highlight the importance of a protein synthesis regulator in enhancing axonal regeneration in the adult mammalian PNS and provide a novel strategy that can be combined with current avenues in promoting axon regeneration in the CNS.

## Materials and methods

### Animal surgery and tissue preparation

All experimental procedures involving animals were performed in compliance with the Institutional Animal Care guidelines of Nantong University and were approved by the Ethics Committees of Nantong University (Approval ID: S20200323-151) and the Administration Committee of Experimental Animals, Jiangsu Province, China (Approval ID: SYXK [SU] 2017–0046). All the animals used in the experiments were maintained in a pathogen-free facility at 23 to 24°C under a 12-hour light, 12-hour dark regimen with free access to food and water.

Adult Sprague–Dawley (SD) rats (approximately 200 g) underwent surgery for sciatic nerve crush injury, as previously described with some modification [77]. Briefly, the rats were anesthetized by an intraperitoneal injection of 40 mg/kg sodium pentobarbital, and the sciatic nerve was exposed by a small incision. The left sciatic nerve at 10 mm above the bifurcation into the tibial and common fibular nerves was crushed 3 times (10 seconds each time) with a pair of forceps at a force of 54 N, and the crush site was marked with a 9–0 nylon suture as previously reported [78]. The L4-5 DRGs were collected at days 0, 1, and 4 after surgery. For the conditioning injury, AAV intrathecal injection and sciatic nerve transection or sham injury were performed simultaneously. Fourteen days later, a crush injury (the second injury) was performed approximately 8 mm proximal to the first injury site and sciatic nerve regrowth between the 2 injury sites was analyzed after another 2 days. Male and female rats were randomly distributed among the treatment groups for all experiments. No gender-specific differences were observed in any of the analyses.

### AAV constructs and packaging

The sequences of the shRNAs targeting rat RSK1 are shown in S5 Table. The AAV serotype 2/8 constructs for knocking down rat RSK1, or overexpressing rat mutant RSK1 were packaged by BrainVTA (Wuhan, China). The AAV serotype 2/8 construct for overexpressing rat wt-RSK1 and the AAV serotype 2/2 constructs for overexpressing mouse RSK1, or PTEN-targeting gRNA1-5 were packaged by Vigenebio (Jinan, China). The AAV serotype 2/8 construct for overexpressing rat eEF2 and the AAV serotype 2/2 constructs for overexpressing mouse mutant RSK1 were packaged by OBIO Technology (Shanghai, China). Virus titer (approximately 5 × 10^12^ genome copies (GC)/mL) was measured by RT-qPCR.

### Intrathecal injection

For intrathecal injection of AAV viruses, adult rats were anesthetized and shaved to expose the skin around the lumbar region. A total of 10 μL of virus solution was injected into the cerebrospinal fluid between vertebrae L5 and L6 using a 10-μL Hamilton syringe. After injection, the needle was left in place for an additional 2 minutes to allow the fluid to diffuse. Rats were left to recover for 2 weeks to ensure substantial viral expression before behavioral or surgical procedures.

### RNA extraction and RT-qPCR analysis

Total RNA was extracted from lumbar (L)4/L5 DRG tissue or primary DRG neurons using TRIzol regent (Invitrogen, Carlsbad, California, United States of America) and then treated with amplification-grade DNase Ι (Thermo Fisher Scientific, Waltham, Massachusetts, USA) according to the manufacturer’s instructions. Total RNA from each sample was quantified using a Nanodrop 1000 spectrophotometer (NanoDrop Technologies, Wilmington, Delaware, USA). The purified total RNA was converted into cDNA using the PrimeScript RT Reagent Kit (TaKaRa Biotechnology, Dalian, China) according to the manufacturer’s protocol. For qPCR, 5 ng of cDNA was amplified in a 10-μL reaction containing SYBR Premix Ex Taq (TaKaRa Biotechnology) using a 2-step procedure. Melt curve analysis was enabled at the end of amplification. All samples were normalized against GAPDH and quantified using the 2^−ΔΔCT^ method. The experiment was repeated in triplicate. The primers used are listed in S6 Table.

### Ribosomal profiling

To immobilize initiating ribosomes, harringtonine was diluted in cell culture medium to a final concentration of 2 μg/mL. Cells were incubated for 120 seconds with harringtonine in an incubator. Next, to block translational elongation, cycloheximide was added to the cell culture medium to a final concentration of 100 μg/mL. Cells were mixed well and immediately lysed. The extracts, resuspended in lysis buffer, were transferred to new microtubes, pipetted several times, and incubated on ice for 10 minutes. The cells were then triturated 10 times through a 26-G needle. The lysate was centrifuged at 20,000 × *g* for 10 minutes at 4°C, and the supernatant was collected. To prepare RPFs, 7.5 mL of RNaseI and 5 mL of DNase I (both NEB; Ipswich, Massachusetts, USA) were added to 300 mL of lysate and incubated for 45 minutes at room temperature with gentle mixing on a Nutator mixer. Nuclease digestion was stopped by adding 10 mL of SUPERase RNase inhibitor (Ambion, Austin, Texas, USA). Size-exclusion columns (illustra MicroSpin S-400 HR Columns; GE Healthcare, Pittsburgh, Pennsylvania, USA; catalog no. 27-5140-01) were equilibrated with 3 mL of polysome buffer by gravity flow and centrifuged at 600 × *g* for 4 minutes at room temperature. Then, 100 mL of digested RPFs was added to the column and centrifuged at 600 × *g* for 2 minutes. Next, 10 mL of 10% (*w/v*) SDS was added to the elution, and RPFs with a size greater than 17 nucleotides were isolated using the RNA Clean and Concentrator-25 Kit (*Zymo Research*, Orange, California, USA; R1017) according to the manufacturer’s instructions. rRNA was removed using a previously reported method [79]. Briefly, short (50 to 80 bases) antisense DNA probes complementary to rRNA sequences were added to a solution containing RPFs, and RNase H (NEB) and DNase I (NEB) were added to digest the rRNA and residual DNA probes, respectively. Finally, RPFs were further purified using magnet beads (Vazyme, Nanjing, Jiangsu, China). Ribosomal profiling libraries were constructed using NEB Next Multiple Small RNA Library Prep Set for Illumina (catalog nos E7300S and E7300L). Briefly, adapters were added to both ends of the RPFs, followed by reverse transcription and PCR amplification. The 140- to 160-bp size PCR products were enriched to generate a cDNA library and sequenced by Gene Denovo Biotechnology in the Illumina HiSeq X10 platform.

### RNA sequencing

Total RNA was extracted using TRIzol reagent (Invitrogen) according to the manufacturer’s protocol. RNA quality was assessed on an Agilent 2100 Bioanalyzer (Agilent Technologies, Palo Alto, California, USA) and checked using RNase-free agarose gel electrophoresis. After total RNA was extracted, the mRNA was enriched by Oligo(dT) beads. The enriched mRNA was then fragmented into short fragments using fragmentation buffer and reverse transcribed into cDNA using random primers. Second-strand cDNA was synthesized using DNA polymerase I, RNase H, dNTPs, and buffer. The cDNA fragments were purified with a QIAquick PCR Extraction Kit (QIAGEN, Venlo, the Netherlands), end-repaired, polyadenylated, and ligated to Illumina sequencing adapters. The ligation products were size-selected by agarose gel electrophoresis, PCR amplified, and sequenced using the Illumina HiSeq2500 platform by Gene Denovo Biotechnology (Guangzhou, China). Ribo-seq and RNA-seq data have been deposited in SRA under accession number SRP317959.

### Primary DRG neuron culture

DRGs from adult (8-week-old) rats were dissected in cold HBSS and digested with 0.5 mg/mL collagenase (Roche Diagnostics, Basel, Switzerland) for 2 hours at 37°C, followed by digestion with 0.125% trypsin for 30 minutes at 37°C. Tissues were triturated in culture medium (Neurobasal medium with 2% B27, 1% glutamine; Thermo Fisher Scientific) with 1-mL pipette tips and passed through a 70-μm cell strainer. The cells were resuspended in culture medium and plated in 24-well plates precoated with poly-L-lysine. For replating DRG neuron culture, at DIV3 of primary DRG culture, cells were gently pipetted onto culture dishes. Cells were flushed by 20 to 30 rounds of pipetting in each well of a 6-well plate. After resuspending, the cells were replated onto a 24-well plate. For the small-molecule inhibitor (MedChemExpress, Monmouth Junction, New Jersey, USA), recombinant BDNF (PeproTech, Suzhou, China), IGF1 (Abcam, Cambridge, Massachusetts, USA), or neutralizing antibody treatment, the molecules were added immediately after replating. For in vitro AAV infection, virus was added at DIV1, and the cells were replated 7 days later. Fixation and staining were performed 16 hours after replating. Tuj1 staining was used to visualize neuronal axons and cell bodies. The longest and total lengths of neurites from each DRG neuron were measured by NeuronJ in ImageJ. In each experiment, approximately 50 neurons per condition were selected randomly, and the length of each neurite was measured manually. The longest and total axon length was quantified from at least 3 independent experiments.

### CCK-8 cell viability assay

A Cell Counting Kit-8 (Dojido, Kumamoto, Japan) was used to assess cell survival according to the manufacturer’s manual. Briefly, 10 μL of CCK-8 solution was added to 100 μL of medium solution in each neuronal culture well of a 96-well plate and incubated for 2 hours at 37°C. The absorbance at 450 nm was measured with a BioTek Synergy 2 Plate Reader.

### In situ hybridization

Digoxigenin (DIG)-labeled probes for rat RSK1, RSK2, RSK3, and RSK4 were synthesized using the DIG RNA Labeling Kit (Roche Diagnostics). After treatment with proteinase K, the DRG sections were prehybridized for 2 hours and then hybridized with DIG-labeled probes overnight at 37°C in a humidified chamber. The sections were then blocked with AKP-conjugated Fab anti-DIG antibody (Roche Diagnostics) overnight at 4°C and stained by 5-bromo-4-chloro-3-indolyl phosphate and nitroblue tetrazolium (BCIP-NBT; Roche Diagnostics) for visualization and imaging. Detailed probe information is provided in S7 Table.

### Immunocytochemical and immunohistochemical procedures

Primary cultured DRG neurons were fixed for 15 minutes in 4% paraformaldehyde (PFA), blocked with 5% normal horse serum in PBS/0.3% Triton X-100 for 1 hour and incubated overnight at 4°C with a primary antibody against Tuj1 in 2% BSA. For IHC on tissue sections, rats were transcardially perfused with 100 mL of PBS followed by 100 mL of 4% PFA. The L4/L5 DRG tissues or the sciatic nerves were removed, post-fixed in the same fixative overnight at 4°C, and cryoprotected in 30% sucrose, also overnight. Cryostat sections (20-μm thick) were cut and processed for IHC. After incubation with a blocking buffer, the sections were incubated with the primary antibody at 4°C overnight and then with Alexa Fluor-conjugated secondary antibody. Detailed antibody information is provided in S8 Table. As for the specificity of these antibodies against RSK1 versus RSK2 phosphorylation, without definitive evidence, e.g., knocking out RSK1, it is difficult to rule out reacting with RSK2. Images were obtained with a Zeiss Axio Imager M2 microscope. Exposure time and gain were maintained constant between conditions for each fluorescence channel. For quantitative analysis, SCG10 fluorescence intensity was measured along the length of the sciatic nerve using ImageJ. A regeneration index was calculated by measuring the distance from the crush site in which the average SCG10 fluorescence intensity was half that observed at the crush site [38]. For quantitative analysis of fluorescence intensity (RSK1, pRSK1^S380^, pRSK1^S221^, or pRSK1^T573^), the nucleus and soma of DRG or RGC neurons were manually outlined in images. To minimize variability between images, the intensity values of each cell were normalized to the background fluorescence signal, and mean values of intensities were calculated for each animal or sample using ImageJ. All measurements were performed blind to the experimental groups.

### FISH in combination with immunocytochemistry

FISH was performed with a FISH Kit (RiboBio, Guangzhou, China). In brief, primary cultured rat DRG neurons on coverslips were briefly rinsed in PBS and fixed with 4% PFA at room temperature for 15 minutes. Then the cells were rinsed in PBS for 3 times, 5 minutes for each time, and permeabilized in PBS containing 0.3% Triton X-100 at 4°C for 5 minutes, washed with PBS 3 times for 5 minutes, and prehybridized at 37°C for 30 minutes. Then 400 nM Cy3-labeled anti-Ntrk2 oligodeoxynucleotide probes (RiboBio) were added in the hybridization solution at 37°C overnight in the dark. The next day, cells were rinsed 3 times in 4× Saline Sodium Citrate (SSC) buffer for 5 minutes at 42°C, followed by washing once for 5 minutes at 42°C in 2× SSC and 1× SSC, respectively. Then the cells were blocked with 2% bovine serum albumin in PBS for 1 hour at room temperature and then incubated with rabbit anti-Tuj1 antibody overnight at 4°C. On the third day, after the samples were washed 3 times with PBS, they were incubated with Alexa Fluor 488 donkey anti-rabbit IgG (H+L) for 1 hour at room temperature. Then, the cells were washed 3 times in PBS. Finally, immunofluorescence images were captured using a Zeiss Axio Imager M2 microscope. FISH probes were designed and synthesized by RiboBio.

### Western blotting

Cultured DRG neurons or DRG tissues were lysed using RIPA buffer (Thermo Fisher Scientific), and the total protein was extracted according to the manufacturer’s instructions. For cytoplasmic and nuclear protein separation, nuclear and cytoplasmic proteins of 40 mg DRG tissue were isolated using an NE-PER Nuclear and Cytoplasmic Extraction Reagent kit (Thermo Fisher Scientific) according to the manufacturer’s instructions. Moreover, 400 μL cytoplasmic extraction regent I and 200 μL nuclear extraction reagent were used for each sample. Protease and phosphatase inhibitors were added to the extraction reagents before use. Protein concentration was determined using a Bicinchoninic Acid Protein Assay Kit (Thermo Fisher Scientific). Equal amounts (50 μg per sample) of protein were separated by 10% SDS-polyacrylamide gel electrophoresis and transferred onto polyvinylidene difluoride membranes (Roche Diagnostics). After blocking with 5% milk for 1 hour at room temperature, the membranes were incubated with primary antibodies (anti-GAPDH, anti-RSK1, anti-p-RSK1^S380^, anti-p-RSK1^S221^, anti-p-RSK1^T573^, anti-eEF2, anti-RSK2, anti-p-eEF2, anti-eEF2K, anti-p-eEF2K, anti-S6, anti-p-S6^S235/236^, or anti-Lamin B1) at 4°C overnight. Following incubation with horseradish peroxidase-conjugated secondary antibody for 1 hour at room temperature, protein bands were revealed using the ECL Western Blotting Detection Kit (Thermo Fisher Scientific). For the quantification of protein expression, all the blots were scanned at 600 dpi in TIFF file format and then converted to greyscale mode using Photoshop. The protein expression level was quantified densiometrically using ImageJ. The relative protein expression was calculated after normalization to GAPDH. Detailed antibody information is provided in S8 Table.

### ELISA for detection of BDNF and IGF1

Rat BDNF and IGF1 detection kits were obtained from Signalway Antibody (College Park, Maryland, USA). The secreted levels of BDNF and IGF1 in the supernatant of primary DRG neurons were determined following the manufacturer’s instructions. The results were recorded by measuring the absorbance at 450 nm in a BioTek Synergy 2 Plate Reader.

### GO enrichment analysis

The list of genes with down-regulated TEs in “TE only” and “Opposite” groups was submitted to the database for annotation, visualization, and integrated discovery (DAVID (2021 Update; https://david.ncifcrf.gov) and GO Resource (http://geneontology.org) for GO enrichment analysis[47–49]. Moreover, the top 10 enriched terms from intersection of the results (with the threshold of fold enrichment > 1.2, count ≥ 15) from the 2 different analysis tools are shown.

### Behavioral analysis

To evaluate the sensitivity to mechanical stimulation, the 50% paw withdrawal threshold was determined using the up-down method [80]. Briefly, rats were individually placed on a wire-mesh grid floor (5 × 5 mm) in a plastic cage. Following acclimation to the test cage for 1 hour, calibrated Von Frey filaments (TACTILE TEST AESTHESIO Semmes-Weinstein Von Frey Aesthesiometer, Muromachi Kikai, Tokyo, Japan) were applied to the middle of the plantar surface of the hind paw at an angle of 90° through the bottom of the mesh floor. In this paradigm, testing was initiated with a 10 *g* force in the middle of the series (4, 6, 8, 10, 15, 26, 60, and 100 *g*) and held for 3 to 5 seconds with the filament slightly buckled. Stimuli were always presented in a consecutive fashion, which was either ascending or descending. In the absence of a paw withdrawal response to the selected force, a stronger stimulus was applied. In the presence of paw withdrawal as a positive response, a weaker stimulus was chosen. After the response threshold was first crossed (the 2 responses straddling the threshold), 4 additional stimuli were applied. The 50% paw withdrawal threshold (g) was calculated based on the responses to the series of stimuli applied using the Von Frey filament. Rats that did not respond to any filaments following sciatic nerve crush injury were assigned a paw withdrawal threshold of 100 g.

The Hargreaves apparatus (Ugo Basile, Varese, Italy) was used to apply thermal stimulation to measure the sensitivity to thermal stimulation in unrestrained animals [81]. Rats were placed onto a plexiglass surface and left to acclimatize for 15 minutes before testing. The Hargreaves apparatus was set at 30% intensity, and 30 seconds was established as a cutoff time to avoid tissue damage. Rats were again tested before surgery to identify any abnormalities (and thus withdraw the rats from continued inclusion in the experiment), defined as a 30-second cutoff over the 3 repetitions, to avoid any potential burn injury (no rats were found with such abnormalities). For each rat, the heat source was applied to the plantar surface of the hind paw until the animal withdrew from the noxious thermal stimulus, and the time of reaction was measured. Ten minutes were allowed between each session. Measurements were repeated 3 times for each paw.

### Optic nerve injury and quantification

The procedure was performed as previously described [20]. Briefly, for the mouse experiment, 1 microliter of AAV-RSK1 or control AAV was intravitreally injected into the left eye of adult C57BL/6 or constitutive Rosa26-Cas-9 knock-in mice (stock number: JAX_026179) with 1 μL of AAV-PTEN-gRNA1–5; for the rat experiment, 2 microliters AAV-RSK1 or control AAV was intravitreally injected into the left eye of adult SD rats. Meloxicam (1 mg/kg) was injected as analgesia after the operation. Animals with obvious eye inflammation or shrinkage were sacrificed and excluded from further experiments. Two weeks after injection, an incision was made on the conjunctiva after the animals were anesthetized, the left optic nerve was intraorbitally exposed and crushed with jeweler’s forceps (Dumont number 5; Fine Science Tools) for 3 seconds, approximately 1 mm behind the optic disk. To visualize regenerating axons, RGC axons in the optic nerve were anterogradely labeled with 1 μL (for mice) or 4 μL (for rats) of cholera toxin B subunit (CTB; 2 mg/mL; Invitrogen) 12 days after injury. Animals were fixed in 4% PFA 2 days after CTB injection, and the fixed optic nerves were dehydrated in incremental concentrations of tetrahydrofuran (THF, 50%, 80%, 100%, and 100%, %v/v in distilled water, 20 minutes each, Sigma-Aldrich, St. Louis, Missouri, USA) in amber glass bottles on an orbital shaker at room temperature. Then the nerves were incubated with benzyl alcohol/benzyl benzoate (BABB, 1:2 in volume, Sigma-Aldrich) clearing solution for 20 minutes. The nerves were protected from exposure to light during the whole process to reduce photo bleaching of the fluorescence [82]. CTB fluorescence intensity was measured at different distances from the crush site and normalized to the intensity at the crush site.

### Statistical analysis

All animals and neuronal cultures used in these studies were randomly assigned to groups before treatment or any experimental manipulation. Sample size was calculated with G*Power 3.1 software, and values were set at *p* = 0.05, power = 0.8 and an effect size estimated from the previous experiments or pilot studies [9,59,83,84]. The numbers of independent animals are described in the Materials and methods and Results sections or indicated in the figure legends. All analyses were performed while blinded to the treatment group. Statistical analysis was performed with GraphPad Prism 8 using either the Student *t* test or ANOVA. One- and 2-way ANOVAs were followed by a Bonferroni, Tukey, or Dunnett post hoc test. Error bars indicate the standard error of the mean (SEM). A *p*-value < 0.05 was considered statistically significant.

## Supporting information

S1 Fig RSK inhibitors suppress DRG neuron regenerative growth. **Related to Fig 1. (A)** A CCK-8 assay showing the viability of cultured DRG neurons treated with various concentrations of rapamycin, eFT508, or SL0101 (N.S., not significant, mean ± SEM, 1-way ANOVA, Dunnett post hoc test, *n* = 3 biologically independent experiments). **(B)** CCK-8 assay showing the viability of cultured DRG neurons treated with various concentrations of BI-D1870 (BI) alone or combining with 10 μM SL0101 (SL) (N.S., not significant, mean ± SEM, 1-way ANOVA, Dunnett post hoc test, *n* = 3 biologically independent experiments). **(C)** Representative images of cultured DRG neurons treated with DMSO, 10 μM SL0101, 1 μM BI-D1870, or a combination of 0.5 μM BI-D1870 and 10 μM SL0101. Scale bar, 50 μm. **(D)** Quantification of the total and the longest neurite outgrowth length per neuron relating to (C) (mean ± SEM, 1-way ANOVA, Dunnett post hoc test, *n* = 4 biologically independent experiments, approximately 50 cells/experiment on average). The data underlying all the graphs shown in the figure are included in S1 Data. CCK-8, cell counting kit-8; DRG, dorsal root ganglion; RSK, ribosomal S6 kinase; SEM, standard error of the mean.
(TIF)

S2 Fig RSK1 expression and phosphorylation are up-regulated in DRG following sciatic nerve axotomy. **Related to Fig 2. (A)** Representative images of in situ hybridization for RSKs in DRG tissue sections on days 0 and 4 after nerve injury. The corresponding sense probe was used as a control (Sense) for nonspecific binding. Scale bar, 200 μm. **(B)** Quantification of RSKs intensity relating to (A) (mean ± SEM, unpaired 2-tailed *t* test, *n* = 4 biologically independent animals/group). **(C)** Western blotting showing RSK1 and RSK2 expression in DRG tissue after SNI. **(D)** Quantification of RSK1 and RSK2 expression levels relating to (C) (mean ± SEM, 1-way ANOVA, Dunnett post hoc test, *n* = 3 biologically independent experiments). **(E)** Western blotting showing RSK phosphorylation in DRG tissue after SNI. **(F)** Quantification of RSK phosphorylation levels relating to (E) (mean ± SEM, 1-way ANOVA, Dunnett post hoc test, *n* = 3 biologically independent experiments). **(G)** DRG tissues were fractionated into nuclear and cytoplasmic fractions at the indicated time points after SNI. The fractions were immunoblotted for p-RSK^S380^, p-RSK^T573^, p-RSK^S221^, GAPDH (cytoplasmic marker), and Lamin B1 (nuclear marker). **(H)** Quantification of RSK phosphorylation levels relating to (G) (mean ± SEM, 2-way ANOVA, Dunnett post hoc test, *n* = 3 biologically independent experiments). The data underlying all the graphs shown in the figure are included in S1 Data. DRG, dorsal root ganglion; RSK, ribosomal S6 kinase; RSK1, ribosomal S6 kinase 1; SEM, standard error of the mean; SNI, sciatic nerve injury.
(TIF)

S3 Fig RSK1 activities are up-regulated following sciatic nerve axotomy. **Related to Fig 2. (A, C)** Representative fluorescence images of immunostaining for p-S6^S235/236^ (A) and p-eEF2K (C) in the DRG on day 0, 1, or 4 post-SNI. Scale bar, 50 μm. **(B, D)** Quantification of p-S6^S235/236^ (B) and p-eEF2K (D) immunofluorescence intensity relating to (A) and (C) respectively. Relative protein expression levels were quantified after normalization to background immunofluorescence (secondary antibody only) (mean ± SEM, 1-way ANOVA, Dunnett post hoc test, *n* = 5 biologically independent animals/group). **(E)** Western blotting showing p-S6^S235/236^, total S6, p-eEF2K and total eEF2K expression in DRG tissue post-SNI. **(F, G)** Quantification of relative p-S6^S235/236^/S6 (F) and p-eEF2K/eEF2K (G) levels relating to (E) (mean ± SEM, 1-way ANOVA, Dunnett post hoc test, *n* = 3 biologically independent experiments). The data underlying all the graphs shown in the figure are included in S1 Data. DRG, dorsal root ganglion; RSK1, ribosomal S6 kinase 1; SEM, standard error of the mean; SNI, sciatic nerve injury.
(TIF)

S4 Fig Determination of efficiency and specificity of shRNAs targeting RSK1 in vitro. **Related to Fig 3. (A)** RT-qPCR analysis of the expression of RSK1 and RSK2 in DRG neurons infected with control AAV2/8 expressing scramble shRNA (Con) or AAV expressing shRNA1 (RSK1-sh1) or RSK1-sh2 (mean ± SEM, 1-way ANOVA, Dunnett post hoc test, *n* = 3 biologically independent experiments). **(B)** Western blotting showing RSK1 and RSK2 expression in DRG neurons infected with control AAV2/8 or AAV expressing RSK1-sh1 or RSK1-sh2. **(C)** Quantification of RSK1 and RSK2 levels relating to (B) (mean ± SEM, 1-way ANOVA, Dunnett post hoc test, *n* = 3 biologically independent experiments). **(D)** RT-qPCR analysis of the expression of potential candidate target genes of RSK1-sh2 (CACNA1S, EXOC2, PRKCA) in DRG neurons infected with control AAV2/8 or AAV expressing RSK1-sh2 (mean ± SEM, unpaired 2-tailed *t* test, *n* = 3 biologically independent experiments). The data underlying all the graphs shown in the figure are included in S1 Data. DRG, dorsal root ganglion; RSK1, ribosomal S6 kinase 1; RT-qPCR, reverse transcription quantitative real-time PCR; SEM, standard error of the mean.
(TIF)

S5 Fig Determination of AAV2/8 infection efficiency in vivo. **Related to Fig 3. (A)** EGFP (green) was co-labeled with a neuronal marker NeuN (red) in DRG at 2 weeks following intrathecal injection of AAV2/8 expressing EGFP. Scale bar, 200 μm. **(B)** Bar graph represents percentage of EGFP-positive neurons in all DRG neurons (mean ± SEM, *n* = 5 biologically independent animals). **(C–E)** EGFP (green) was co-labeled with NF200 (C), CGRP (D), or IB4 (E) (red) in DRG at 2 weeks following intrathecal injection of AAV2/8 expressing EGFP. Scale bar, 100 μm. **(F)** Bar graph represents percentage of EGFP-positive cells in the subsets of DRG neurons (mean ± SEM, *n* = 5 biologically independent animals). **(G)** Representative fluorescence images of EGFP (green) and RSK1(red) in the DRG infected with control AAV2/8 or AAV expressing RSK1-sh2. Scale bar, 50 μm. **(H)** Quantification of RSK1 immunofluorescence intensity in EGFP-positive cells relating to (G). Relative protein expression levels were quantified after normalization to background immunofluorescence (secondary antibody only) (mean ± SEM, unpaired 2-tailed *t* test, *n* = 5 biologically independent animals/group). The data underlying all the graphs shown in the figure are included in S1 Data. DRG, dorsal root ganglion; RSK1, ribosomal S6 kinase 1; SEM, standard error of the mean.
(TIF)

S6 Fig KD of RSK1 inhibits axon regeneration in a conditioning injury model. **Related to Fig 3. (A)** Timeline for RSK1 KD in a CL model. Briefly, AAV intrathecal injection and sciatic nerve transection or sham injury were performed simultaneously. Fourteen days later, a crush injury (the second injury) was performed approximately 8 mm proximal to the first injury site and sciatic nerve regrowth was analyzed after another 2 days. **(B)** Representative longitudinal sections from injured sciatic nerves. The crush site is indicated by a purple dotted line. Scale bar, 500 μm. **(C)** Normalized SCG10 intensity plotted in function of the distance from the crush line (*n* = 5 rats per group). **(D)** Axon regeneration in injured rats was quantified by regeneration indices obtained from SCG10 immunostaining on day 2 after crush injury (mean ± SEM, 1-way ANOVA, Tukey post hoc test, *n* = 5 rats per group). The data underlying all the graphs shown in the figure are included in S1 Data. CL, conditioning lesion; KD, knockdown; RSK1, ribosomal S6 kinase 1; SEM, standard error of the mean.
(TIF)

S7 Fig Determination of efficiency of AAV2/8 overexpressing RSK1 in vitro and in vivo. **Related to Fig 4. (A)** RT-qPCR analysis of the expression of RSK1 in primary DRG neurons infected with control AAV2/8 (Con) or AAV overexpressing wt-RSK1 (mean ± SEM, unpaired 2-tailed *t* test, *n* = 3 biologically independent experiments). **(B)** Western blotting analysis (upper panel) and quantification (lower panel) of RSK1 expression in primary DRG neurons infected with Con or wt-RSK1. **(C)** Western blotting showing p-eEF2K and total eEF2K expression in primary DRG neurons infected with Con, wt-RSK1, or AAV overexpressing inactive mutant (S221A, S380A, and T573A) RSK1 (iav-RSK1). **(D)** Quantification of relative p-eEF2K/eEF2K levels relating to (C) (mean ± SEM, 1-way ANOVA, Dunnett post hoc test, *n* = 3 biologically independent experiments). **(E)** Representative fluorescence images of RSK1 (green) and NeuN (red) in the DRG infected with Con or wt-RSK1. Scale bar, 50 μm. **(F)** Representative fluorescence images of p-RSK1^S221^ (green) and NeuN (red) in the DRG infected with Con or wt-RSK1. Scale bar, 25 μm. **(G)** Quantification of RSK1 and p-RSK1^S221^ immunofluorescence intensity in the soma relating to (E) and in the nuclei relating to (F), respectively. Relative protein expression levels were quantified after normalization to background immunofluorescence (secondary antibody only) (mean ± SEM, unpaired 2-tailed *t* test, *n* = 5 biologically independent animals/group). **(H)** Representative fluorescence images of p-eEF2K (green) and NeuN (red) in the DRG infected with Con, wt-RSK1 or iav-RSK1. Scale bar, 25 μm. **(I)** Quantification of p-eEF2K immunofluorescence intensity in the soma relating to (H). Relative protein expression levels were quantified after normalization to background immunofluorescence (secondary antibody only) (mean ± SEM, 1-way ANOVA, Tukey post hoc test, *n* = 5 biologically independent animals/group). The data underlying all the graphs shown in the figure are included in S1 Data. DRG, dorsal root ganglion; RSK1, ribosomal S6 kinase 1; RT-qPCR, reverse transcription quantitative real-time PCR; SEM, standard error of the mean; wt-RSK1, wild-type RSK1.
(TIF)

S8 Fig Determination of efficiency of AAV2/8 overexpressing eEF2 in vitro and in vivo. **Related to Fig 5. (A)** Western blotting showing p-eEF2 and total eEF2 expression in primary DRG neurons infected with control AAV2/8 (Con), AAV expressing wt-RSK1, or inactive mutant (S221A, S380A and T573A) RSK1 (iav-RSK1). **(B)** Quantification of relative p-eEF2/eEF2 levels relating to (A) (mean ± SEM, 1-way ANOVA, Tukey post hoc test, *n* = 3 biologically independent experiments). **(C)** RT-qPCR analysis of the expression of eEF2 in primary DRG neurons infected with control AAV2/8 (Con) or AAV overexpressing eEF2 (eEF2) (mean ± SEM, unpaired 2-tailed *t* test, *n* = 3 biologically independent experiments). **(D)** Western blotting showing eEF2 expression in DRG neurons infected with Con or eEF2. (E) Quantification of eEF2 levels relating to (D) (mean ± SEM, unpaired 2-tailed *t* test, *n* = 3 biologically independent experiments). **(F)** Representative fluorescence images of eEF2 (green) and NeuN (red) in the DRG infected with Con or eEF2. Scale bar, 25 μm. **(G)** Quantification of eEF2 immunofluorescence intensity in the soma relating to (F). Relative protein expression levels were quantified after normalization to background immunofluorescence (secondary antibody only) (mean ± SEM, unpaired 2-tailed *t* test, *n* = 5 biologically independent animals/group). **(H)** Western blotting showing RSK1 and eEF2 expression in DRG infected with control AAV (Con), AAV to knock down RSK1 (RSK1-sh2), or AAVs to knock down RSK1 and overexpress eEF2 (RSK1-sh2+eEF2). **(I, J)** Quantification of RSK1 (I) and eEF2 (J) levels relating to (H) (mean ± SEM, 1-way ANOVA, Tukey post hoc test, *n* = 3 biologically independent experiments). The data underlying all the graphs shown in the figure are included in S1 Data. DRG, dorsal root ganglion; RSK1, ribosomal S6 kinase 1; RT-qPCR, reverse transcription quantitative real-time PCR; SEM, standard error of the mean; wt-RSK1, wild-type RSK1.
(TIF)

S9 Fig Determination of efficiency and specificity of AAV2/8 expressing shRNA2 targeting RSK1 in vitro. **Related to Fig 6. (A)** EGFP (green) was co-labeled with a neuronal marker Tuj1 (red) and a nuclear staining dye DAPI (blue) in primary DRG neurons at 7 days following infection of AAV2/8 expressing shRNA2 targeting RSK1 (RSK1-sh2). Scale bar, 50 μm. **(B)** Bar graph represents percentages of EGFP-positive cells in neurons or nonneuronal cells (mean ± SEM, *n* = 5 biologically independent wells). **(C, D)** RNA-seq (C) and RT-qPCR (D) analysis of the expression of RSK1 in primary DRG neurons infected with control AAV2/8 expressing scramble shRNA (Con-sh) or AAV expressing RSK1-sh2 (mean ± SEM, unpaired 2-tailed *t* test, *n* = 3 biologically independent experiments). The data underlying all the graphs shown in the figure are included in S1 Data. RSK1, ribosomal S6 kinase 1; DRG, dorsal root ganglion; RT-qPCR, reverse transcription quantitative real-time PCR; SEM, standard error of the mean.
(TIF)

S10 Fig Determination of efficiency of AAV2/8 and capacity of the neutralizing antibodies against BDNF and IGF1. **Related to Fig 7. (A)** RT-qPCR analysis of the expression of RSK1 in primary DRG neurons infected with control AAV2/8 expressing scramble shRNA (Con-sh) or AAV expressing shRNA2 (RSK1-sh2) (mean ± SEM, unpaired 2-tailed *t* test, *n* = 3 biologically independent experiments). **(B)** RT-qPCR analysis of the expression of RSK1 in primary DRG neurons infected with control AAV2/8 (Con-OE) or AAV overexpressing RSK1 (RSK1-OE) (mean ± SEM, unpaired 2-tailed *t* test, *n* = 3 biologically independent experiments). **(C)** Representative images of cultured DRG neurons treated with PBS (Mock), 10 μg/mL IgG, 5 ng/mL BDNF and 10 μg/mL IgG (BDNF+IgG), 5 ng/mL BDNF and 10 μg/mL BDNF neutralizing antibody (BDNF+a-BDNF), 10 ng/mL IGF1 and 10 μg/mL IgG (IGF1+IgG), 10 ng/mL IGF1 and 10 μg/mL IGF1 neutralizing antibody (IGF1+a-IGF1). Scale bar, 50 μm. **(D)** Quantification of the total and the longest neurite outgrowth per neuron relating to (C) (mean ± SEM, 1-way ANOVA, Tukey post hoc test, *n* = 4 biologically independent experiments, approximately 50 cells/experiment on average). **(E)** Representative images of cultured DRG neurons infected with control AAV2/8 (Con-OE) or AAV overexpressing RSK1 (RSK1-OE). Red signals show the TrkB^+^ cells by FISH, whereas the green signals show the Tuj1^+^ cells by IHC. Scale bar, 100 μm. **(F)** Quantification of the total and the longest neurite outgrowth per neuron relating to (E) (mean ± SEM, 1-way ANOVA, Bonferroni post hoc test, *n* = 4 biologically independent experiments, approximately 50 cells/experiment on average). The data underlying all the graphs shown in the figure are included in S1 Data. DRG, dorsal root ganglion; RSK1, ribosomal S6 kinase 1; RT-qPCR, reverse transcription quantitative real-time PCR; SEM, standard error of the mean.
(TIF)

S11 Fig RSK1 expression and activity are unchanged in RGCs following optic nerve axotomy. **Related to Fig 8. (A–D)** Representative fluorescence images of immunostaining for RSK1 (A), p-RSK^S380^ (B), p-RSK^T573^ (C), and p-RSK^S221^ (D) (red) in the retina at 0, 1 or 3 days post-ONC injury. Tuj1 (green) was used to label RGCs. Scale bar, 40 μm. **(E)** Quantification of RSK1, p-RSK^S380^, p-RSK^T573^, and p-RSK^S221^ immunofluorescence intensity relating to (A–D), respectively. Relative protein expression levels were quantified after normalization to background immunofluorescence (secondary antibody only) (mean ± SEM, 1-way ANOVA, Dunnett post hoc test, *n* = 5 biologically independent animals/group). The data underlying all the graphs shown in the figure are included in S1 Data. ONC, optic nerve crush; RGC, retinal ganglion cell; RSK1, ribosomal S6 kinase 1; SEM, standard error of the mean.
(TIF)

S12 Fig RSK1 alone does not affect axon regeneration in rat RGCs. **Related to Fig 8. (A)** EGFP (green) was co-labeled with a RGC marker Tuj1 (red) in rat retinas at 2 weeks following intravitreal injection of AAV2 expressing EGFP. Scale bar, 40 μm. **(B)** Bar graph represents percentage of EGFP-positive neurons in RGCs (mean ± SEM, *n* = 4 biologically independent animals). **(C)** Representative fluorescence images of Tuj1 (green) and RSK1 (red) in the retina infected with control AAV2 (Con), or AAV expressing wt-RSK1. Scale bar, 40 μm. **(D)** Quantification of RSK1 immunofluorescence intensity in RGCs relating to (C). Relative protein expression levels were quantified after normalization to background immunofluorescence (secondary antibody only) (mean ± SEM, unpaired 2-tailed *t* test, *n* = 4 biologically independent animals/group). **(E)** Representative fluorescence images of Tuj1 (green) and p-eEF2K (red) in the retina infected with control AAV2 (Con), AAVs expressing wt-RSK1 or active mutant (S221D, S380D, and T573D) RSK1 (av-RSK1). Scale bar, 40 μm. **(F)** Quantification of p-eEF2K immunofluorescence intensity in RGCs relating to (E) (mean ± SEM, 1-way ANOVA, Tukey post hoc test, *n* = 4 biologically independent animals/group). **(G)** Timeline for RSK1 overexpression in rat RGCs, ONC injury, and CTB injection. **(H)** Representative images of the cleared whole-mount rat optic nerves 2 weeks postinjury. Control AAV2 (Con), AAVs expressing wt-RSK1 or av-RSK1 were administered by intravitreal injection. Axons were labeled by AF 555-conjugated CTB. Scale bar, 250 μm. **(I)** Normalized fluorescence intensity plotted in function of the distance from the crush line (N.S., not significant, mean ± SEM, 2-way ANOVA, Tukey post hoc test, *n* = 5 rats per group). The data underlying all the graphs shown in the figure are included in S1 Data. AF, Alexa Fluor; CTB, cholera toxin B subunit; ONC, optic nerve crush; RGC, retinal ganglion cell; RSK1, ribosomal S6 kinase 1; SEM, standard error of the mean; wt-RSK1, wild-type RSK1.
(TIF)

S13 Fig RSK1 alone does not affect axon regeneration in mouse RGCs. **Related to Fig 8. (A)** Representative fluorescence images of Tuj1 (green) and RSK1 (red) in mouse retina infected with control AAV2 (Con), or AAV expressing wt-RSK1. Scale bar, 40 μm. **(B)** Quantification of RSK1 immunofluorescence intensity in mouse RGCs relating to (A). Relative protein expression levels were quantified after normalization to background immunofluorescence (secondary antibody only) (mean ± SEM, unpaired 2-tailed *t* test, *n* = 4 biologically independent animals/group). **(C)** Representative fluorescence images of Tuj1 (green) and p-eEF2K (red) in mouse retina infected with control AAV2 (Con), AAV expressing wt-RSK1or av-RSK1. Scale bar, 40 μm. **(D)** Quantification of p-eEF2K immunofluorescence intensity in RGCs relating to (C) (mean ± SEM, 1-way ANOVA, Tukey post hoc test, *n* = 4 biologically independent animals/group). **(E)** Representative images of the cleared whole-mount mouse optic nerves 2 weeks postinjury. Control AAV2 (Con), AAVs expressing wt-RSK1 or av-RSK1 were administered by intravitreal injection. Axons were labeled by AF 555-conjugated CTB. Scale bar, 250 μm. **(F)** Normalized fluorescence intensity plotted in function of the distance from the crush line (N.S., not significant, mean ± SEM, 2-way ANOVA, Tukey post hoc test, *n* = 5 mice per group). **(G)** Representative fluorescence images of Tuj1 (green) and p-S6^S240/244^ (red) in Rosa26-Cas-9 mouse retina infected with control AAV2 (Con), or AAV with gRNA targeting PTEN (PTEN KO) followed by ONC for 2 weeks. Scale bar, 40 μm. **(H)** Quantification of p-S6^S240/244^ immunofluorescence intensity in RGCs relating to (G) (mean ± SEM, unpaired 2-tailed *t* test, *n* = 4 biologically independent animals/group). The data underlying all the graphs shown in the figure are included in S1 Data. AF, Alexa Fluor; CTB, cholera toxin B subunit; gRNA, guide RNA; ONC, optic nerve crush; RGC, retinal ganglion cell; RSK1, ribosomal S6 kinase 1; SEM, standard error of the mean; wt-RSK1, wild-type RSK1.
(TIF)

S1 Data The underlying data for Figs 1C, 2B, 2D, 2H, 3D, 3G, 3H, 4D, 4G, 4H, 4I, 4J, 5D, 5G, 5H, 5I, 5J, 6C, 6E, 7A, 7B, 7C, 7D, 7G and 8C and S1A, S1B, S1D, S2B, S2D, S2F, S2H, S3B, S3D, S3F, S3G, S4A, S4C, S4D, S5B, S5F, S5H, S6C, S6D, S7A, S7B, S7D, S7G, S7I, S8B, S8C, S8E, S8G, S8I, S8J, S9B, S9C, S9D, S10A, S10B, S10D, S10F, S11E, S12B, S12D, S12F, S12I, S13B, S13D, S13F and S13H Figs. (XLSX)

S1 Raw images Original blot images for S2C, S2E, S2G, S3E, S4B, S7B, S7C, S8A, S8D and S8H Figs. (PPTX)

S1 Table List of differential genes regulated by RSK1 in translatome and transcriptome. Related to Fig 6B. RSK1, ribosomal S6 kinase 1.
(XLSX)

S2 Table List of differential genes regulated by RSK1 in TEs. Related to Fig 6D. RSK1, ribosomal S6 kinase 1; TE, translation efficiency.
(XLSX)

S3 Table Intersection of the results from GO analysis using DAVID and GO Resource. Related to Fig 6E. GO, Gene Ontology.
(XLSX)

S4 Table Frequency of the genes in top 10 enriched GO terms. Related to Fig 6E.
(XLSX)

S5 Table List of shRNA and sgRNA sequences. (DOCX)

S6 Table List of qRT-PCR primers. (DOCX)

S7 Table List of cDNAs used for in situ hybridization. (DOCX)

S8 Table List of the antibodies. (DOCX)

## Abbreviations

BCIP-NBT: 5-bromo-4-chloro-3-indolyl phosphate and nitroblue tetrazolium
CNS: central nervous system
CTB: cholera toxin B subunit
CTK: carboxyl-terminal kinase
DIG: digoxigenin
DIV1: 1 day in vitro
DRG: dorsal root ganglion
ELISA: enzyme-linked immunosorbent assay
FISH: fluorescence in situ hybridization
GC: genome copies
GO: Gene Ontology
gRNA: guide RNA
hSyn: human synapsin
IHC: immunohistochemistry
iav-RSK1: inactive RSK1
KD: knockdown
KO: knockout
MAPK: mitogen-activated protein kinase
MAPKAPK: MAPK-activated protein kinase
MNK: MAPK-interacting kinase
mTOR: mechanistic target of rapamycin
NTK: N-terminal kinase
ONC: optic nerve crush
PFA: paraformaldehyde
PNS: peripheral nervous system
PTEN: phosphatase and tensin homolog
RGC: retinal ganglion cell
RPF: ribosome-protected fragment
RSK: ribosomal S6 kinase
RSK1: ribosomal S6 kinase 1
RT-qPCR: reverse transcription quantitative real-time PCR
SD: Sprague–Dawley
SEM: standard error of the mean
SNI: sciatic nerve injury
SSC: Saline Sodium Citrate
TE: translation efficiency
THF: tetrahydrofuran
wt-RSK1: wild-type RSK1
