# Supplementary figures and images for: RSK1 promotes mammalian axon regeneration by inducing the synthesis of regeneration-related proteins
Source: PLoS Biol. 2022 Jun 1;20(6):e3001653. doi: 10.1371/journal.pbio.3001653 (PMC9159620; doi:10.1371/journal.pbio.3001653)

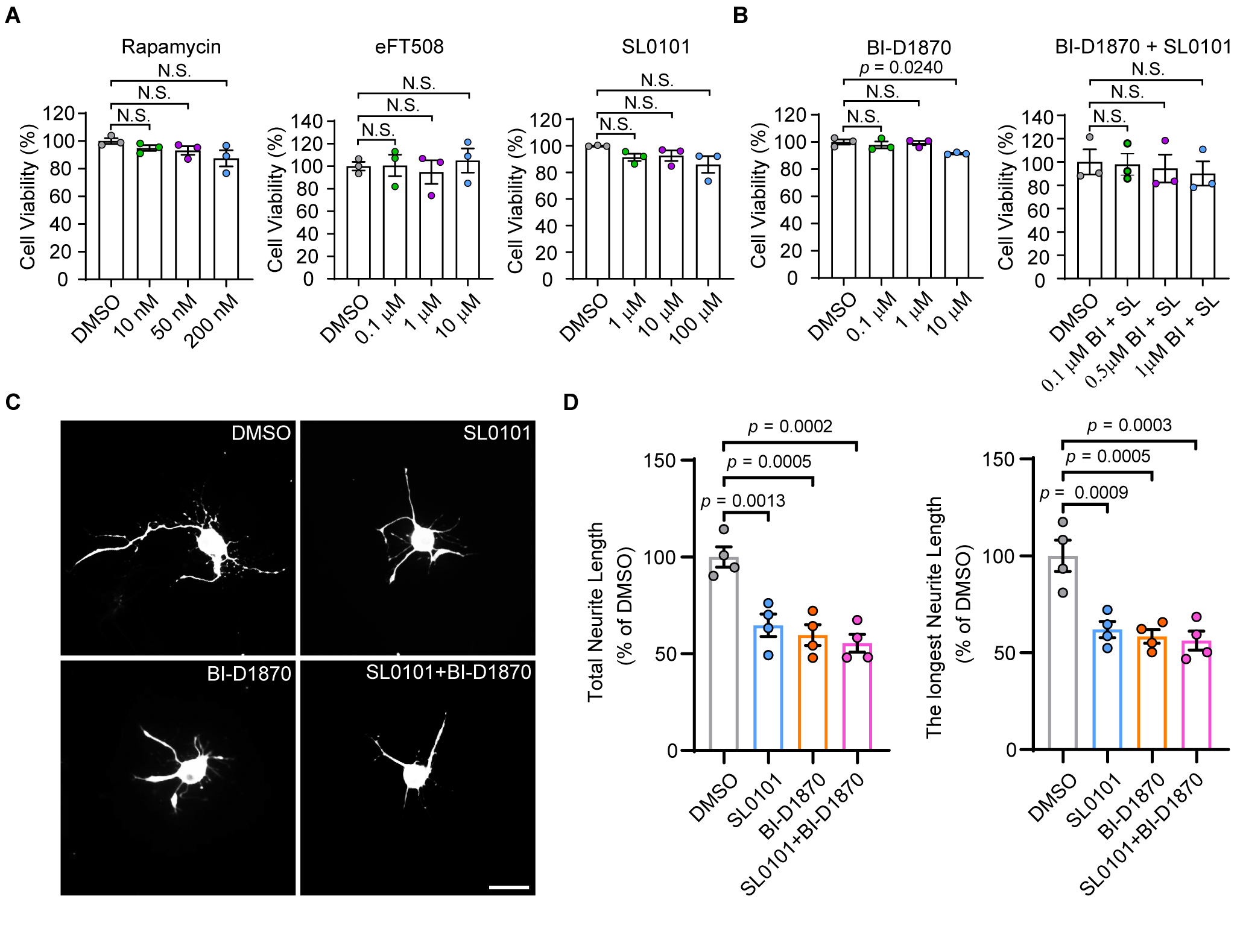

Supplement: S1 Fig — Related to Fig 1. (A) A CCK-8 assay showing the viability of cultured DRG neurons treated with various concentrations of rapamycin, eFT508, or SL0101 (N.S., not significant, mean ± SEM, 1-way ANOVA, Dunnett post hoc test, n = 3 biologically independent experiments). (B) CCK-8 assay showing the viability of cultured DRG neurons treated with various concentrations of BI-D1870 (BI) alone or combining with 10 μM SL0101 (SL) (N.S., not significant, mean ± SEM, 1-way ANOVA, Dunnett post hoc test, n = 3 biologically independent experiments). (C) Representative images of cultured DRG neurons treated with DMSO, 10 μM SL0101, 1 μM BI-D1870, or a combination of 0.5 μM BI-D1870 and 10 μM SL0101. Scale bar, 50 μm. (D) Quantification of the total and the longest neurite outgrowth length per neuron relating to (C) (mean ± SEM, 1-way ANOVA, Dunnett post hoc test, n = 4 biologically independent experiments, approximately 50 cells/experiment on average). The data underlying all the graphs shown in the figure are included in S1 Data. CCK-8, cell counting kit-8; DRG, dorsal root ganglion; RSK, ribosomal S6 kinase; SEM, standard error of the mean. (TIF) [file pbio.3001653.s001.tif]

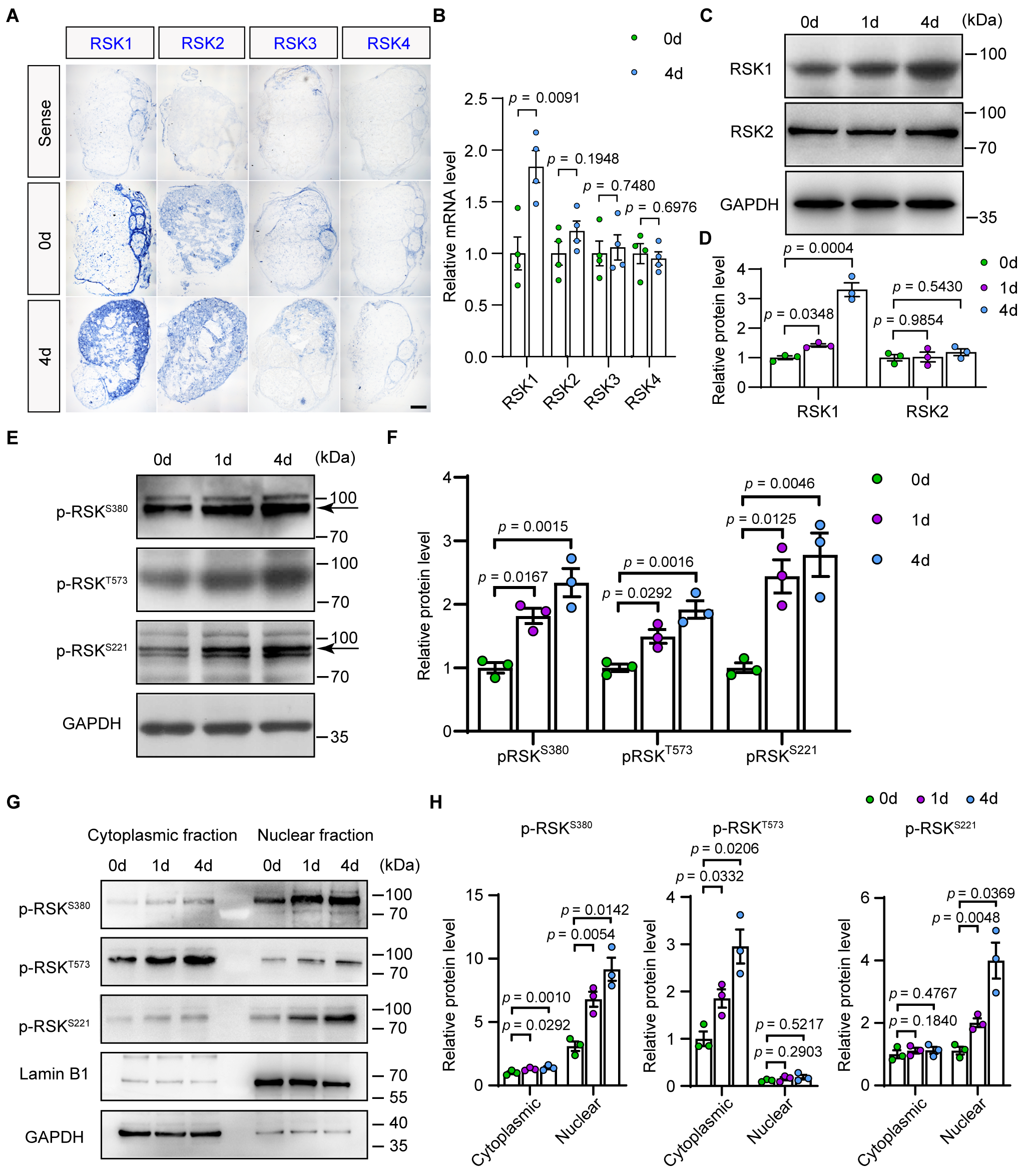

Supplement: S2 Fig — Related to Fig 2. (A) Representative images of in situ hybridization for RSKs in DRG tissue sections on days 0 and 4 after nerve injury. The corresponding sense probe was used as a control (Sense) for nonspecific binding. Scale bar, 200 μm. (B) Quantification of RSKs intensity relating to (A) (mean ± SEM, unpaired 2-tailed t test, n = 4 biologically independent animals/group). (C) Western blotting showing RSK1 and RSK2 expression in DRG tissue after SNI. (D) Quantification of RSK1 and RSK2 expression levels relating to (C) (mean ± SEM, 1-way ANOVA, Dunnett post hoc test, n = 3 biologically independent experiments). (E) Western blotting showing RSK phosphorylation in DRG tissue after SNI. (F) Quantification of RSK phosphorylation levels relating to (E) (mean ± SEM, 1-way ANOVA, Dunnett post hoc test, n = 3 biologically independent experiments). (G) DRG tissues were fractionated into nuclear and cytoplasmic fractions at the indicated time points after SNI. The fractions were immunoblotted for p-RSKS380, p-RSKT573, p-RSKS221, GAPDH (cytoplasmic marker), and Lamin B1 (nuclear marker). (H) Quantification of RSK phosphorylation levels relating to (G) (mean ± SEM, 2-way ANOVA, Dunnett post hoc test, n = 3 biologically independent experiments). The data underlying all the graphs shown in the figure are included in S1 Data. DRG, dorsal root ganglion; RSK, ribosomal S6 kinase; RSK1, ribosomal S6 kinase 1; SEM, standard error of the mean; SNI, sciatic nerve injury. (TIF) [file pbio.3001653.s002.tif]

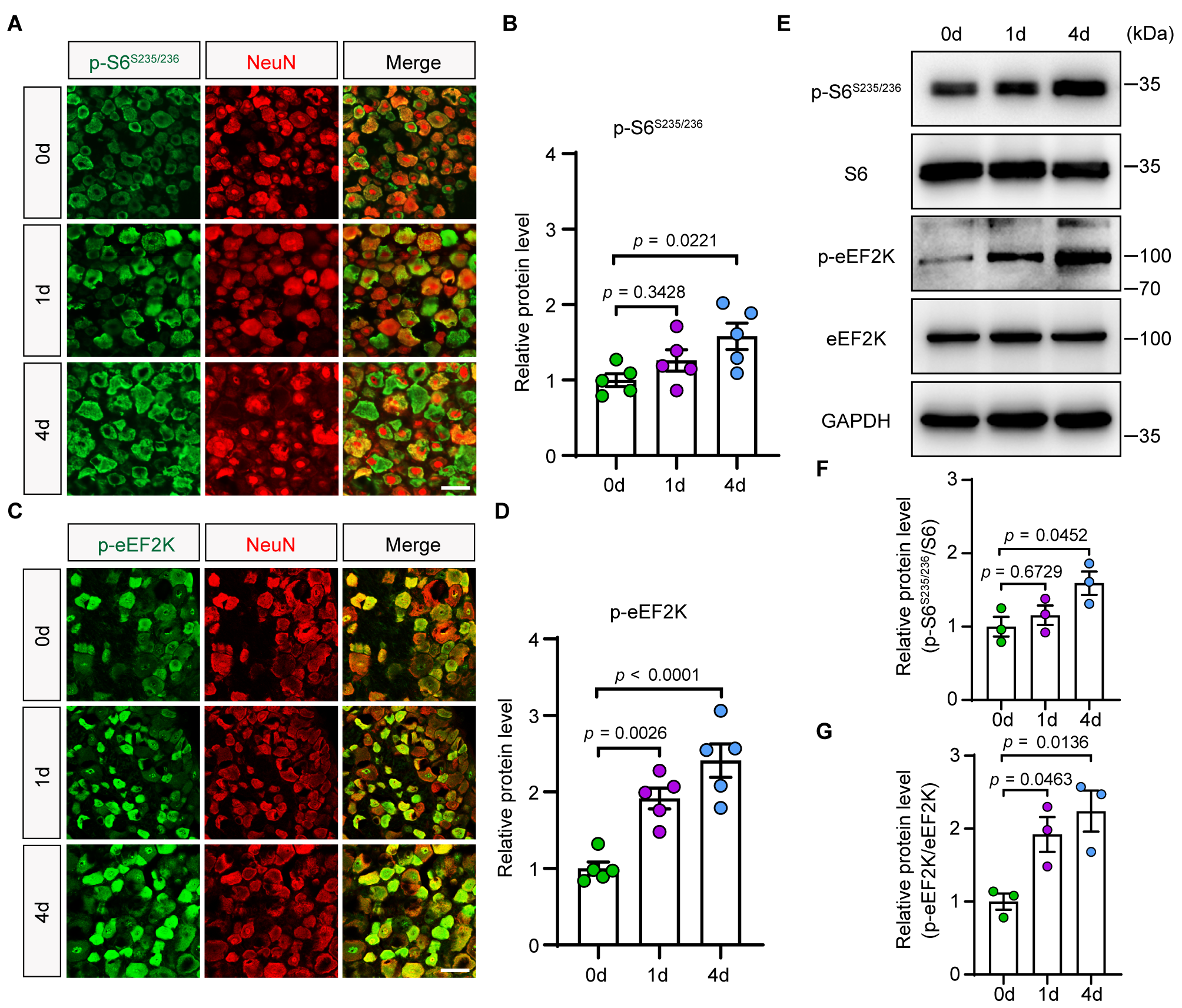

Supplement: S3 Fig — Related to Fig 2. (A, C) Representative fluorescence images of immunostaining for p-S6S235/236 (A) and p-eEF2K (C) in the DRG on day 0, 1, or 4 post-SNI. Scale bar, 50 μm. (B, D) Quantification of p-S6S235/236 (B) and p-eEF2K (D) immunofluorescence intensity relating to (A) and (C) respectively. Relative protein expression levels were quantified after normalization to background immunofluorescence (secondary antibody only) (mean ± SEM, 1-way ANOVA, Dunnett post hoc test, n = 5 biologically independent animals/group). (E) Western blotting showing p-S6S235/236, total S6, p-eEF2K and total eEF2K expression in DRG tissue post-SNI. (F, G) Quantification of relative p-S6S235/236/S6 (F) and p-eEF2K/eEF2K (G) levels relating to (E) (mean ± SEM, 1-way ANOVA, Dunnett post hoc test, n = 3 biologically independent experiments). The data underlying all the graphs shown in the figure are included in S1 Data. DRG, dorsal root ganglion; RSK1, ribosomal S6 kinase 1; SEM, standard error of the mean; SNI, sciatic nerve injury. (TIF) [file pbio.3001653.s003.tif]

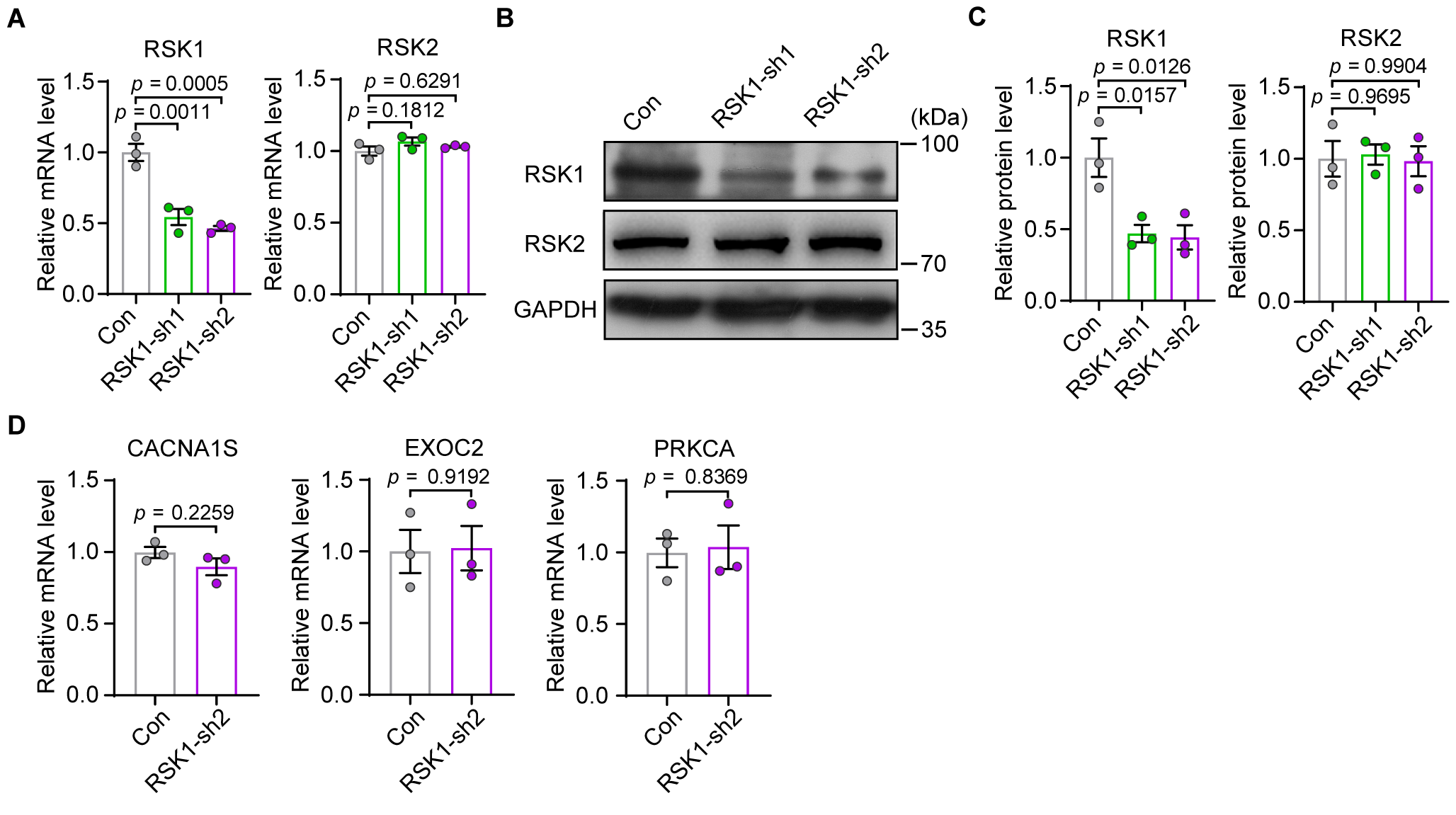

Supplement: S4 Fig — Related to Fig 3. (A) RT-qPCR analysis of the expression of RSK1 and RSK2 in DRG neurons infected with control AAV2/8 expressing scramble shRNA (Con) or AAV expressing shRNA1 (RSK1-sh1) or RSK1-sh2 (mean ± SEM, 1-way ANOVA, Dunnett post hoc test, n = 3 biologically independent experiments). (B) Western blotting showing RSK1 and RSK2 expression in DRG neurons infected with control AAV2/8 or AAV expressing RSK1-sh1 or RSK1-sh2. (C) Quantification of RSK1 and RSK2 levels relating to (B) (mean ± SEM, 1-way ANOVA, Dunnett post hoc test, n = 3 biologically independent experiments). (D) RT-qPCR analysis of the expression of potential candidate target genes of RSK1-sh2 (CACNA1S, EXOC2, PRKCA) in DRG neurons infected with control AAV2/8 or AAV expressing RSK1-sh2 (mean ± SEM, unpaired 2-tailed t test, n = 3 biologically independent experiments). The data underlying all the graphs shown in the figure are included in S1 Data. DRG, dorsal root ganglion; RSK1, ribosomal S6 kinase 1; RT-qPCR, reverse transcription quantitative real-time PCR; SEM, standard error of the mean. (TIF) [file pbio.3001653.s004.tif]

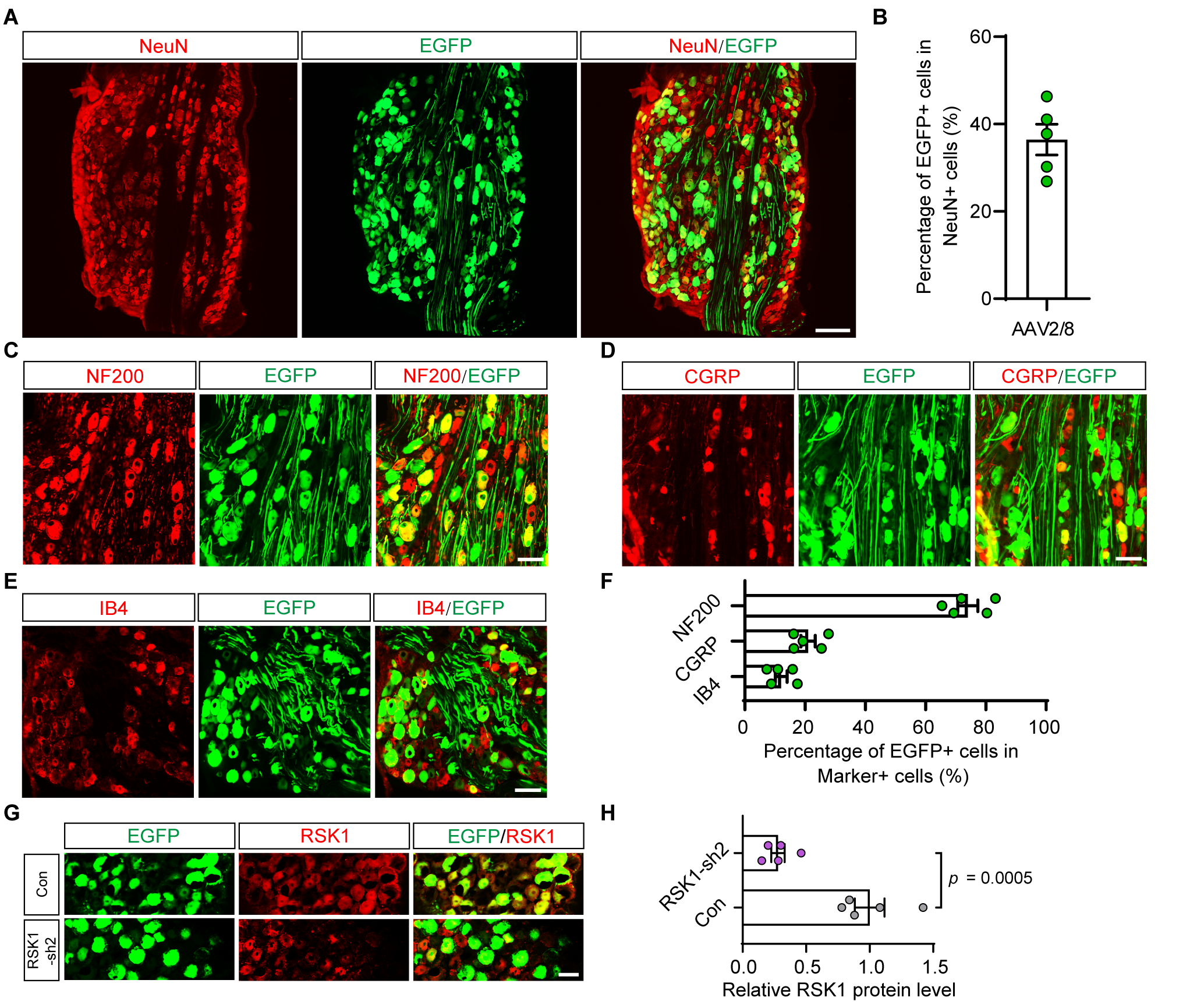

Supplement: S5 Fig — Related to Fig 3. (A) EGFP (green) was co-labeled with a neuronal marker NeuN (red) in DRG at 2 weeks following intrathecal injection of AAV2/8 expressing EGFP. Scale bar, 200 μm. (B) Bar graph represents percentage of EGFP-positive neurons in all DRG neurons (mean ± SEM, n = 5 biologically independent animals). (C–E) EGFP (green) was co-labeled with NF200 (C), CGRP (D), or IB4 (E) (red) in DRG at 2 weeks following intrathecal injection of AAV2/8 expressing EGFP. Scale bar, 100 μm. (F) Bar graph represents percentage of EGFP-positive cells in the subsets of DRG neurons (mean ± SEM, n = 5 biologically independent animals). (G) Representative fluorescence images of EGFP (green) and RSK1(red) in the DRG infected with control AAV2/8 or AAV expressing RSK1-sh2. Scale bar, 50 μm. (H) Quantification of RSK1 immunofluorescence intensity in EGFP-positive cells relating to (G). Relative protein expression levels were quantified after normalization to background immunofluorescence (secondary antibody only) (mean ± SEM, unpaired 2-tailed t test, n = 5 biologically independent animals/group). The data underlying all the graphs shown in the figure are included in S1 Data. DRG, dorsal root ganglion; RSK1, ribosomal S6 kinase 1; SEM, standard error of the mean. (TIF) [file pbio.3001653.s005.tif]

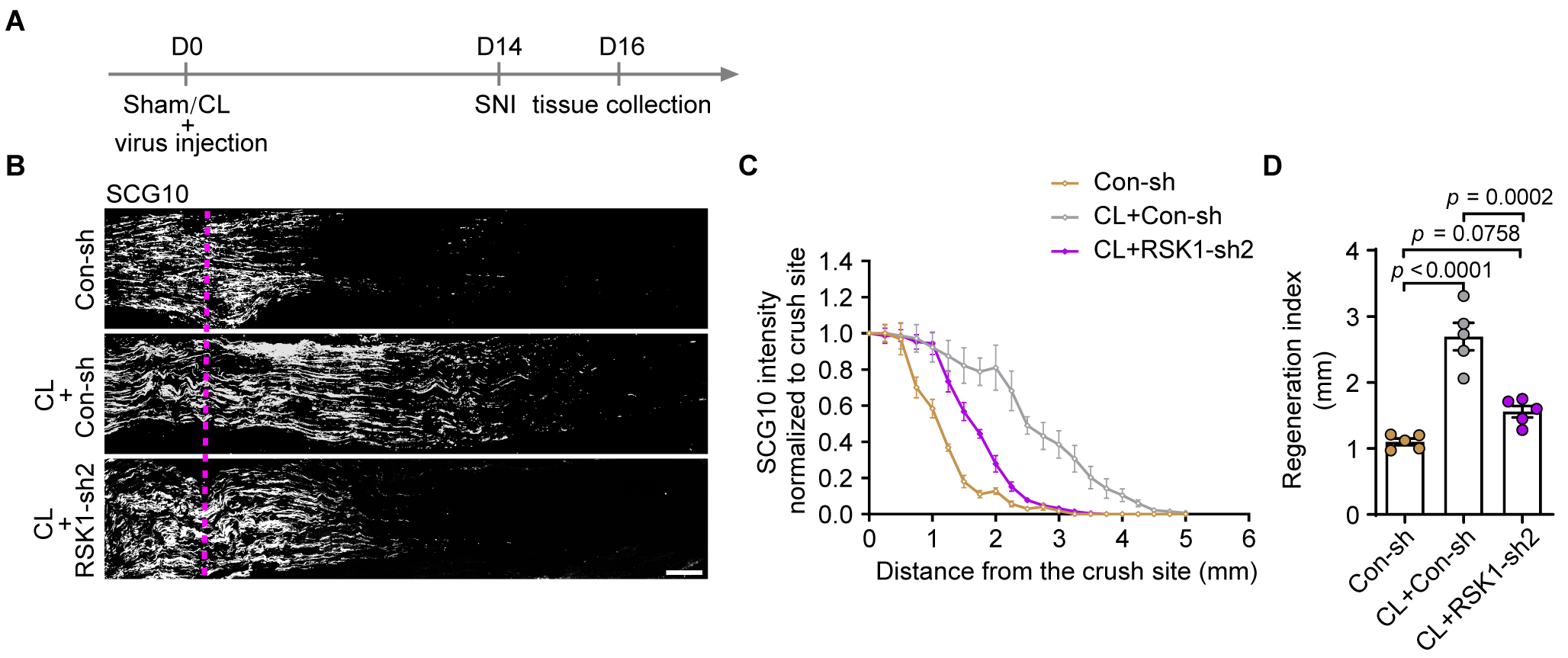

Supplement: S6 Fig — Related to Fig 3. (A) Timeline for RSK1 KD in a CL model. Briefly, AAV intrathecal injection and sciatic nerve transection or sham injury were performed simultaneously. Fourteen days later, a crush injury (the second injury) was performed approximately 8 mm proximal to the first injury site and sciatic nerve regrowth was analyzed after another 2 days. (B) Representative longitudinal sections from injured sciatic nerves. The crush site is indicated by a purple dotted line. Scale bar, 500 μm. (C) Normalized SCG10 intensity plotted in function of the distance from the crush line (n = 5 rats per group). (D) Axon regeneration in injured rats was quantified by regeneration indices obtained from SCG10 immunostaining on day 2 after crush injury (mean ± SEM, 1-way ANOVA, Tukey post hoc test, n = 5 rats per group). The data underlying all the graphs shown in the figure are included in S1 Data. CL, conditioning lesion; KD, knockdown; RSK1, ribosomal S6 kinase 1; SEM, standard error of the mean. (TIF) [file pbio.3001653.s006.tif]

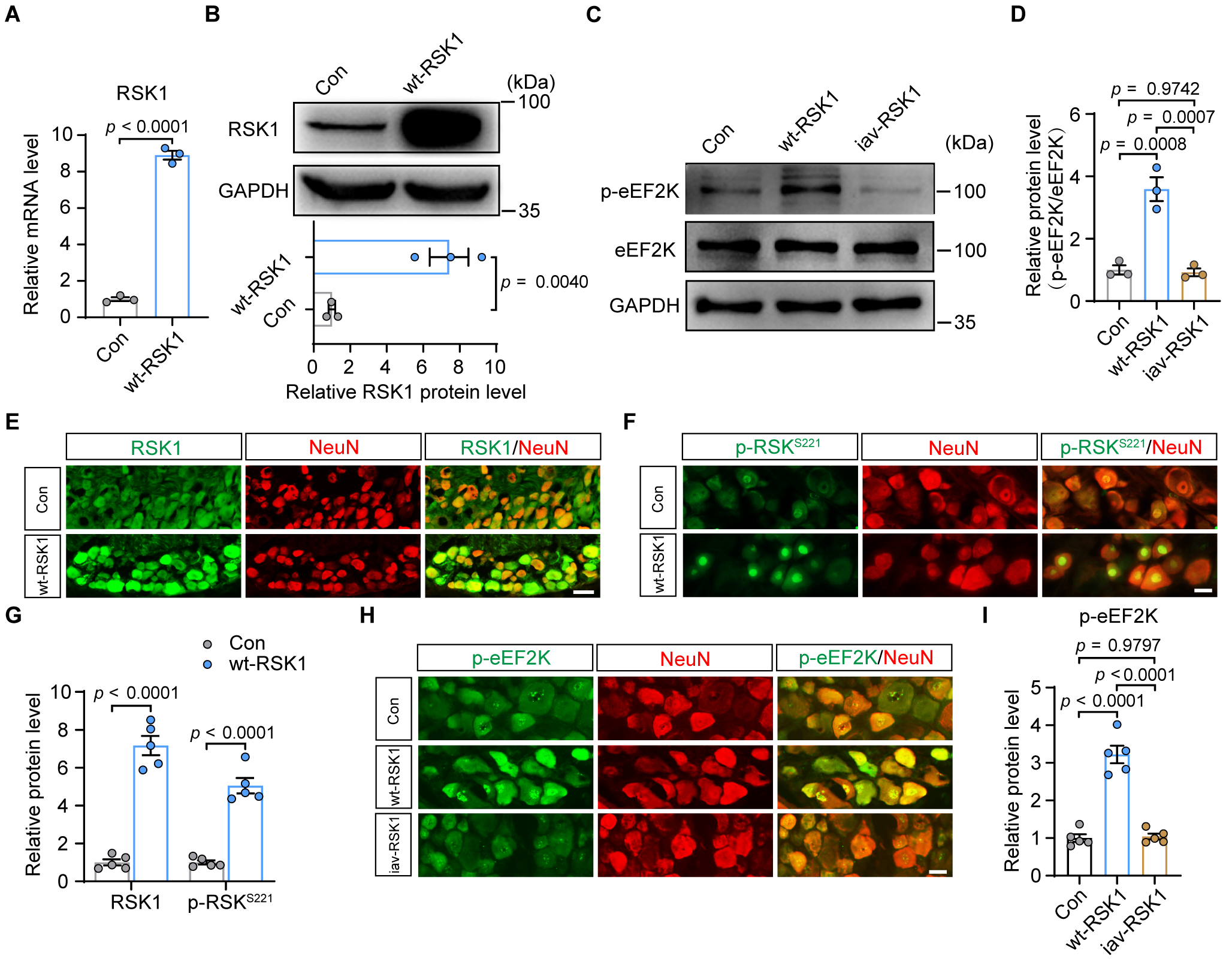

Supplement: S7 Fig — Related to Fig 4. (A) RT-qPCR analysis of the expression of RSK1 in primary DRG neurons infected with control AAV2/8 (Con) or AAV overexpressing wt-RSK1 (mean ± SEM, unpaired 2-tailed t test, n = 3 biologically independent experiments). (B) Western blotting analysis (upper panel) and quantification (lower panel) of RSK1 expression in primary DRG neurons infected with Con or wt-RSK1. (C) Western blotting showing p-eEF2K and total eEF2K expression in primary DRG neurons infected with Con, wt-RSK1, or AAV overexpressing inactive mutant (S221A, S380A, and T573A) RSK1 (iav-RSK1). (D) Quantification of relative p-eEF2K/eEF2K levels relating to (C) (mean ± SEM, 1-way ANOVA, Dunnett post hoc test, n = 3 biologically independent experiments). (E) Representative fluorescence images of RSK1 (green) and NeuN (red) in the DRG infected with Con or wt-RSK1. Scale bar, 50 μm. (F) Representative fluorescence images of p-RSK1S221 (green) and NeuN (red) in the DRG infected with Con or wt-RSK1. Scale bar, 25 μm. (G) Quantification of RSK1 and p-RSK1S221 immunofluorescence intensity in the soma relating to (E) and in the nuclei relating to (F), respectively. Relative protein expression levels were quantified after normalization to background immunofluorescence (secondary antibody only) (mean ± SEM, unpaired 2-tailed t test, n = 5 biologically independent animals/group). (H) Representative fluorescence images of p-eEF2K (green) and NeuN (red) in the DRG infected with Con, wt-RSK1 or iav-RSK1. Scale bar, 25 μm. (I) Quantification of p-eEF2K immunofluorescence intensity in the soma relating to (H). Relative protein expression levels were quantified after normalization to background immunofluorescence (secondary antibody only) (mean ± SEM, 1-way ANOVA, Tukey post hoc test, n = 5 biologically independent animals/group). The data underlying all the graphs shown in the figure are included in S1 Data. DRG, dorsal root ganglion; RSK1, ribosomal S6 kinase 1; RT-qPCR, reverse transcription quantit [file pbio.3001653.s007.tif]

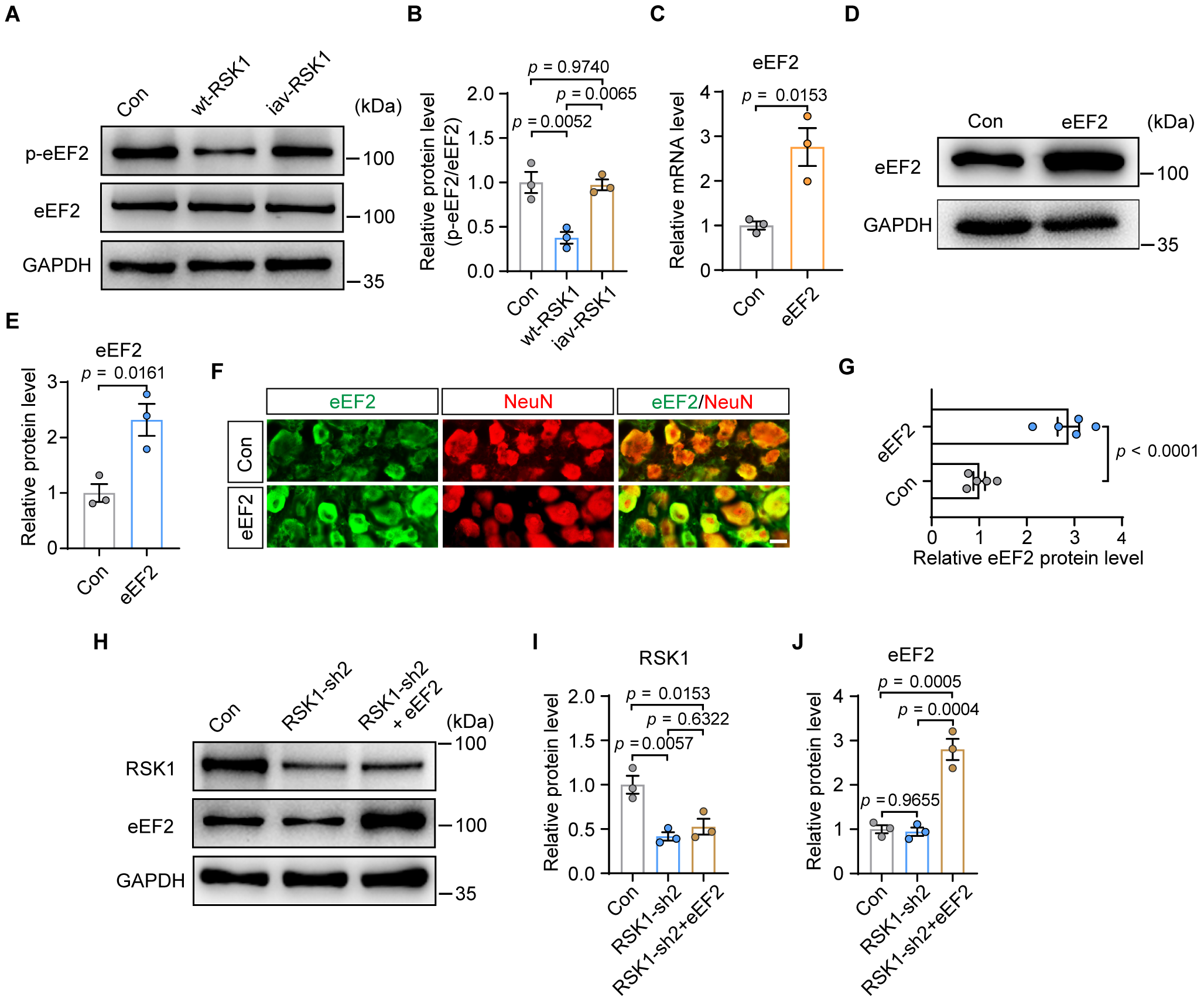

Supplement: S8 Fig — Related to Fig 5. (A) Western blotting showing p-eEF2 and total eEF2 expression in primary DRG neurons infected with control AAV2/8 (Con), AAV expressing wt-RSK1, or inactive mutant (S221A, S380A and T573A) RSK1 (iav-RSK1). (B) Quantification of relative p-eEF2/eEF2 levels relating to (A) (mean ± SEM, 1-way ANOVA, Tukey post hoc test, n = 3 biologically independent experiments). (C) RT-qPCR analysis of the expression of eEF2 in primary DRG neurons infected with control AAV2/8 (Con) or AAV overexpressing eEF2 (eEF2) (mean ± SEM, unpaired 2-tailed t test, n = 3 biologically independent experiments). (D) Western blotting showing eEF2 expression in DRG neurons infected with Con or eEF2. (E) Quantification of eEF2 levels relating to (D) (mean ± SEM, unpaired 2-tailed t test, n = 3 biologically independent experiments). (F) Representative fluorescence images of eEF2 (green) and NeuN (red) in the DRG infected with Con or eEF2. Scale bar, 25 μm. (G) Quantification of eEF2 immunofluorescence intensity in the soma relating to (F). Relative protein expression levels were quantified after normalization to background immunofluorescence (secondary antibody only) (mean ± SEM, unpaired 2-tailed t test, n = 5 biologically independent animals/group). (H) Western blotting showing RSK1 and eEF2 expression in DRG infected with control AAV (Con), AAV to knock down RSK1 (RSK1-sh2), or AAVs to knock down RSK1 and overexpress eEF2 (RSK1-sh2+eEF2). (I, J) Quantification of RSK1 (I) and eEF2 (J) levels relating to (H) (mean ± SEM, 1-way ANOVA, Tukey post hoc test, n = 3 biologically independent experiments). The data underlying all the graphs shown in the figure are included in S1 Data. DRG, dorsal root ganglion; RSK1, ribosomal S6 kinase 1; RT-qPCR, reverse transcription quantitative real-time PCR; SEM, standard error of the mean; wt-RSK1, wild-type RSK1. (TIF) [file pbio.3001653.s008.tif]

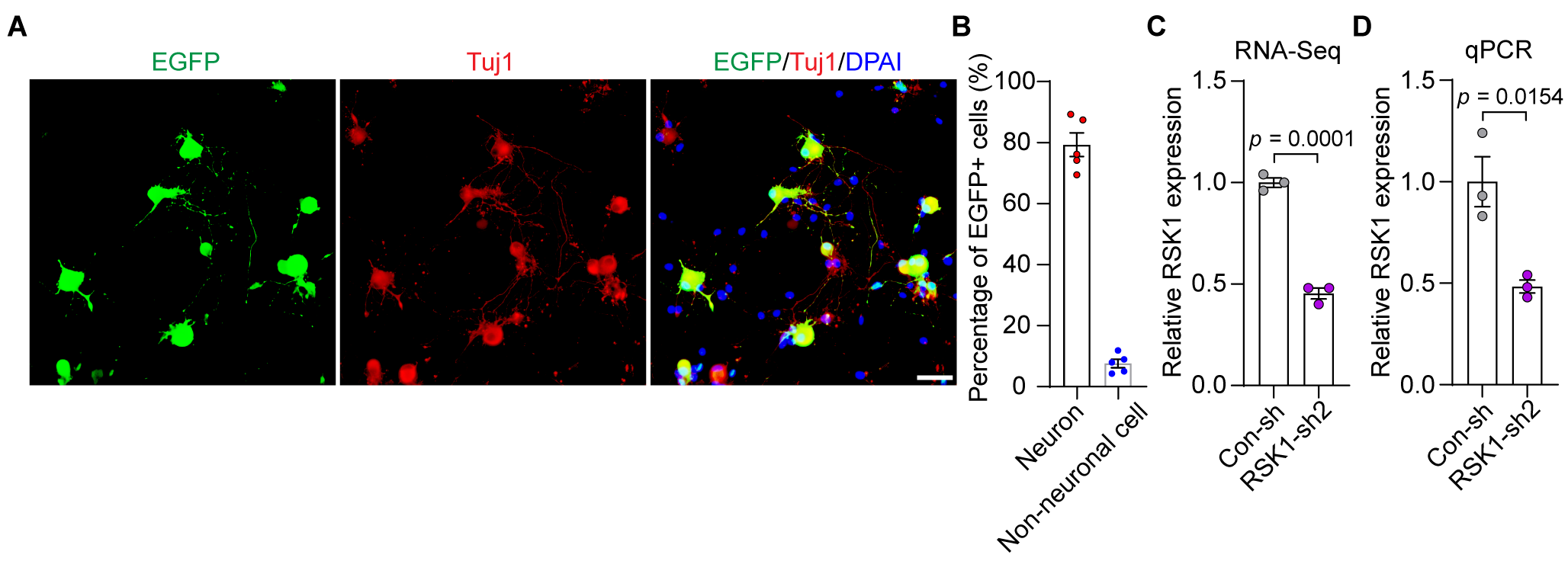

Supplement: S9 Fig — Related to Fig 6. (A) EGFP (green) was co-labeled with a neuronal marker Tuj1 (red) and a nuclear staining dye DAPI (blue) in primary DRG neurons at 7 days following infection of AAV2/8 expressing shRNA2 targeting RSK1 (RSK1-sh2). Scale bar, 50 μm. (B) Bar graph represents percentages of EGFP-positive cells in neurons or nonneuronal cells (mean ± SEM, n = 5 biologically independent wells). (C, D) RNA-seq (C) and RT-qPCR (D) analysis of the expression of RSK1 in primary DRG neurons infected with control AAV2/8 expressing scramble shRNA (Con-sh) or AAV expressing RSK1-sh2 (mean ± SEM, unpaired 2-tailed t test, n = 3 biologically independent experiments). The data underlying all the graphs shown in the figure are included in S1 Data. RSK1, ribosomal S6 kinase 1; DRG, dorsal root ganglion; RT-qPCR, reverse transcription quantitative real-time PCR; SEM, standard error of the mean. (TIF) [file pbio.3001653.s009.tif]

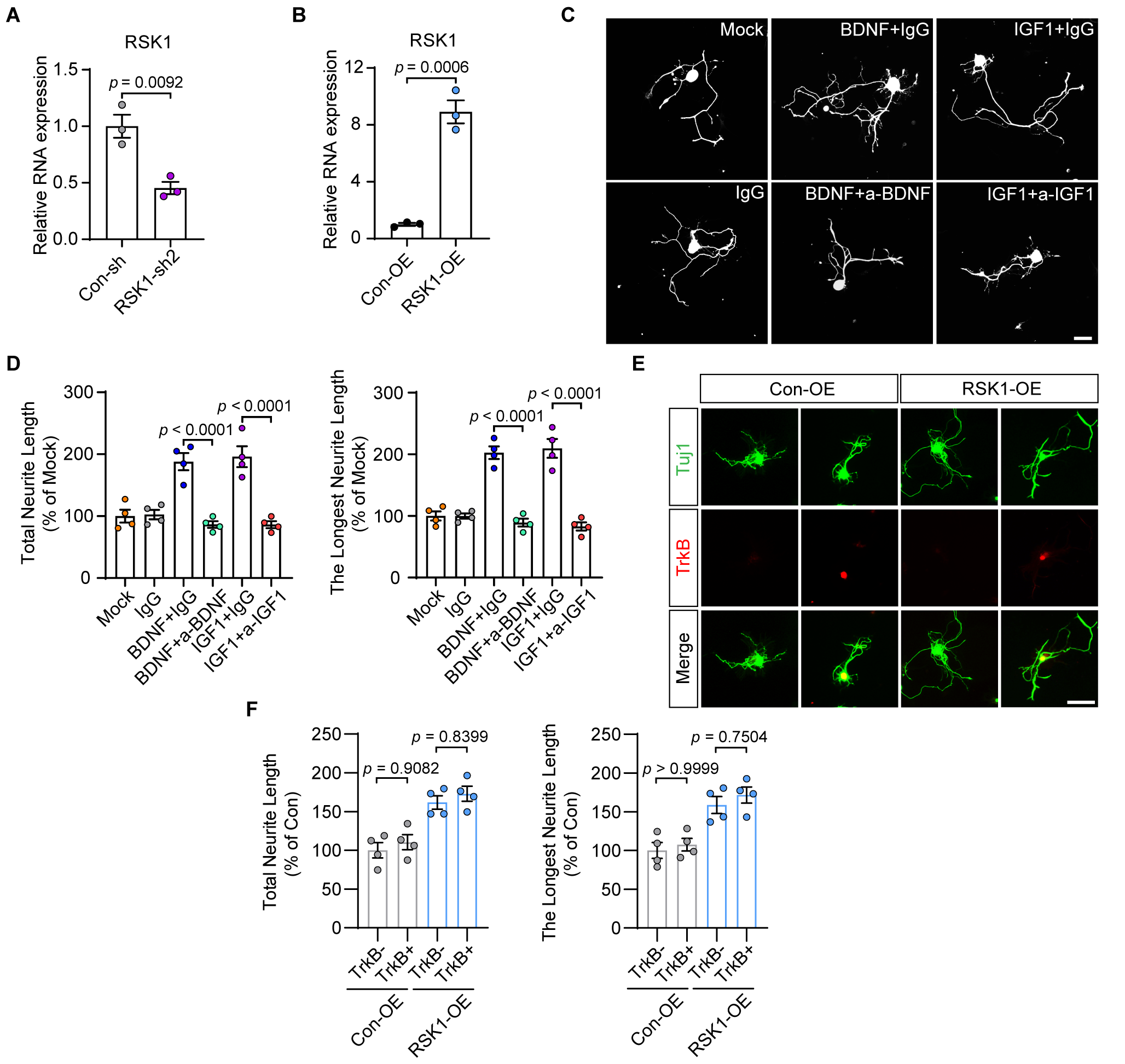

Supplement: S10 Fig — Related to Fig 7. (A) RT-qPCR analysis of the expression of RSK1 in primary DRG neurons infected with control AAV2/8 expressing scramble shRNA (Con-sh) or AAV expressing shRNA2 (RSK1-sh2) (mean ± SEM, unpaired 2-tailed t test, n = 3 biologically independent experiments). (B) RT-qPCR analysis of the expression of RSK1 in primary DRG neurons infected with control AAV2/8 (Con-OE) or AAV overexpressing RSK1 (RSK1-OE) (mean ± SEM, unpaired 2-tailed t test, n = 3 biologically independent experiments). (C) Representative images of cultured DRG neurons treated with PBS (Mock), 10 μg/mL IgG, 5 ng/mL BDNF and 10 μg/mL IgG (BDNF+IgG), 5 ng/mL BDNF and 10 μg/mL BDNF neutralizing antibody (BDNF+a-BDNF), 10 ng/mL IGF1 and 10 μg/mL IgG (IGF1+IgG), 10 ng/mL IGF1 and 10 μg/mL IGF1 neutralizing antibody (IGF1+a-IGF1). Scale bar, 50 μm. (D) Quantification of the total and the longest neurite outgrowth per neuron relating to (C) (mean ± SEM, 1-way ANOVA, Tukey post hoc test, n = 4 biologically independent experiments, approximately 50 cells/experiment on average). (E) Representative images of cultured DRG neurons infected with control AAV2/8 (Con-OE) or AAV overexpressing RSK1 (RSK1-OE). Red signals show the TrkB+ cells by FISH, whereas the green signals show the Tuj1+ cells by IHC. Scale bar, 100 μm. (F) Quantification of the total and the longest neurite outgrowth per neuron relating to (E) (mean ± SEM, 1-way ANOVA, Bonferroni post hoc test, n = 4 biologically independent experiments, approximately 50 cells/experiment on average). The data underlying all the graphs shown in the figure are included in S1 Data. DRG, dorsal root ganglion; RSK1, ribosomal S6 kinase 1; RT-qPCR, reverse transcription quantitative real-time PCR; SEM, standard error of the mean. (TIF) [file pbio.3001653.s010.tif]

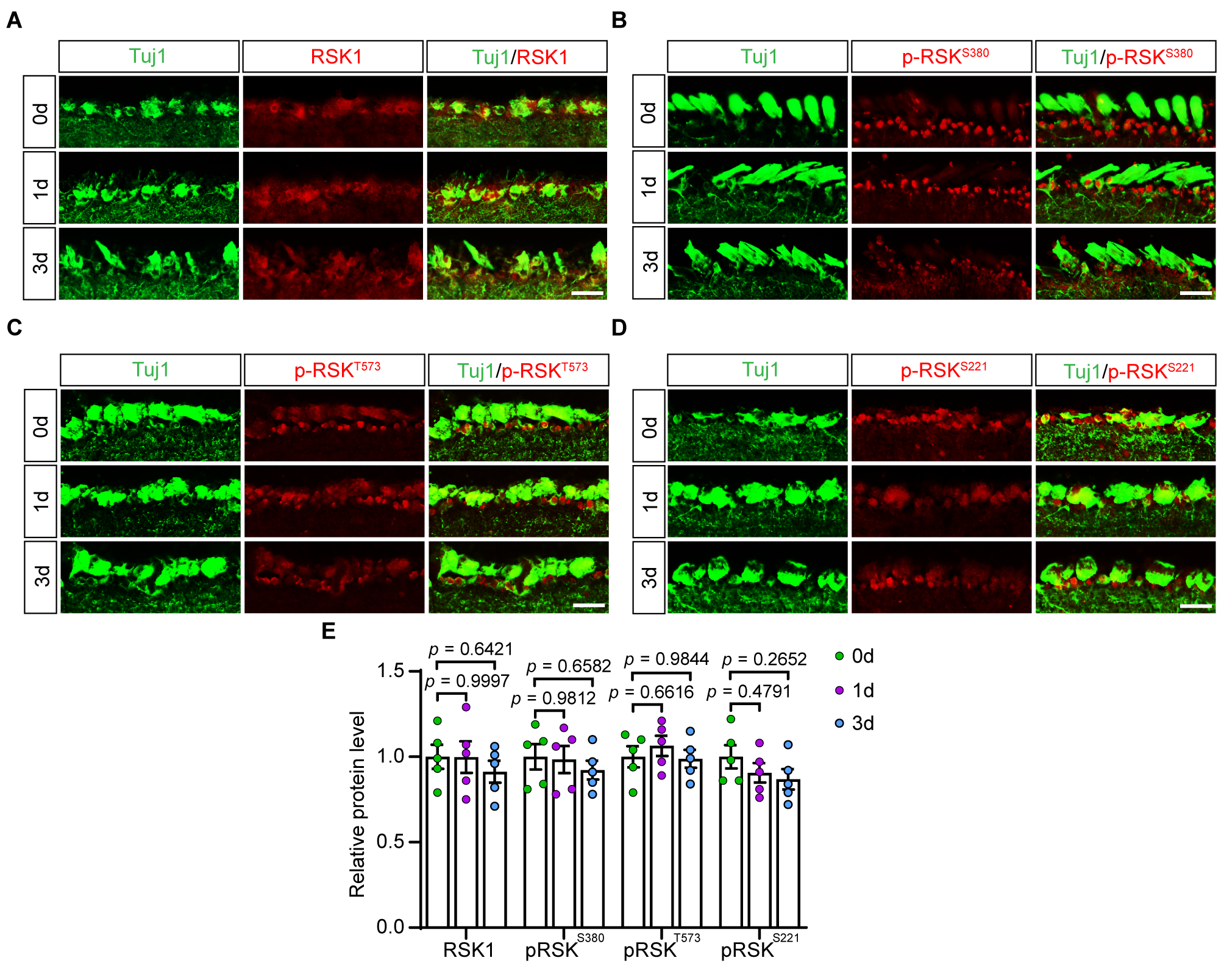

Supplement: S11 Fig — Related to Fig 8. (A–D) Representative fluorescence images of immunostaining for RSK1 (A), p-RSKS380 (B), p-RSKT573 (C), and p-RSKS221 (D) (red) in the retina at 0, 1 or 3 days post-ONC injury. Tuj1 (green) was used to label RGCs. Scale bar, 40 μm. (E) Quantification of RSK1, p-RSKS380, p-RSKT573, and p-RSKS221 immunofluorescence intensity relating to (A–D), respectively. Relative protein expression levels were quantified after normalization to background immunofluorescence (secondary antibody only) (mean ± SEM, 1-way ANOVA, Dunnett post hoc test, n = 5 biologically independent animals/group). The data underlying all the graphs shown in the figure are included in S1 Data. ONC, optic nerve crush; RGC, retinal ganglion cell; RSK1, ribosomal S6 kinase 1; SEM, standard error of the mean. (TIF) [file pbio.3001653.s011.tif]

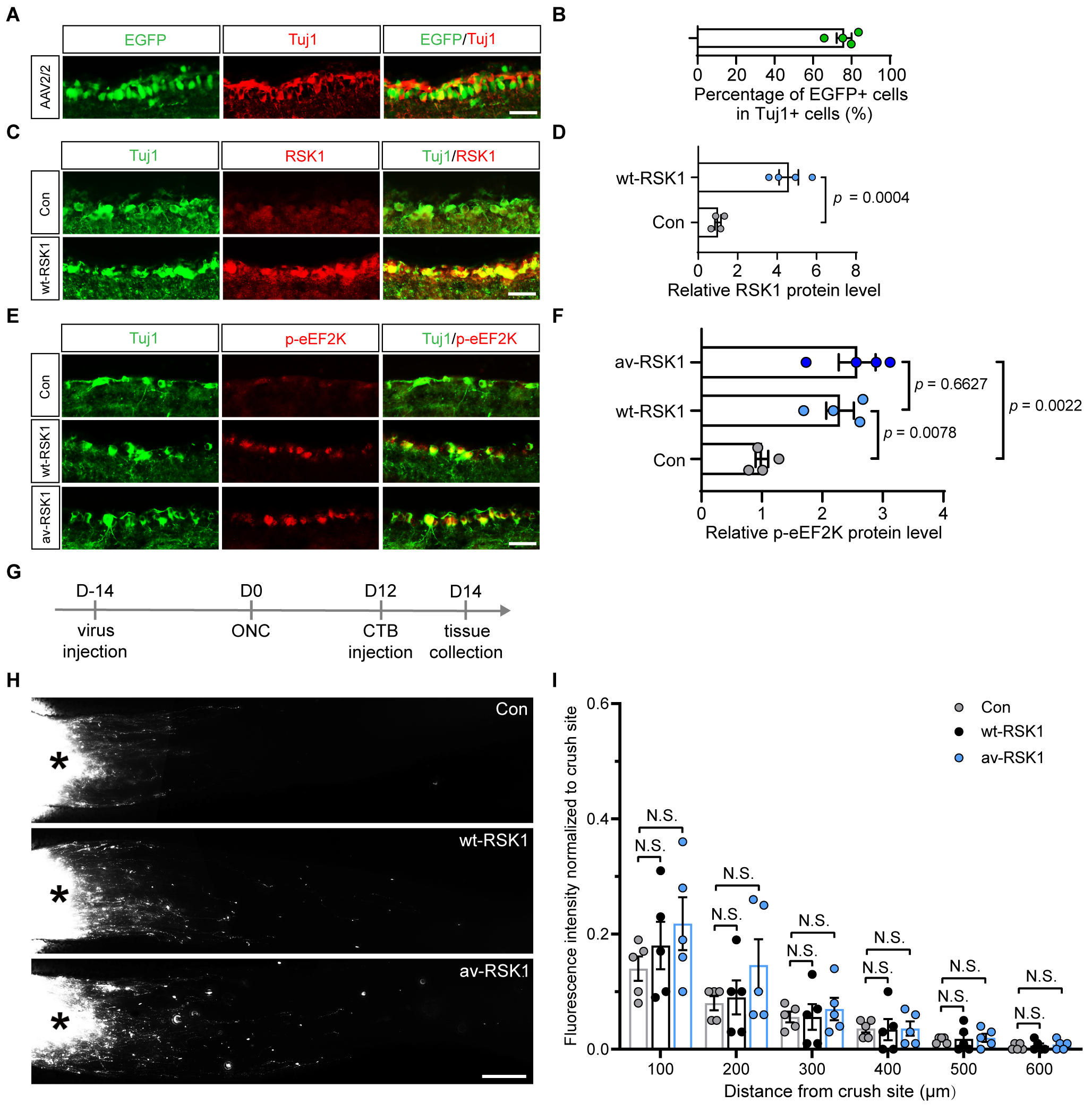

Supplement: S12 Fig — Related to Fig 8. (A) EGFP (green) was co-labeled with a RGC marker Tuj1 (red) in rat retinas at 2 weeks following intravitreal injection of AAV2 expressing EGFP. Scale bar, 40 μm. (B) Bar graph represents percentage of EGFP-positive neurons in RGCs (mean ± SEM, n = 4 biologically independent animals). (C) Representative fluorescence images of Tuj1 (green) and RSK1 (red) in the retina infected with control AAV2 (Con), or AAV expressing wt-RSK1. Scale bar, 40 μm. (D) Quantification of RSK1 immunofluorescence intensity in RGCs relating to (C). Relative protein expression levels were quantified after normalization to background immunofluorescence (secondary antibody only) (mean ± SEM, unpaired 2-tailed t test, n = 4 biologically independent animals/group). (E) Representative fluorescence images of Tuj1 (green) and p-eEF2K (red) in the retina infected with control AAV2 (Con), AAVs expressing wt-RSK1 or active mutant (S221D, S380D, and T573D) RSK1 (av-RSK1). Scale bar, 40 μm. (F) Quantification of p-eEF2K immunofluorescence intensity in RGCs relating to (E) (mean ± SEM, 1-way ANOVA, Tukey post hoc test, n = 4 biologically independent animals/group). (G) Timeline for RSK1 overexpression in rat RGCs, ONC injury, and CTB injection. (H) Representative images of the cleared whole-mount rat optic nerves 2 weeks postinjury. Control AAV2 (Con), AAVs expressing wt-RSK1 or av-RSK1 were administered by intravitreal injection. Axons were labeled by AF 555-conjugated CTB. Scale bar, 250 μm. (I) Normalized fluorescence intensity plotted in function of the distance from the crush line (N.S., not significant, mean ± SEM, 2-way ANOVA, Tukey post hoc test, n = 5 rats per group). The data underlying all the graphs shown in the figure are included in S1 Data. AF, Alexa Fluor; CTB, cholera toxin B subunit; ONC, optic nerve crush; RGC, retinal ganglion cell; RSK1, ribosomal S6 kinase 1; SEM, standard error of the mean; wt-RSK1, wild-type RSK1. (TIF) [file pbio.3001653.s012.tif]

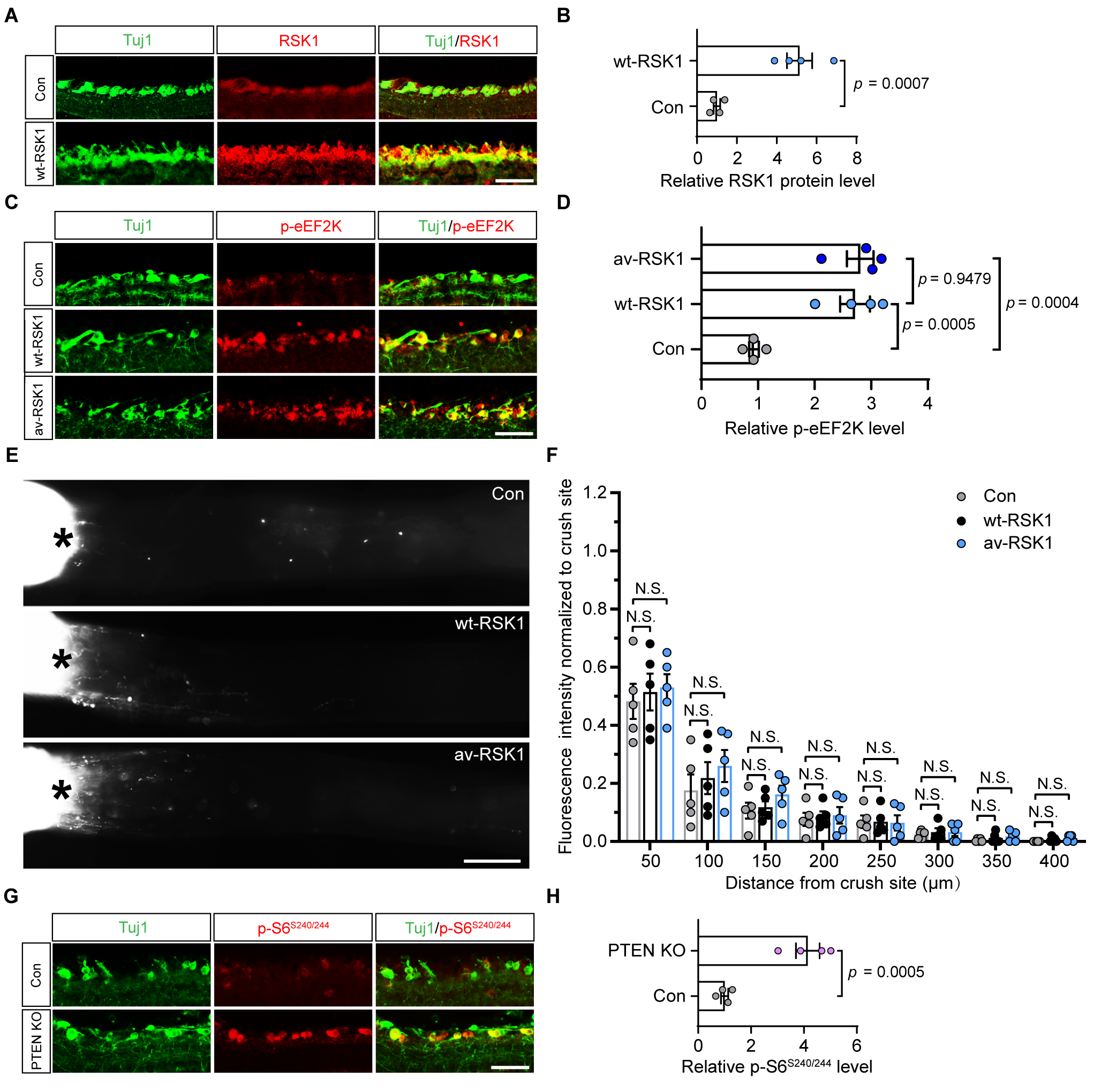

Supplement: S13 Fig — Related to Fig 8. (A) Representative fluorescence images of Tuj1 (green) and RSK1 (red) in mouse retina infected with control AAV2 (Con), or AAV expressing wt-RSK1. Scale bar, 40 μm. (B) Quantification of RSK1 immunofluorescence intensity in mouse RGCs relating to (A). Relative protein expression levels were quantified after normalization to background immunofluorescence (secondary antibody only) (mean ± SEM, unpaired 2-tailed t test, n = 4 biologically independent animals/group). (C) Representative fluorescence images of Tuj1 (green) and p-eEF2K (red) in mouse retina infected with control AAV2 (Con), AAV expressing wt-RSK1or av-RSK1. Scale bar, 40 μm. (D) Quantification of p-eEF2K immunofluorescence intensity in RGCs relating to (C) (mean ± SEM, 1-way ANOVA, Tukey post hoc test, n = 4 biologically independent animals/group). (E) Representative images of the cleared whole-mount mouse optic nerves 2 weeks postinjury. Control AAV2 (Con), AAVs expressing wt-RSK1 or av-RSK1 were administered by intravitreal injection. Axons were labeled by AF 555-conjugated CTB. Scale bar, 250 μm. (F) Normalized fluorescence intensity plotted in function of the distance from the crush line (N.S., not significant, mean ± SEM, 2-way ANOVA, Tukey post hoc test, n = 5 mice per group). (G) Representative fluorescence images of Tuj1 (green) and p-S6S240/244 (red) in Rosa26-Cas-9 mouse retina infected with control AAV2 (Con), or AAV with gRNA targeting PTEN (PTEN KO) followed by ONC for 2 weeks. Scale bar, 40 μm. (H) Quantification of p-S6S240/244 immunofluorescence intensity in RGCs relating to (G) (mean ± SEM, unpaired 2-tailed t test, n = 4 biologically independent animals/group). The data underlying all the graphs shown in the figure are included in S1 Data. AF, Alexa Fluor; CTB, cholera toxin B subunit; gRNA, guide RNA; ONC, optic nerve crush; RGC, retinal ganglion cell; RSK1, ribosomal S6 kinase 1; SEM, standard error of the mean; wt-RSK1, wild-type RSK1. (TIF) [file pbio.3001653.s013.tif]
