# Supplementary material for: RSK1 promotes mammalian axon regeneration by inducing the synthesis of regeneration-related proteins
Source: PLoS Biol. 2022 Jun 1;20(6):e3001653. doi: 10.1371/journal.pbio.3001653 (PMC9159620; doi:10.1371/journal.pbio.3001653)
Supplement: S1 Raw images — (PPTX) [file pbio.3001653.s015.pptx]

## Slide 1
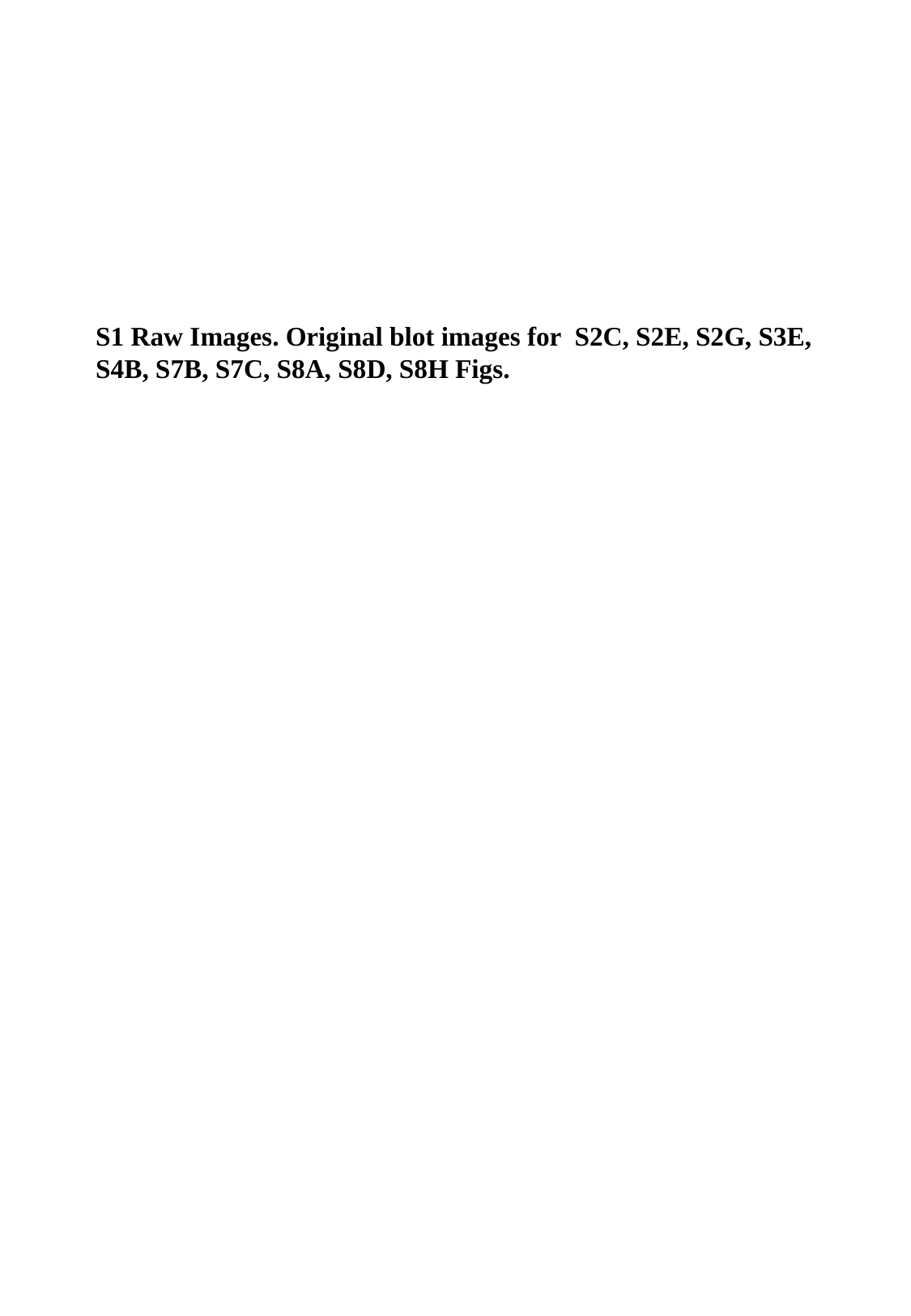

S1 Raw Images. Original blot images for S2C, S2E, S2G, S3E, S4B, S7B, S7C, S8A, S8D, S8H Figs.

## Slide 2
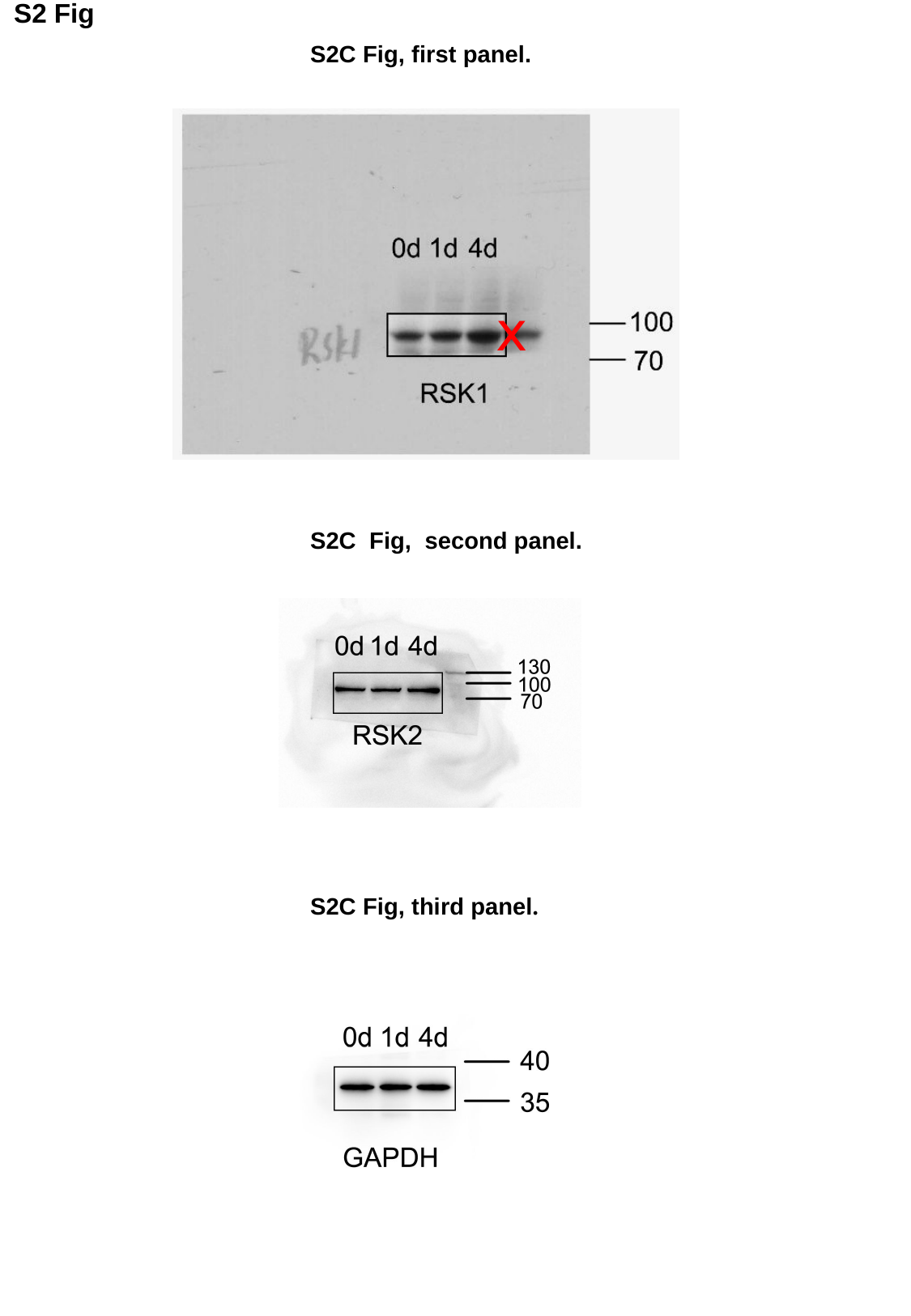

S2 Fig
S2C Fig, first panel.
ⅹ
S2C Fig, second panel.
S2C Fig, third panel.

## Slide 3
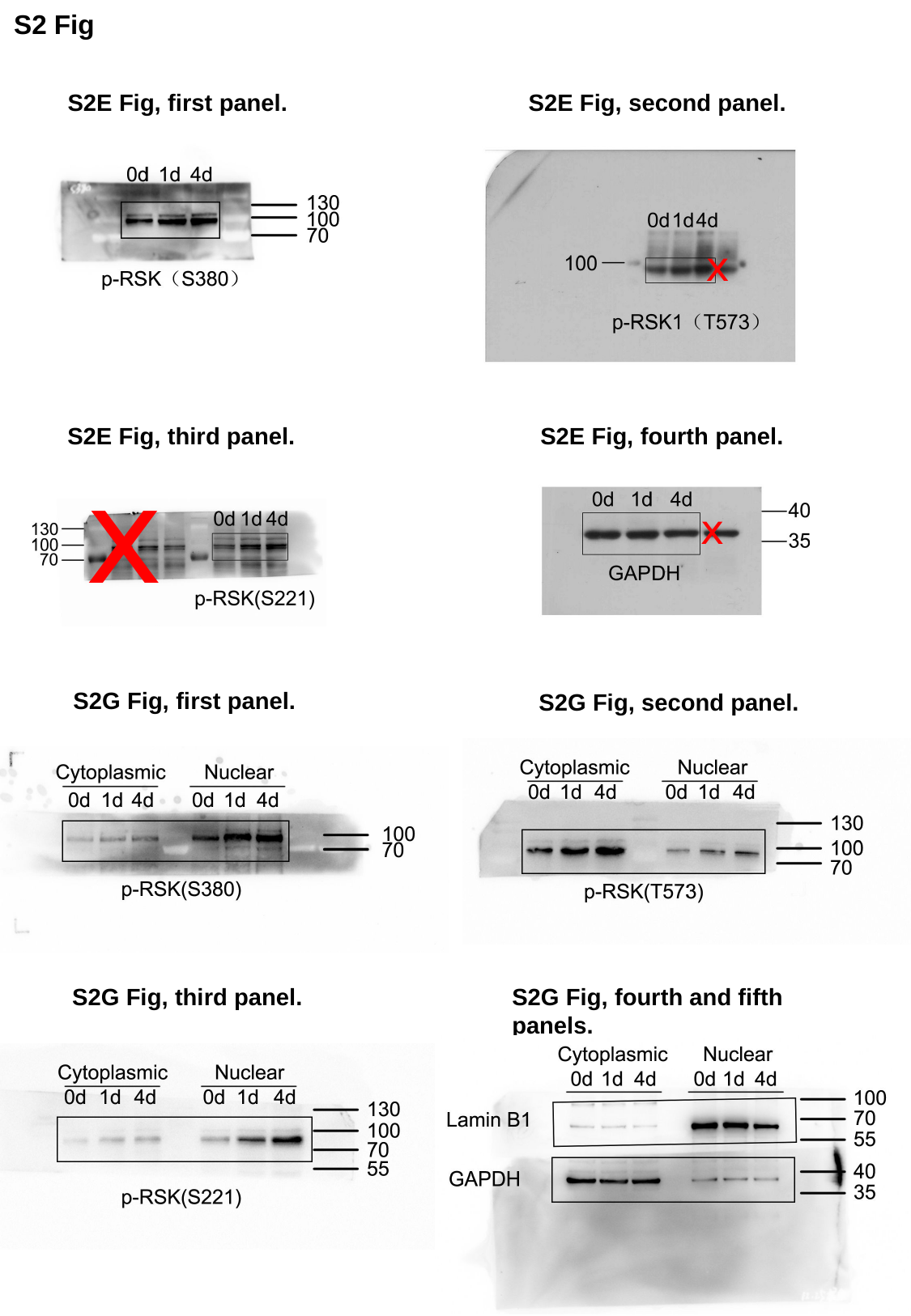

S2 Fig
S2E Fig, first panel.
S2E Fig, second panel.
ⅹ
S2E Fig, third panel.
S2E Fig, fourth panel.
ⅹ
ⅹ
S2G Fig, first panel.
S2G Fig, second panel.
S2G Fig, third panel.
S2G Fig, fourth and fifth panels.

## Slide 4
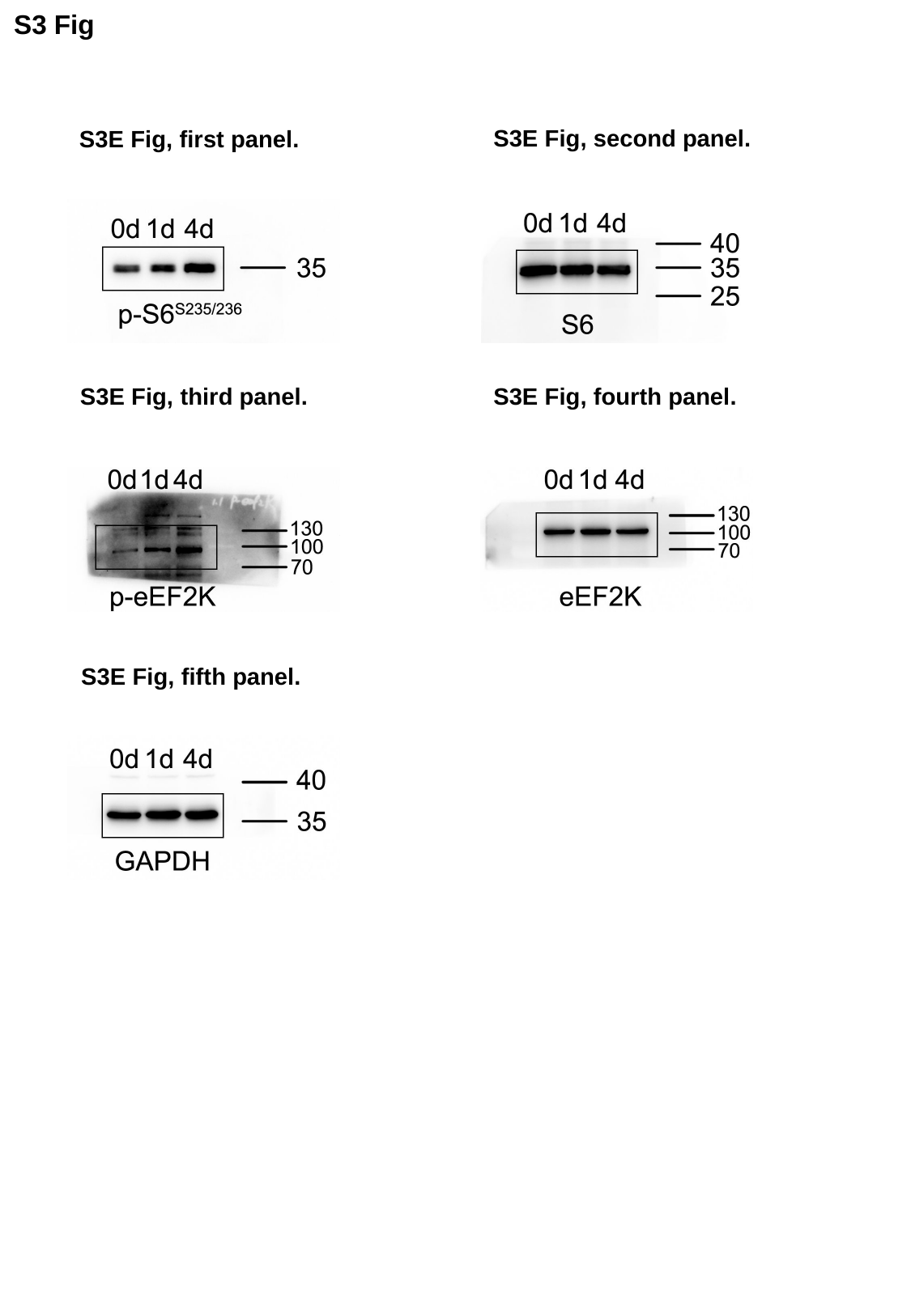

S3 Fig
S3E Fig, second panel.
S3E Fig, first panel.
S3E Fig, fourth panel.
S3E Fig, third panel.
S3E Fig, fifth panel.

## Slide 5
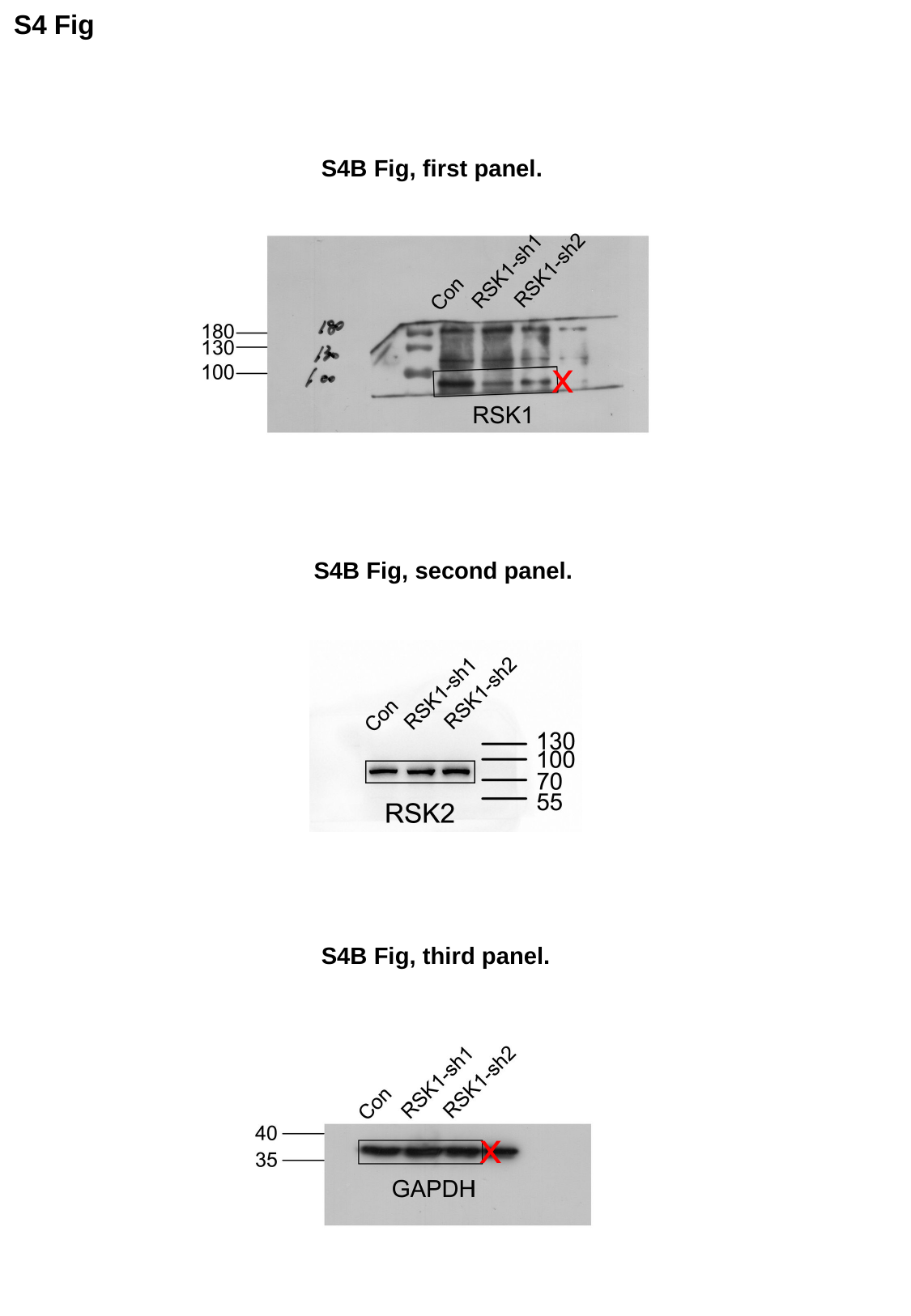

S4 Fig
S4B Fig, first panel.
ⅹ
S4B Fig, second panel.
S4B Fig, third panel.
ⅹ

## Slide 6
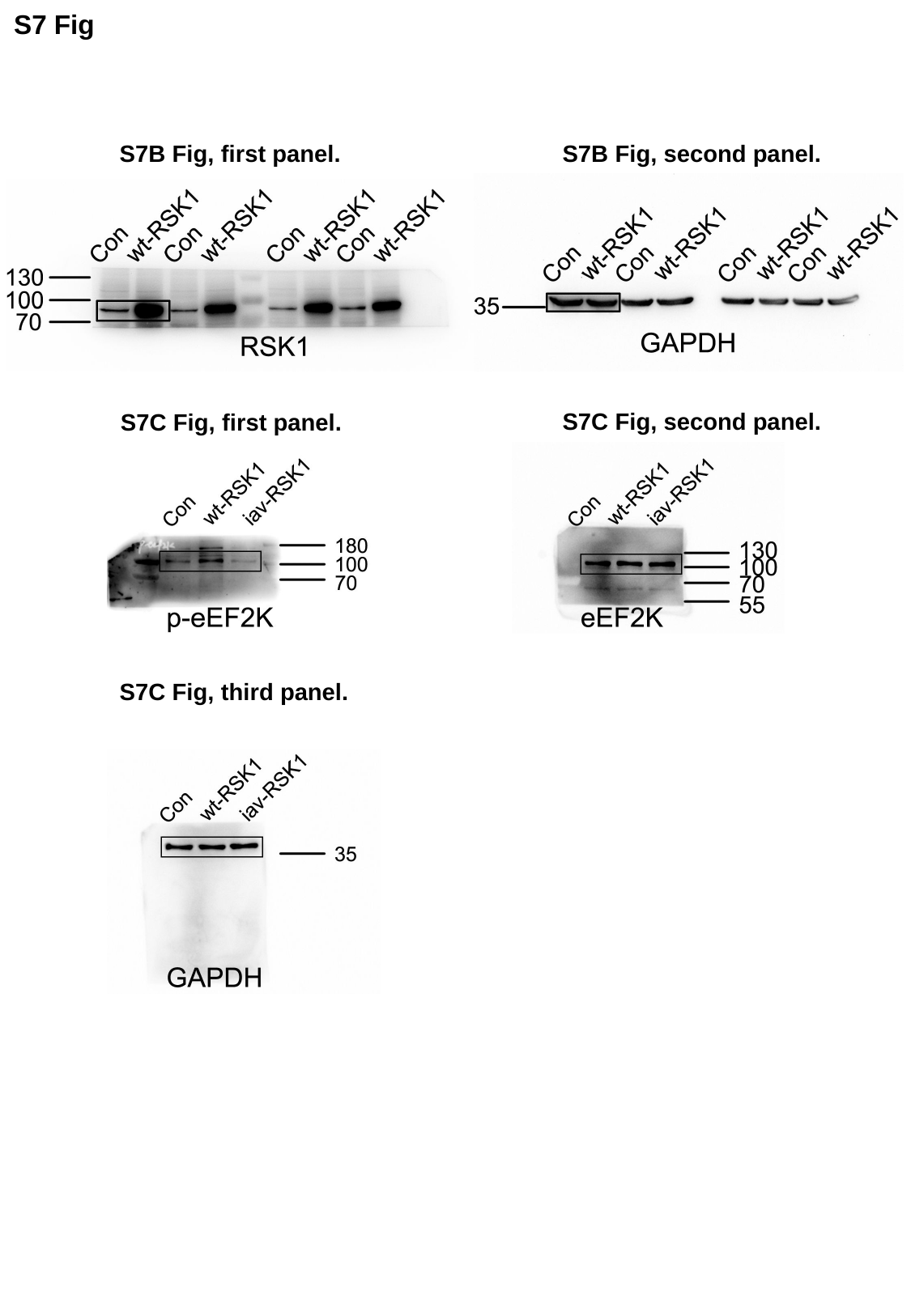

S7 Fig
S7B Fig, first panel.
S7B Fig, second panel.
S7C Fig, second panel.
S7C Fig, first panel.
S7C Fig, third panel.

## Slide 7
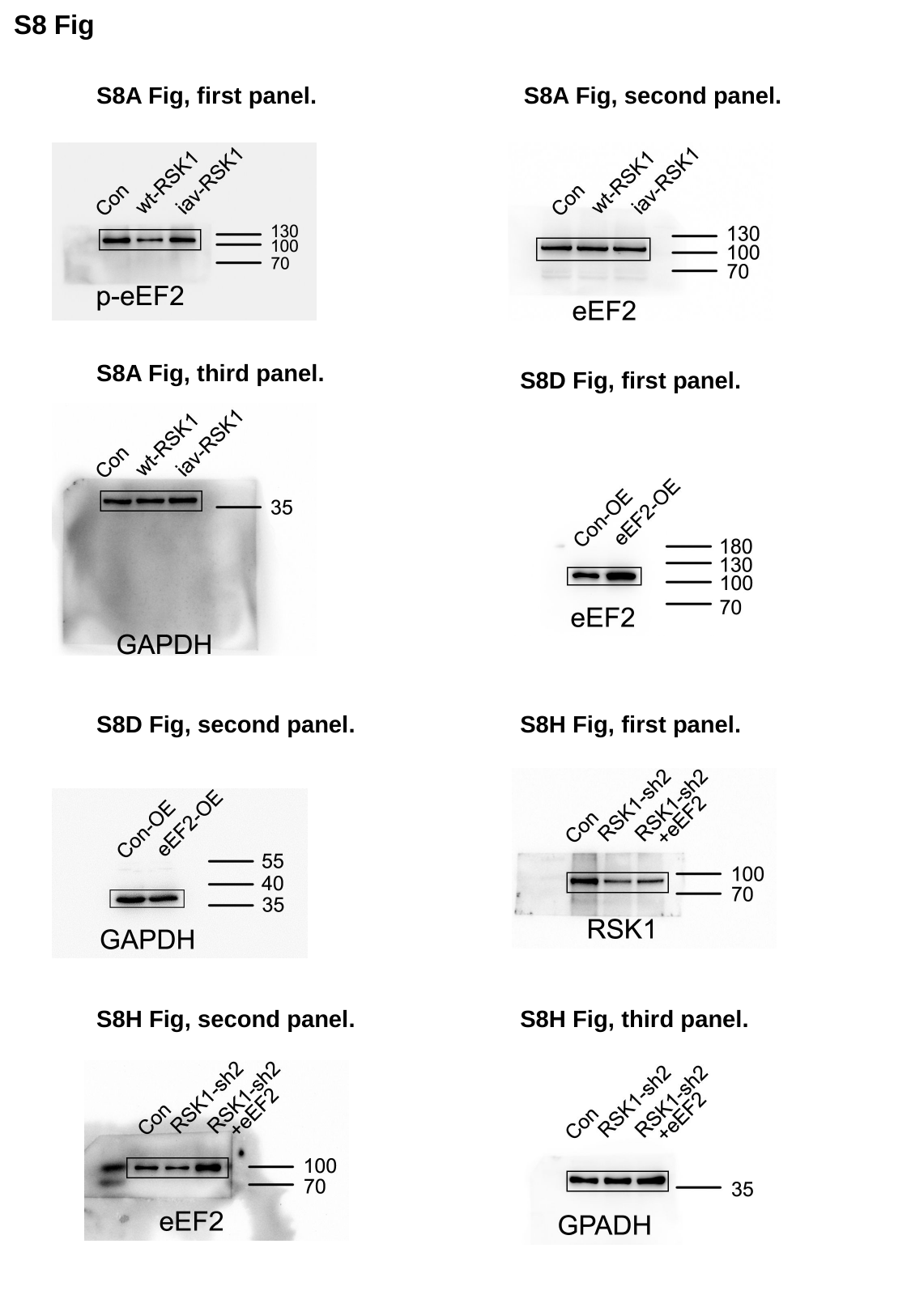

S8 Fig
S8A Fig, second panel.
S8A Fig, first panel.
S8A Fig, third panel.
S8D Fig, first panel.
S8H Fig, first panel.
S8D Fig, second panel.
S8H Fig, second panel.
S8H Fig, third panel.
