# Supplementary material for: RSK1 promotes mammalian axon regeneration by inducing the synthesis of regeneration-related proteins
Source: PLoS Biol. 2022 Jun 1;20(6):e3001653. doi: 10.1371/journal.pbio.3001653 (PMC9159620; doi:10.1371/journal.pbio.3001653)
Supplement: S5 Table — (DOCX) [file pbio.3001653.s020.docx]

**S5 Table. List of shRNA and sgRNA sequences.**

| **Name** | **Sequence** |
| --- | --- |
| Control shRNA | CCTAAGGTTAAGTCGCCCTCG |
| Rsk1-sh1 | GCAAGACTGTGGAATACTTGC |
| Rsk1-sh2 | GGATCACCCAGAAAGACAAGC |
| PTEN sgRNA1 | ACCGCCAAATTTAACTGCAG |
| PTEN sgRNA2 | GCAGCAATTCACTGTAAAGC |
| PTEN sgRNA3 | TGTCATCTTCACTTAGCCAT |
| PTEN sgRNA4 | ACAATATTGATGATGTAGTA |
| PTEN sgRNA5 | CATACCTCTGCAGTTAAATT |
