# Supplementary material for: RSK1 promotes mammalian axon regeneration by inducing the synthesis of regeneration-related proteins
Source: PLoS Biol. 2022 Jun 1;20(6):e3001653. doi: 10.1371/journal.pbio.3001653 (PMC9159620; doi:10.1371/journal.pbio.3001653)
Supplement: S6 Table — (DOCX) [file pbio.3001653.s021.docx]

**S6 Table. List of qRT-PCR primers.**

| **Gene symbol** |  | **Sequence (5’-3’)** |
| --- | --- | --- |
| RSK1 | Forward | AAGCTGGACTTCAGCCATCC |
|  | Reverse | GAACACGGTCACGCACTTTC |
| RSK2 | Forward | CTGCTCCTGCTTCGTCTC |
|  | Reverse | CATAAACTGTCCATCCCTGTAA |
| CACNA1S | Forward | TTCCCAGGAATTCGGCTCAC |
|  | Reverse | CCCTTTGGGGATTCTCGCTT |
| EXOC2 | Forward | GAAGCAGACGGAACAGAA |
|  | Reverse | TTGAGAGGAAGGTTGAAGAG |
| PRKCA | Forward | GAAGGCAGAGGTCACAGATGAA |
|  | Reverse | CAGTCGCCGGTCTTTGTCT |
| BDNF | Forward | ACTCTGGAGAGCGTGAATGG |
|  | Reverse | GCAGCCTTCCTTCGTGTAAC |
| IGF1 | Forward | TCTCTTCTACCTGGCACTCTG |
|  | Reverse | GAAGCAACACTCATCCACAAT |
| eEF2 | Forward | CCACTAAGGAGGGTGCTCTT |
|  | Reverse | ACACCATAGATTCCGCCCAC |
| GAPDH | Forward | ATGCCATCACTGCCACTCA |
|  | Reverse | CCTGCTTCACCACCTTCTTG |
