# Supplementary material for: RSK1 promotes mammalian axon regeneration by inducing the synthesis of regeneration-related proteins
Source: PLoS Biol. 2022 Jun 1;20(6):e3001653. doi: 10.1371/journal.pbio.3001653 (PMC9159620; doi:10.1371/journal.pbio.3001653)
Supplement: S7 Table — (DOCX) [file pbio.3001653.s022.docx]

**S7 Table. List of cDNAs used for in situ hybridization.**

| **Gene symbol** | **Genbank Accession Numbers** | **Position** | **Size (bp)** |
| --- | --- | --- | --- |
| RSK1 | NM_031107.2 | 1367-2274 | 908 |
| RSK2 | [NM_001192004.3](https://www.ncbi.nlm.nih.gov/entrez/viewer.fcgi?db=nucleotide&id=1982559700) | 943-1675 | 733 |
| RSK3 | [NM_057128.1](https://www.ncbi.nlm.nih.gov/entrez/viewer.fcgi?db=nucleotide&id=281306813) | 2193-3087 | 895 |
| RSK4 | [NM_001191721.1](https://www.ncbi.nlm.nih.gov/entrez/viewer.fcgi?db=nucleotide&id=300795099) | 1480-2279 | 800 |
