# Supplementary material for: RSK1 promotes mammalian axon regeneration by inducing the synthesis of regeneration-related proteins
Source: PLoS Biol. 2022 Jun 1;20(6):e3001653. doi: 10.1371/journal.pbio.3001653 (PMC9159620; doi:10.1371/journal.pbio.3001653)
Supplement: S8 Table — (DOCX) [file pbio.3001653.s023.docx]

**S8 Table. List of the antibodies.**

| **Antibody** | **Dilution** | **Source** | **Identifier** |
| --- | --- | --- | --- |
| Rabbit polyclonal to beta III Tubulin | 1:1000 (ICC)  1:250 (IHC) | Abcam | ab18207 |
| Mouse monoclonal to beta III Tubulin | 1:250 (IHC) | R&D systems | MAB1195 |
| Rabbit monoclonal [E4] to RSK1 | 1:100 (IHC)  1:500 (WB) | Abcam | ab32114 |
| Rabbit monoclonal [D21B2] to RSK2 | 1:100 (IHC)  1:500 (WB) | CST | #5528 |
| Mouse monoclonal [A60] to NeuN | 1:100 (IHC) | Sigma-Aldrich | MAB377 |
| Rabbit monoclonal [E239] to RSK1 (phospho S380) | 1:100 (IHC)  1:500 (WB) | Abcam | ab32203 |
| Rabbit polyclonal to RSK1 (phospho S221)/ RSK2 (phospho S227) | 1:100 (IHC)  1:500 (WB) | R&D systems | AF892 |
| Rabbit monoclonal [EP2133Y] to RSK1 (phospho T573) | 1:100 (IHC)  1:500 (WB) | Abcam | ab62324 |
| Rabbit polyclonal to SCG10 | 1:400 (IHC) | Novus | NBP1-49461 |
| Rabbit polyclonal to eEF2 | 1:500 (WB)  1:100 (IHC) | CST | #2332 |
| Rabbit polyclonal to EEF2 (phospho T56) | 1:100 (IHC)  1:500 (WB) | Abcam | ab53114 |
| Rabbit polyclonal to eEF2K | 1:500 (WB) | CST | #3692 |
| Mouse monoclonal to p-eEF2K (phospho S366) | 1:100 (IHC)  1:500 (WB) | Santa Cruz | sc-377536 |
| Mouse monoclonal [N52] to NF200 | 1:200 (IHC) | Sigma-Aldrich | MAB5266 |
| Mouse monoclonal [4901] to CGRP | 1:100 (IHC) | Sigma-Aldrich | C7113 |
| Isolectin B4 (BSI-B4), biotin conjugate | 1:100 (IHC) | Sigma-Aldrich | L-2140 |
| Rabbit monoclonal [5G10] to S6 | 1:1000 (WB) | CST | #2217 |
| Rabbit monoclonal [D57.2.2E] to S6 (phospho Ser235/236) | 1:100 (IHC)  1:500 (WB) | CST | #4858 |
| Rabbit monoclonal [D68F8] to S6 (phospho Ser240/244) | 1:100 (IHC)  1:500 (WB) | CST | #5364 |
| Rabbit monoclonal [D9V6H] to Lamin B1 | 1:1000 (WB) | CST | #13435 |
| Rabbit polyclonal to BDNF | 10 μg/ml | LifeSpan BioSciences | LS-C104728 |
| Mouse monoclonal [SPM406] to IGF1 | 10 μg/ml | Novus | NBP2-34409 |
| Rabbit monoclonal [EPR16884] to GAPDH | 1:3000 (WB) | Abcam | ab181603 |
| Alexa Fluor 594 goat anti-rabbit IgG (H+L) | 1:1000 | ThermoFisher | A11012 |
| Alexa Fluor 488 donkey anti-rabbit IgG (H+L) | 1:1000 | ThermoFisher | A21206 |
| Alexa Fluor 488 goat anti- mouse IgG (H+L) | 1:1000 | ThermoFisher | A11001 |
| Alexa Fluor 594 donkey anti-Mouse IgG (H+L) | 1:1000 | ThermoFisher | A21203 |
| Alexa Fluor 647 goat anti-Rabbit IgG (H+L) | 1:1000 | ThermoFisher | A21244 |
| Alexa Fluor 647 donkey anti-Mouse IgG (H+L) | 1:1000 | ThermoFisher | A32787 |
| Peroxidase AffiniPure Goat Anti-Mouse IgG (H+L) | 1:5000 | Jackson | 115-035-003 |
| Peroxidase AffiniPure Goat Anti-Rabbit IgG (H+L) | 1:5000 | Jackson | 111-035-003 |
| Streptavidin−Cy3 from Streptomyces avidinii | 1:500 | Sigma-Aldrich | S6402 |
